# Click Chemistry of Selenium Dihalides: Novel Bicyclic Organoselenium Compounds Based on Selenenylation/Bis-Functionalization Reactions and Evaluation of Glutathione Peroxidase-like Activity

**DOI:** 10.3390/ijms232415629

**Published:** 2022-12-09

**Authors:** Maxim V. Musalov, Vladimir A. Potapov

**Affiliations:** A. E. Favorsky Irkutsk Institute of Chemistry, Siberian Division of The Russian Academy of Sciences, 1 Favorsky Str., 664033 Irkutsk, Russia

**Keywords:** selenium dihalides, alcohols, 9-selenabicyclo[3.3.1]nonane derivatives, glutathione peroxidase-like activity

## Abstract

A number of highly efficient methods for the preparation of novel derivatives of 9-selenabicyclo[3.3.1]nonane in high yields based on selenium dibromide and cis,cis-1,5-cyclooctadiene are reported. The one-pot syntheses of 2,6-diorganyloxy-9-selenabicyclo[3.3.1]nonanes using various O-nucleophiles including alkanols, phenols, benzyl, allyl, and propargyl alcohols were developed. New 2,6-bis(1,2,3-triazol-1-yl)-9-selenabicyclo[3.3.1]nonanes were obtained by the copper-catalyzed 1,3-dipolar cycloaddition of 2,6-diazido-9-selenabicyclo[3.3.1]nonane with unsubstituted gaseous acetylene and propargyl alcohol. The synthesis of 2,6-bis(vinylsulfanyl)-9-selenabicyclo[3.3.1]nonane, based on the generation of corresponding dithiolate anion from bis[amino(iminio)methylsulfanyl]-9-selenabicyclo[3.3.1]nonane dibromide, followed by the nucleophilic addition of the dithiolate anion to unsubstituted acetylene, was developed. The glutathione peroxidase-like activity of the obtained water-soluble products was estimated and compounds with high activity were found. Overall, 2,6-Diazido-9-selenabicyclo[3.3.1]nonane exhibits the highest activity among the obtained compounds.

## 1. Introduction

Selenium and selenium-containing compounds were considered poisons for many years, until this element was identified as a micronutrient for humans and mammals [1]. Since then, interest in the synthesis and properties of organoselenium compounds has increased significantly [2,3,4,5,6]. 

Organoselenium compounds, and particularly selenium heterocycles, exhibit various kinds of biological activity, including antitumor, antiviral, antibacterial, anti-inflammatory, antiproliferative, antifungal, and glutathione peroxidase-like properties [7,8,9,10,11,12,13,14,15,16,17,18,19,20,21,22,23,24,25,26,27,28,29,30,31,32,33]. 

**Figure 1:**
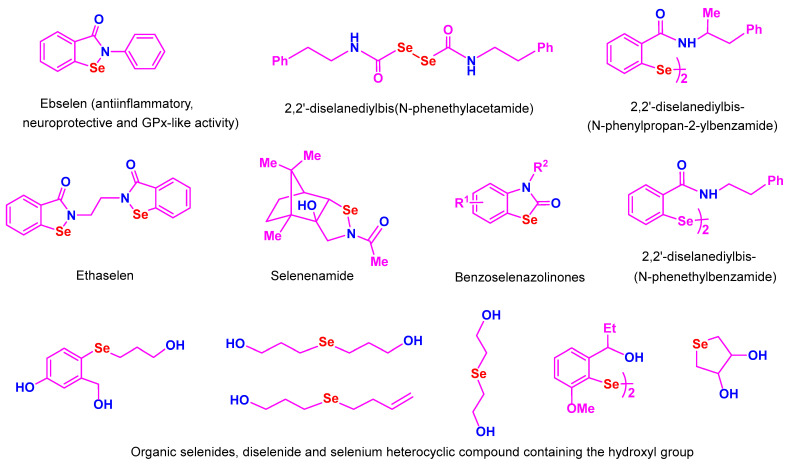
Examples of known functionalized organoselenium compounds with glutathione peroxidase-like activity including selenides with the hydroxyl groups [26,27,28,29,30,31,32,33].

The glutathione peroxidase-like activity is perhaps the most important biological property of organoselenium compounds. It is known that a number of organoselenium compounds exhibit glutathione peroxidase-like activity and, in trace amounts, can potentially act in the human body as catalysts for the reduction in peroxides and other reactive oxygen species, preventing lipid peroxidation and other undesirable processes. Some examples of known functionalized organoselenium compounds with glutathione peroxidase-like activity, including selenides containing the hydroxyl group, are presented in Figure 1 [26,27,28,29,30,31,32,33]. The presence of the hydroxyl group increases the solubility in water, which is considered a desirable property of compounds with glutathione peroxidase-like activity [29,30,31,32,33].

Currently, selenium is recognized as an essential trace element for humans and plays an important role in the human body via selenium-containing enzymes. These enzymes (glutathione peroxidase, thioredoxin reductase, methionine sulfoxide reductase, etc.) are involved in redox regulation in the body, reducing hydrogen peroxide and lipid peroxide species and maintaining antioxidant activity [34,35,36]. Sufficient intake of selenium is very important for the human body. It is known that low or suboptimal levels of selenium intake are associated with a wide range of human diseases such as heart disease, stroke, arthritis, cystic fibrosis, and even several types of cancer [34,35,36]. Selenium supplementation in the elderly is an important strategy for prevention of age-related diseases.

A number of diseases and degenerative conditions are accompanied by particularly high levels of peroxide formation and oxidative stress, which suppresses the protective glutathione peroxidase effect. For example, ischemic reperfusion of infarction in stroke patients often results in cardiovascular and neurological injury from the damaging effects of peroxides and other reactive oxygen species released by neutrophils during the reperfusion process [33,37]. The well-known selenium heterocycle ebselen is used to treat cardiovascular diseases and to prevent ischemic stroke and acute stroke [23,24,25,26,27]. Ebselen is a novel anti-inflammatory drug exhibiting glutathione peroxidase-like and neuroprotective properties. This organoselenium compound has been studied in phase three clinical trials for its cardiovascular and neuroprotective effects [23,24,25,26,27,33]. Moreover, ebselen has recently entered clinical trials in COVID-19 patients as this compound was found to inhibit CoV2 activity and viral replication [24,25]. 

Aging is an inevitable process and is always accompanied by age-related diseases. Reactive oxygen species are important initial factors in aging and age-related diseases. Selenium contributes to reducing inflammation mediated by reactive oxygen species, reducing DNA damage, and plays an important role in the fight against aging and the prevention of age-related diseases [38,39,40]. Thus, there is a growing need to discover new classes of non-toxic water-soluble organoselenium GPx mimetics with high catalytic activity and improved properties. 

Organoselenium compounds have proven themselves as versatile and efficient intermediates and synthons for modern organic synthesis [1,2,3,4,5,6,7,8,9,10,11,41,42,43,44,45,46,47,48,49,50]. The selenium-containing reagents and selenium-mediated reactions are used in the synthesis of many useful products, including the total synthesis of important biologically active molecules [1,2,3,4,5,6,7,8,9,10,11,41,42,43,44,45,46,47,48,49,50].

Earlier in this laboratory, selenium dichloride and dibromide were introduced for the first time into the synthesis of organoselenium compounds [51]. The use of these reagents in organic synthesis made it possible to obtain novel classes of organoselenium and heterocyclic compounds [51,52,53,54,55,56,57,58,59,60,61].

The transannular addition of selenium dichloride and dibromide to cis,cis-1,5-cyclooctadiene afforded 2,6-dichloro-9-selenabicyclo[3.3.1]nonane (**1**) and 2,6-dibromo-9-selenabicyclo[3.3.1]nonane (**2**) in near quantitative yields [56,57,58]. The compound **1** was used in studies of the anchimeric assistance effect of the selenium and sulfur atoms, quantified by the rates of nucleophilic substitution reactions. The anchimeric assistance effect of the selenium atom was found to be approximately two orders of magnitude higher than that of the sulfur atom [56]. Thus, the compounds **1** and **2** are very reactive in nucleophilic substitution reactions and useful reagents for click chemistry. 

**Scheme 1:**
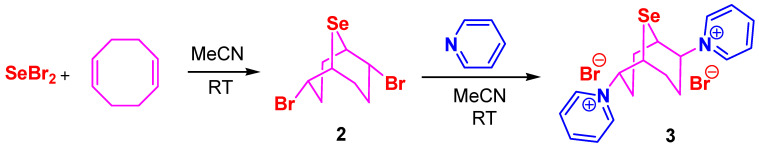
Synthesis of bis-pyridinium salt **3** by the reaction of 2,6-dibromo-9-selenabicyclo[3.3.1]nonane **2** with pyridine.

A number of efficient syntheses of novel organoselenium compounds were developed based on 2,6-dibromo-9-selenabicyclo[3.3.1]nonane (**2**) [56,57,58,59,60,61]. *Inter alia*, bis-pyridinium salt **3** was obtained by the reaction of nonane **2** with pyridine at room temperature in acetonitrile (Scheme 1). 

Joint research of this laboratory with the Irkutsk research anti-plague institute established that compound **3** is a promising drug for metabolic correction during the vaccination process [61]. The introduction of compound **3** into the body of experimental animals significantly decreases the development of pathological reactions under the action of the tularemia vaccine and reduces the reactogenicity of the brucellosis vaccine by one order of magnitude [61]. Moreover, compound **3** does not show toxicity and may act as a catalyst for the decomposition of peroxides in the body, exhibiting glutathione peroxidase-like activity.

It is worthwhile to note that the undesirable post-vaccination reaction of the body is oxidative stress, which develops as a result of the increased generation of reactive oxygen species by cells [61]. It can also lead to inflammatory and allergic reactions, which are based on the process of lipid peroxidation. The development of new drugs with glutathione peroxidase-like activity for metabolic correction is an urgent task, considering the need to vaccinate the population against coronavirus and other diseases. The use of metabolic correction drugs can significantly reduce the side effects that occur during vaccination. 

The recent award of the Nobel Prize to Sharpless, the founder of click chemistry, demonstrates the great importance of this field of organic chemistry [62,63,64,65,66,67,68,69,70,71,72,73,74,75,76,77,78,79,80,81,82,83,84,85,86,87,88,89]. The term “click chemistry” was introduced by Sharpless [62] and is widely used today. Click chemistry reactions should give very high yields of desired products, be broad in scope, and produce only harmless by-products. Available starting materials and reagents, simple reaction conditions, high selectivity, and convenient product isolation procedures are also important features of click chemistry. The classic example of click chemistry is the copper-catalyzed azide-alkynes 1,3-dipolar cycloaddition reaction. The products of this reaction, the 1,2,3-triazole derivatives, exhibit a variety of biological activities [62,63,64,65,66,67,68,69,70,71,72,73,74,75,76,77,78,79,80,81,82,83,84,85,86,87,88,89]. 

The application of organoselenium compounds in click chemistry reactions and the combination of the advantages of selenium-containing reagents with the copper-catalyzed click chemistry of azide-alkynes 1,3-dipolar cycloaddition reactions can give a new impetus to the development of organoselenium chemistry and the synthesis of new useful compounds with high biological activity [90,91]. 

We recently developed the efficient synthesis of bis-1,2,3-triazole derivatives of 9-selenabicyclo[3.3.1]nonane in high yields, combining selenium dihalide click chemistry with the click chemistry of copper-catalyzed azide-alkyne 1,3-dipolar cycloaddition reactions [59]. The copper-catalyzed cycloaddition reaction of 2,6-diazido-9-selenabicyclo[3.3.1]nonane with terminal acetylenes proceeded in a regioselective fashion, affording a number of 2,6-bis(4-organyl-1,2,3-triazole)-9-selenabicyclo[3.3.1]nonanes in high yields.

Thus, the development of efficient and selective methods for the synthesis of new classes of non-toxic water-soluble organoselenium compounds with high glutathione peroxidase-like activity based on the principles of click chemistry is an urgent task for chemists.

## 2. Results and Discussion

The aim of this research is to develop efficient syntheses of novel derivatives of 9-selenabicyclo[3.3.1]nonane by selenenylation/bis-functionalization reactions and nucleophilic substitution with various O-centered nucleophiles (water, alkanols, phenols, benzyl, allyl, and propargyl alcohols) and to estimate glutathione peroxidase-like activity of the obtained water-soluble products. 

**Scheme 2:**
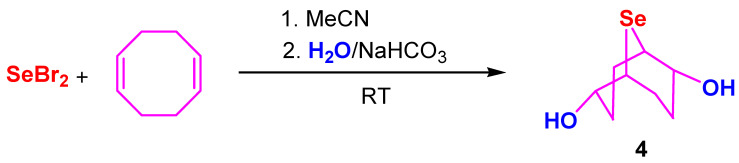
The synthesis of dihydroxy derivative of 9-selenabicyclo[3.3.1]nonane **4**.

A highly efficient synthesis of dihydroxyl derivative of 9-selenabicyclo[3.3.1]nonane **4** in 96% yield was developed through the selenenylation/bis-hydroxylation reaction of selenium dibromide with cis,cis-1,5-cyclooctadiene in acetonitrile in the presence of water and sodium bicarbonate at room temperature (Scheme 2). 

The process was carried out by the addition of selenium dibromide to cis,cis-1,5-cyclooctadiene, followed by the addition of an aqueous solution of sodium bicarbonate to the reaction mixture. The product **4** is an odorless, water-soluble, white crystalline compound that is easy to handle.

**Scheme 3:**
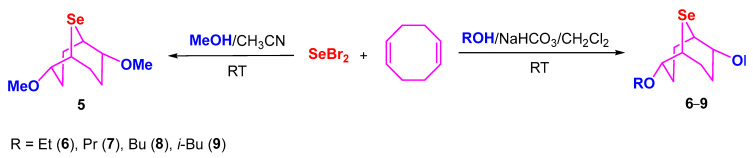
The synthesis of 2,6-dialkoxy-9-selenabicyclo[3.3.1]nonane **5–9**.

The selenenylation/bis-methoxylation reaction proceeded very smoothly in a mixture of acetonitrile and methanol at room temperature. The yield of the product, 2,6-dimethoxy-9-selenabicyclo[3.3.1]nonane **5**, was as high as 98% (Scheme 3).

The process was carried out by adding a solution of selenium dibromide to a mixture of acetonitrile and methanol containing cis,cis-1,5-cyclooctadiene, followed by stirring at room temperature.

**Scheme 4:**
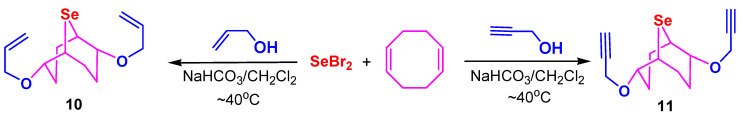
The synthesis of allyloxy and propargyloxy derivatives **10** and **11**.

In the case of other alcohols (ethanol, propanol, butanol and isobutanol), the addition of sodium bicarbonate to the reaction mixture containing alcohols was necessary for effectively conducting the selenenylation/bis-alkoxylation process and obtaining the target products, 2,6-dialkoxy-9-selenabicyclo[3.3.1]nonanes **6–9**, in high yields (91–96%, Scheme 4). 

Unsaturated alcohols, allyl and propargyl alcohols, were successfully involved in the selenenylation/bis-alkoxylation reaction. The nucleophilicity of allyl and propargyl alcohols seems to be somewhat lower than that of the corresponding saturated alcohol, propanol, and also methanol and ethanol. We found that heating (~40 °C) in methylene chloride in the presence of sodium bicarbonate is preferable for obtaining allyloxy and propargyloxy derivatives **10** and **11** in high yields (97% and 95%, respectively) (Scheme 4).

**Scheme 5:**
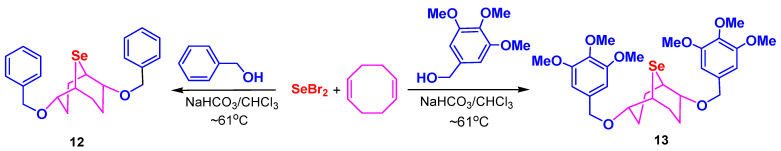
The synthesis of benzyloxy derivatives **12** and **13**.

When using benzyl and 3,4,5-trimethoxybenzyl alcohols under the conditions similar to reactions of alkanols (Scheme 3), as well as to reactions of allyl and propargyl alcohols (Scheme 4), the product yields were not high enough. It was possible to obtain high yields of benzyloxy derivatives by carrying out the reaction in a solvent with a slightly higher boiling point than that of methylene chloride. Refluxing in chloroform in the presence of sodium bicarbonate made it possible to obtain benzyloxy derivatives **12** and **13** in 94% and 92% yields, respectively (Scheme 5).

**Scheme 6:**
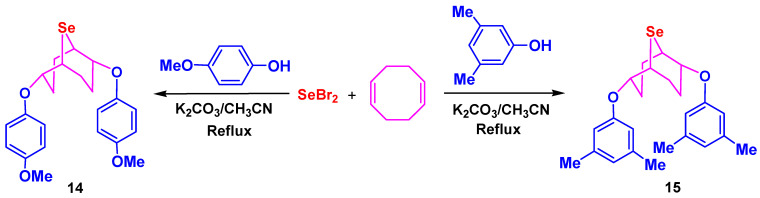
The synthesis of bis(4-methoxyphenoxy) and bis(3,5-dimethylphenoxy) derivatives **14** and **15**.

It is known that phenols are less reactive in nucleophilic reactions than alkanols and usually, in this case, more stringent conditions are required. Indeed, under the conditions of the reactions of alcohols shown in the Scheme 4, phenols gave low yields of the products. The one-pot synthesis of bis(4-methoxyphenoxy) and bis(3,5-dimethylphenoxy) derivatives **14** and **15** in 82–85% yields was developed through the addition of selenium dibromide to cyclooctadiene in acetonitrile, followed by refluxing the reaction mixture in the presence of potassium carbonate (Scheme 6). 

**Scheme 7:**
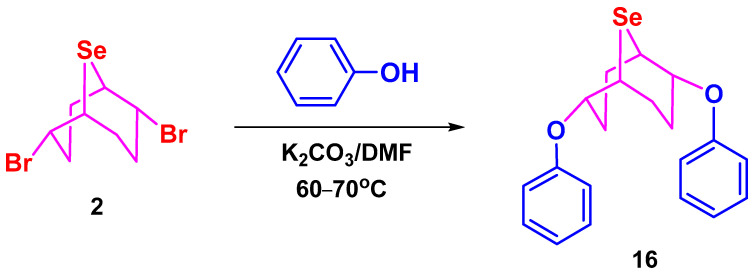
The synthesis of bis(phenoxy) derivative **16** from compound **2** and phenol.

The reaction with unsubstituted phenol under the same conditions, as indicated in Scheme 6, was very sluggish. The use of dibromo derivative **2** as a starting material in the nucleophilic substitution reaction with phenol was chosen as the better approach to the target product. Heating (60–70 °C) compound **2** with phenol in a solution of DMF in the presence of potassium carbonate allowed us to obtain bis-phenolic derivative **16** in 80% yield (Scheme 7). 

It is known that the 1,2,3-triazole derivatives are available by the click chemistry reaction of copper-catalyzed azide-alkynes 1,3-dipolar cycloaddition and this class of organic compounds exhibit a variety of biological activities [62,63,64,65,66,67,68,69,70,71,72,73,74,75,76,77,78,79,80,81,82,83,84,85,86,87,88,89]. However, relatively few cycloaddition reactions with unsubstituted gaseous acetylene under atmospheric pressure have been described in the literature. 

**Scheme 8:**
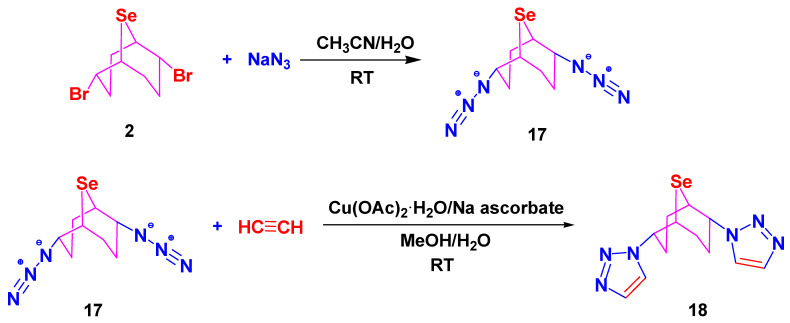
The synthesis of 2,6-bis(1,2,3-triazol-1-yl)-9-selenabicyclo[3.3.1]nonane **18** by the copper-catalyzed azide-alkyne 1,3-dipolar cycloaddition reaction of acetylene with diazido derivative **17**, which was obtained from compound **2** and sodium azide.

We developed the synthesis of the new triazole derivative of selenabicyclo[3.3.1]nonane, 2,6-bis(1,2,3-triazol-1-yl)-9-selenabicyclo[3.3.1]nonane **18**, from diazide **17** and unsubstituted acetylene by the copper-catalyzed azide-alkynes 1,3-dipolar cycloaddition reaction (Scheme 8). 

The catalytic system of Cu(OAc)_2_·H_2_O and sodium ascorbate was used to carry out cycloaddition reactions of diazide **17** with acetylene and propargyl alcohol. This system was found to be very efficient in the reactions of selenium-containing organic azides with terminal acetylenes [90,91]. The active Cu(I) catalyst is generated in situ from the Cu(II) salt via the reduction in copper acetate with sodium ascorbate. The addition of a slight excess of sodium ascorbate prevents the formation of oxidative homocoupling products. 

We involved unsubstituted gaseous acetylene in the copper-catalyzed azide-alkyne 1,3-dipolar cycloaddition reaction with diazido derivative **17** (Scheme 8). The process was carried out under atmospheric pressure by bubbling gaseous acetylene into the reaction mixture. The target product **18** was obtained in 72% yield.

**Scheme 9:**
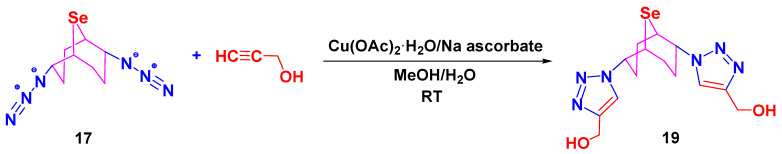
The synthesis of compound **19** by the copper-catalyzed azide-alkyne 1,3-dipolar cycloaddition reaction of diazido derivative **17** with propagyl alcohol.

The efficient synthesis of 2,6-bis(4-hydroxymethyl-1,2,3-triazol-1-yl)-9-selenabicyclo[3.3.1]nonane **19** in 90% yield was developed by the 1,3-dipolar cycloaddition reaction of diazido derivative **17** with propargyl alcohol (Scheme 9).

**Scheme 10:**
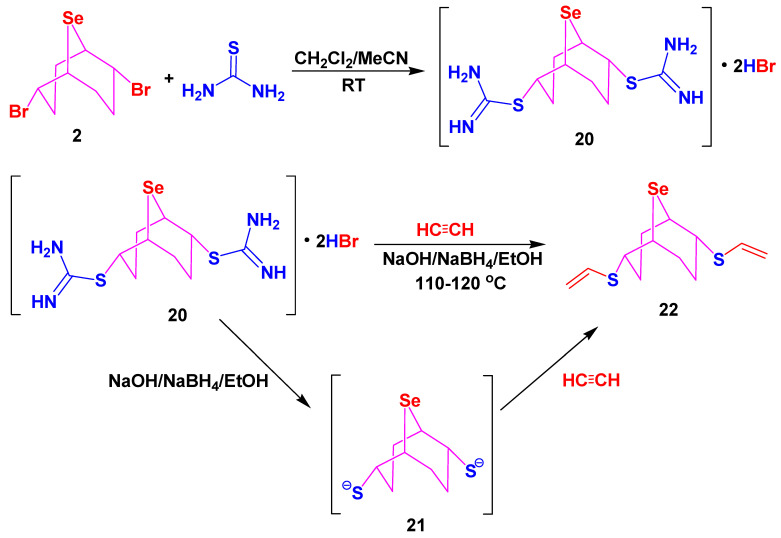
The synthesis of bis(vinylsulfanyl) derivative **22** by the nucleophilic addition of dithiolate anion **21** generated from bis-isothiuronium salt **20**, which was obtained from compound **2** and thiourea.

Along with the 1,3-dipolar cycloaddition reaction, unsubstituted acetylene was involved in the nucleophilic addition reaction of dithiolate anion **21** generated from bis-isothiuronium salt **20**. We previously obtained compound **20** by refluxing dibromo derivative **2** with a high excess of thiourea in acetonitrile [60]. Here, we report the preparation of this compound in 95% yield, at room temperature, at a stoichiometric ratio of the reagents and the synthesis of bis(vinylsulfanyl) derivative of 9-selenabicyclo[3.3.1]nonane **22** in 81% yield (Scheme 10).

**Scheme 11:**
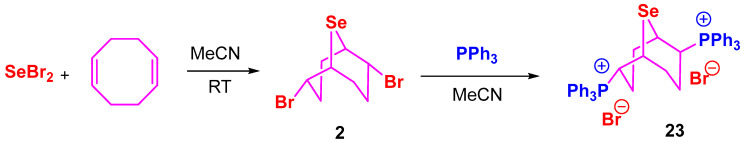
The synthesis of bis-phosphonium salt **23** from compound **2** and triphenyl phosphine.

Thiourea is a sulfur-centered nucleophile. In the same period of the periodic table is phosphorus, the compounds of which are more nucleophilic than their sulfur counterparts. We carried out the reaction of dibromo derivative **2** with triphenyl phosphine, which proceeded smoothly with the formation of water-soluble bis-phosphonium salt **23** in quantitative yield (Scheme 11).

Of the products obtained, compounds **3, 4, 17, 20** and **23** were well water-soluble. These compounds were used for the estimation of glutathione peroxidase-like activity. 

Previously, the glutathione peroxidase-like activity of compound **3** was not estimated. It is important that compound **3** is non-toxic [61] (and probably some other 9-selenabicyclo[3.3.1]nonane derivatives as well). It is also worth noting that 9-selenabicyclo[3.3.1]nonane derivatives have a relatively rigid configuration with a highly sterically accessible selenium atom that can act as an active center for the glutathione peroxidase-like catalysis.

**Scheme 12:**
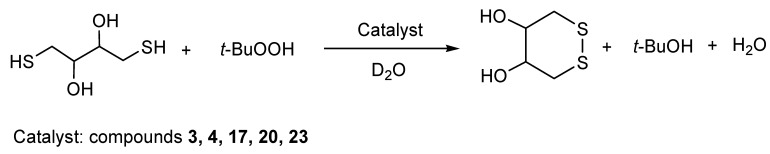
The model reaction of dithiothreitol oxidation by *tert*-butyl hydroperoxide in D_2_O in the presence of synthesized compounds as catalysts (10% mol).

The glutathione peroxidase-like activity of the obtained products was estimated using the model reaction of dithiothreitol oxidation by *tert*-butyl hydroperoxide in D_2_O, in the presence of synthesized compounds as a catalysts (10% mol, Scheme 12) [28,29,30,31,32,33]. The progress of this reaction was monitored by ^1^H NMR spectroscopy at room temperature (*tert*-butyl hydroperoxide, dithiothreitol, 0.025 mmol; tested product, 0.0025 mmol; D_2_O, 0.5 mL). The control experiment was conducted under the same reaction conditions, but in the absence of the catalyst. 

**Figure 2:**
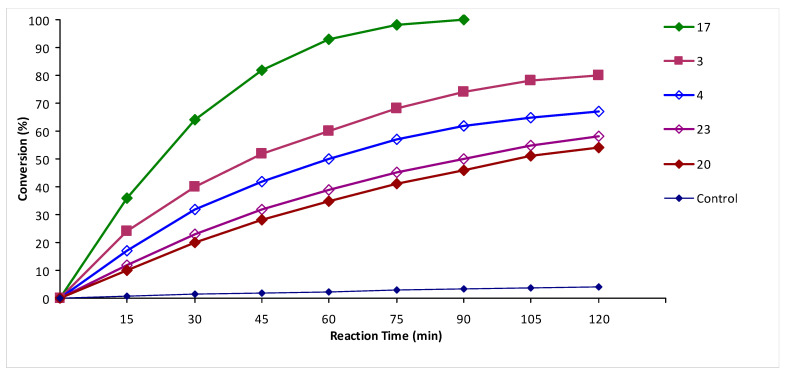
The evaluation of the glutathione peroxidase-like activity of the obtained water-soluble compounds **3, 4, 17, 20** and **23**.

It was found that diazido derivative **17** showed the best activity among the tested products (Figure 2). This compound is considerably superior to other products in activity. The second most active product is bis-pyridinium salt **3**, which is considered a promising drug for metabolic correction during the vaccination process [61]. Compound **4,** with two hydroxyl groups, is the third in activity. Isothiuronium and phosphonium salts **20** and **23** exhibit less activity than compounds **17**, **3** and **4**. 

Compound **17**, which is superior to other products in activity, has two very polar azido groups that have a linear configuration and are located in the relatively rigid molecule on the opposite side of the selenium atom, i.e., the azido groups do not sterically interfere with reactions at the selenium atom and the manifestation of glutathione peroxidase-like activity.

**Scheme 13:**
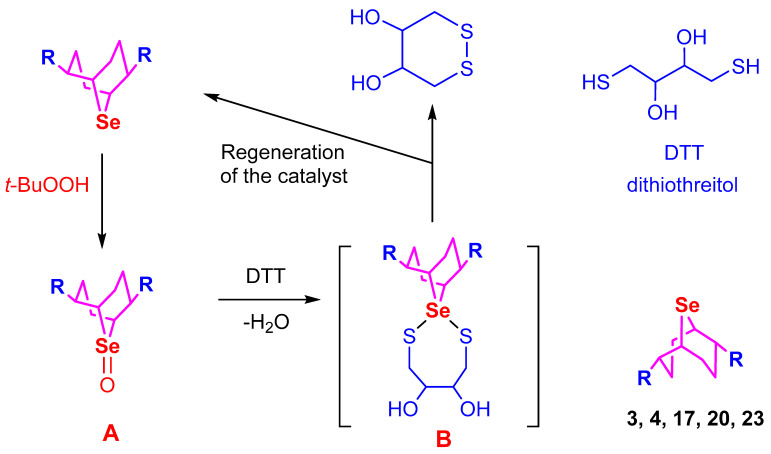
A supposed catalytic cycle with the regeneration of the catalyst.

A supposed catalytic cycle to explain the catalytic effect of the obtained compounds with the regeneration of the catalyst is presented in Scheme 13. The reaction of the catalyst with *tert*-butyl hydroperoxide leads to corresponding selenoxides **A**, which form the heterocyclic intermediate **B** with dithiothreitol. The intermediate **B** undergoes conversion to the oxidized form of dithiothreitol with the regeneration of the catalyst. The intermediates with the sulfur−selenium bond are often considered as intermediates in the oxidation reactions of thiols with peroxides, catalyzed by organoselenium compounds [28,29,30,31,32,33].

The catalytic cycle of glutathione peroxidase in the human body also involves the formation of intermediates with the S−Se bond [92]. Glutathione peroxidase (GPx) contains a selenocysteine residue with the selenol function. The catalytic cycle involves oxidation of the selenol group of the selenocysteine residue by hydrogen peroxide. This process gives selenenic acid derivative (RSeOH), which reacts with glutathione (GSH) to form the intermediate with the S−Se bond (GS−SeR). A second glutathione molecule reduces the GS−SeR intermediate back to the selenol derivative, releasing the disulfide form of glutathione (GS−SG) [92].

Previously, we studied the exchange reactions of dialkyl disulfides with dialkyl diselenides [93]. It was found that the ease of the exchange reaction of dialkyl dichalcogenides (dialkyl disulfides, diselenides and ditellurides) generally rises with the increasing atomic number of the chalcogen and with the decreasing bulk of the alkyl moiety [93]. The equilibrium constants of the exchange reaction of dialkyl disulfides and dialkyl diselenides decrease with the increasing bulk of the alkyl moiety. A number of alkylselenenyl alkyl sulfides have been isolated from the exchange reaction and described for the first time [93].

**Scheme 14:**
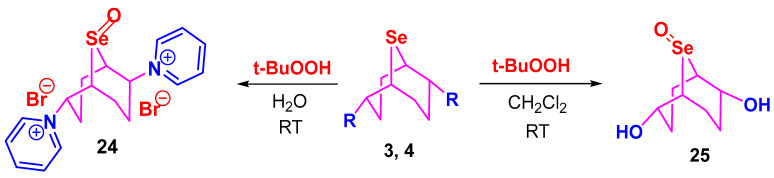
The synthesis of selenoxides **24** and **25** by oxidation of bis-pyridinium salt **3** in water and dihydroxyl derivatives **4** in methylene chloride with *tert*-butyl hydroperoxide.

Selenoxides **A** are considered to be intermediates in the catalytic cycle (Scheme 13). We attempted to obtain corresponding selenoxides from the compounds, which were used for studies of glutathione peroxidase-like activity (Figure 2). It is known that some organic selenoxides are unstable compounds. Pure selenoxides **24** and **25** were obtained in 92–94% yields by oxidation of bis-pyridinium salt **3** in water and dihydroxyl derivatives **4** in methylene chloride with *tert*-butyl hydroperoxide (Scheme 14).

The structural assignments of the synthesized compounds were made using ^1^H, ^13^C, ^77^Se and ^31^P-NMR spectroscopy, including two-dimensional HMBC experiments, and were confirmed by elemental analysis. 

The signals of the carbon atoms of the CH group, which are bonded to the oxygen atom, are observed in the 79–81 ppm region in the ^13^C-NMR spectra of 2,6-diorganyloxy-9-selenabicyclo[3.3.1]nonanes **5**–**16**. The ^13^C-NMR spectra of products **18** and **19** contain signals in the olefin region, which correspond to the C=C group of the triazole ring. 

The obtained values of the ^77^Se-NMR chemical shifts for alkoxy, allyloxy and propargyloxy derivatives **5–13** and aryloxy products **14–16** are very close (~287–293 ppm). The selenium atom in azido compound **17** and the triazole derivatives **18** and **19** resonates at 331.1, 344.7, and 334.2 ppm, respectively. A high downfield shift of the selenium signals is observed for compounds containing positively charged atoms (382.2, 415.1 and 521.4 ppm for bis-isothiuronium **20**, bis-pyridinium **3**, and bis-phosphonium **23** salts, respectively). The obtained values of the ^77^Se-NMR chemical shifts for selenoxides (851.6 and 841.5 ppm for the products **24** and **25**, respectively) are typical for this class of organoselenium compounds.

Characteristic fragment ions [M−R]^+^ in the mass spectra of all products and molecular ions in the mass spectra of organyloxy derivatives **5–11** are observed. The mass spectra of bis-aryloxy derivatives **14–16** show intense ions, which correspond to the elimination of one aryloxy fragment from the molecule.

## 3. Materials and Methods

### 3.1. General Information

The ^1^H (400.1 MHz), ^13^C (100.6 MHz), and ^77^Se (76.3 MHz) NMR spectra (the spectra can be found in the Supplementary Materials) were recorded on a Bruker DPX-400 spectrometer (Bruker BioSpin GmbH, Rheinstetten, Germany) and referred to the residual solvent peaks of CDCl_3_ (δ = 7.27 and 77.16 ppm in ^1^H- and ^13^C-NMR, respectively), DMSO (δ = 2.50 and 39.50 ppm for ^1^H- and ^13^C-NMR, respectively) or D_2_O (δ = 4.79 ppm for ^1^H-NMR) and dimethyl selenide (^77^Se-NMR).

The mass spectra were recorded on a Shimadzu GCMS-QP5050A (Shimadzu Corporation, Kyoto, Japan) with electron impact (EI) ionization (70 eV). The elemental analysis was performed on a Thermo Scientific Flash 2000 Elemental Analyzer (Thermo Fisher Scientific Inc., Milan, Italy). The melting points were determined on a Kofler Hot-Stage Microscope PolyTherm A apparatus (Wagner and Munz GmbH, München, Germany). The distilled organic solvents and degassed water were used in syntheses. 

### 3.2. Synthesis of Compounds ***4–9***

*2,6-Dihydroxy-9-selenabicyclo[3.3.1]nonane* (**4**). A solution of selenium dibromide was prepared from elemental selenium (158 mg, 2 mmol) and bromine (320 mg, 2 mmol) in methylene chloride (1 mL). The solution of selenium dibromide was added dropwise to a solution of cyclooctadiene (216 mg, 2 mmol) in CH_3_CN (10 mL). The mixture was stirred for 4 h at room temperature and a solution of NaHCO_3_ (0.3 g, 3.6 mmol) in water (2 mL) was added. The reaction mixture was stirred overnight (18 h) at room temperature. The solvent was removed on a rotary evaporator, the residue was extracted with methylene chloride (3 × 10 mL). The organic phase was dried over CaCl_2_, the solvent was removed by a rotary evaporator and the residue was dried in vacuum giving the product (424 mg, 96% yield) as white crystals, mp 249–250 °C. 

^1^H NMR (400 MHz, CDCl_3_) δ 1.73–1.81 (m, 2H, CH_2_), 1.85–1.92 (m, 2H, CH_2_), 2.04–2.14 (m, 2H, CH_2_), 2.62–2.70 (m, 4H, CH_2_, OH), 3.59–3.63 (m, 2H, SeCH), 4.29–4.34 (m, 2H, OCH). 

^13^C NMR (100 MHz, CDCl_3_) δ 25.91, 29.50, 30.45, 69.43. 

^77^Se NMR (76.3 MHz, CDCl_3_): 278.1. 

MS (EI): *m/z* (%) = 222 (52, M^+^), 205 (5), 178 (20), 149 (15), 133 (17), 123 (27), 95 (58), 79 (69), 71 (49), 67 (41), 57 (31), 55 (58), 41 (100), 39 (51).

IR (KBr): λ = 873, 982, 1017, 2899, 2932, 3340 cm^−1^. 

Found: C, 43.74; H, 6.56; Se, 35.49. Calc. for C_8_H_14_O_2_Se: C, 43.45; H, 6.38; Se 35.70.

*2,6-Dimethoxy-9-selenabicyclo[3.3.1]nonane* (**5**). A solution of selenium dibromide was prepared from elemental selenium (158 mg, 2 mmol) and bromine (320 mg, 2 mmol) in methylene chloride (1 mL). The solution of selenium dibromide was added dropwise to a solution of cyclooctadiene (216 mg, 2 mmol) in acetonitrile (10 mL). The mixture was stirred for 4 h at room temperature and methanol (2 mL) was added. The mixture was stirred overnight (20 h) at room temperature. The solvent was removed on a rotary evaporator and the residue was dried in vacuum giving the product (488 mg, 98% yield) as a light-yellow oil. 

^1^H NMR (400 MHz, CDCl_3_) δ 1.78–1.82 (m, 2H, CH_2_), 2.00–2.04 (m, 2H, CH_2_), 2.17–2.19 (m, 2H, CH_2_), 2.60–2.64 (m, 2H, CH_2_), 3.02–3.03 (m, 2H, SeCH), 3.36 (s, 6H, CH_3_), 3.88–3.92 (m, 2H, OCH). 

^13^C NMR (100 MHz, CDCl_3_) δ 27.87, 28.05, 28.87, 55.86, 81.04. 

^77^Se NMR (76.3 MHz, CDCl_3_): 288.7 

MS (EI): *m/z* (%) = 250 (30, M^+^), 218 (10), 179 (18), 137 (64), 105 (50), 79 (100), 71 (89), 45 (78), 41 (90).

IR (film): λ = 1086, 1153, 1186, 2817, 2922, 2977 cm^−1^. 

Found: C, 47.89; H, 7.41; Se, 31.43. Calc. for C_10_H_18_O_2_Se: C, 48.20; H, 7.28; Se 31.68.

*2,6-Diethoxy-9-selenabicyclo[3.3.1]nonane* (**6**). A solution of selenium dibromide was prepared from elemental selenium (158 mg, 2 mmol) and bromine (320 mg, 2 mmol) in methylene chloride (1 mL). The solution of selenium dibromide was added dropwise to a solution of cyclooctadiene (216 mg, 2 mmol) in methylene chloride (10 mL). The mixture was stirred for 8 h at room temperature and ethanol (2 mL) and NaHCO_3_ (0.3 g, 3.6 mmol) were added. The mixture was stirred overnight (18 h) at room temperature. The mixture was filtered and the solvent was removed from the filtrate on a rotary evaporator. The residue was dried in vacuum giving the product (532 mg, 96% yield) as a light-yellow oil. 

^1^H NMR (400 MHz, CDCl_3_): 1.20 (t, 6H, CH_3_), 1.80–1.91 (m, 2H, CH_2_), 1.96–2.03 (m, 2H, CH_2_), 2.14–2.24 (m, 2H, CH_2_), 2.64–2.69 (m, 2H, CH_2_), 2.98–3.01 (m, 2H, CHSe), 3.43–3.51 (m, 2H, CH_2_O), 3.57–3.65 (m, 2H, CH_2_O), 3.97–4.03 (m, 2H, CHO). 

^13^C NMR (100 MHz,CDCl_3_): 15.9 (CH_3_), 28.2 (CH_2_), 28.9 (CHSe), 29.5 (CH_2_), 63.6 (CH_2_O), 79.5 (CHO). 

^77^Se NMR (76.3 MHz, CDCl_3_): 288.9. 

MS (EI): *m/z* (%) = 278 (40, M^+^), 232 (14), 193 (30), 151 (36), 123 (35), 105 (50), 85 (48), 79 (60), 57 (100), 41 (86).

IR (film): λ = 1020, 1082, 1159, 2887, 2922, 2971 cm^−1^

Anal. calcd for C_12_H_22_O_2_Se (277.26): C 51.98, H 8.00, O 11.54, Se 28.48%. Found: C 51.89, H 7.96, Se 28.64%.

*2,6-Dipropoxy-9-selenabicyclo[3.3.1]nonane* (**7**) was obtained under the same conditions as compound **6** in 94% yield using propanol.

^1^H NMR (400 MHz, CDCl_3_): 0.86 (t, 6H, CH_3_), 1.48–1.58 (m, 4H, CH_2_), 1.74–1.86 (m, 2H, CH_2_), 1.90–1.97 (m, 2H, CH_2_), 2.07–2.17 (m, 2H, CH_2_), 2.58–2.64 (m, 2H, CH_2_), 2.92–2.95 (m, 2H, CHSe), 3.29–3.38 (m, 2H, CH_2_O), 3.41–3.49 (m, 2H, CH_2_O), 3.89–3.94 (m, 2H, CHO). 

^13^C NMR (100 MHz,CDCl_3_): 10.7 (CH_3_), 23.4 (CH_2_), 28.1 (CH_2_), 28.8 (CHSe), 29.3 (CH_2_), 69.9 (CH_2_O), 79.5 (CHO). 

^77^Se NMR (76.3 MHz, CDCl_3_): 289.2. 

MS (EI): *m/z* (%) = 306 (12, M^+^), 246 (10), 207 (11), 123 (30), 105 (34), 79 (36), 57 (50), 43 (100).

IR (film): λ = 1038, 1082, 1165, 2873, 2932, 2959 cm^−1^

Anal. calcd for C_14_H_26_O_2_Se (305.31): C 55.08, H 8.58, O 10.48, Se 25.86%. Found: C 54.98, H 8.56, Se 26.06%.

*2,6-Dibutoxy-9-selenabicyclo[3.3.1]nonane* (**8**) was obtained under the same conditions as compound **6** in 93% yield using butanol.

^1^H NMR (400 MHz, CDCl_3_): 0.91 (t, 6H, CH_3_), 1.33–1.41 (m, 4H, CH_2_), 1.50–1.57 (m, 4H, CH_2_), 1.81–1.89 (m, 2H, CH_2_), 1.94–2.01 (m, 2H, CH_2_), 2.13–2.21 (m, 2H, CH_2_), 2.62–2.68 (m, 2H, CH_2_), 2.97–2.99 (m, 2H, CHSe), 3.37–3.43 (m, 2H, CH_2_O), 3.50–3.55 (m, 2H, CH_2_O), 3.93–3.98 (m, 2H, CHO). 

^13^C NMR (100 MHz,CDCl_3_): 14.0 (CH_3_), 19.5 (CH_2_), 28.2 (CH_2_), 28.9 (CHSe), 29.4 (CH_2_), 32.4 (CH_2_), 68.1 (CH_2_O), 79.7 (CHO). 

^77^Se NMR (76.3 MHz, CDCl_3_): 288.9. 

MS (EI): *m/z* (%) = 334 (8, M^+^), 260 (5), 221 (8), 165 (13), 123 (32), 105 (27), 79 (41), 57 (92), 41 (100).

IR (film): λ = 1047, 1087, 1148, 2868, 2931, 2966 cm^−1^. 

Anal. calcd for C_16_H_30_O_2_Se (333.37): C 57.65, H 9.07, O 9.60, Se 23.69%. Found: C 57.67, H 9.09, Se 23.85%.

*2,6-Diisobutoxy-9-selenabicyclo[3.3.1]nonane* (**9**) was obtained under the same conditions as compound **6** in 91% yield using isobutanol.

^1^H NMR (400 MHz, CDCl_3_): 0.87–0.93 (m, 12H, CH_3_), 1.77–1.91 (m, 4H, CH_2_, CH), 1.96–2.03 (m, 2H, CH_2_), 2.12–2.22 (m, 2H, CH_2_), 2.64–2.69 (m, 2H, CH_2_), 2.97–3.00 (m, 2H, CHSe), 3.16–3.20 (m, 2H, CH_2_O), 3.26–3.30 (m, 2H, CH_2_O), 3.92–3.97 (m, 2H, CHO). 

^13^C NMR (100 MHz,CDCl_3_): 19.5 (CH_3_), 19.6 (CH_3_), 28.2 (CH_2_), 28.9 (CHSe), 29.0 (CH_2_), 29.4 (CH_2_), 75.4 (CH_2_O), 79.8 (CHO). 

^77^Se NMR (76.3 MHz, CDCl_3_): 286.9. 

MS (EI): *m/z* (%) = 334 (7, M^+^), 260 (6), 221 (5), 165 (14), 123 (29), 105 (23), 79 (37), 57 (91), 41 (100).

IR (film): λ = 1043, 1085, 1139, 2863, 2934, 2959 cm^−1^. 

Anal. calcd for C_16_H_30_O_2_Se (333.37): C 57.65, H 9.07, O 9.60, Se 23.69%. Found: C 57.74, H 9.11, Se 23.72%.

### 3.3. Synthesis of Compounds ***10–13***

*2,6-Diallyloxy-9-selenabicyclo[3.3.1]nonane* (**10**). A solution of selenium dibromide was prepared from elemental selenium (158 mg, 2 mmol) and bromine (320 mg, 2 mmol) in methylene chloride (1 mL). The solution of selenium dibromide was added dropwise to a solution of cyclooctadiene (216 mg, 2 mmol) in a mixture of methylene chloride (10 mL). The mixture was stirred for 4 h at room temperature and allyl alcohol (2 mL) and NaHCO_3_ (0.3 g, 3.6 mmol) were added. The mixture was refluxed for 8 h. The mixture was filtered and the solvent was removed from the filtrate on a rotary evaporator. The residue was dried in vacuum, giving the product (584 mg, 97% yield) as a light-yellow oil. 

^1^H NMR (400 MHz, CDCl_3_): 1.78–1.90 (m, 2H, CH_2_), 1.94–2.00 (m, 2H, CH_2_), 2.11–2.18 (m, 2H, CH_2_), 2.62–2.67 (m, 2H, CH_2_), 2.93–2.96 (m, 2H, CHSe), 3.92–4.06 (m, 6H, CHO, CH_2_O), 5.10–5.25 (dd, 4H, CH_2_=CH), 5.82–5.92 (m, 2H, CH_2_=CH). 

^13^C NMR (100 MHz,CDCl_3_): 28.1 (CH_2_), 28.7 (CHSe), 29.1 (CH_2_), 69.2 (CH_2_O), 79.0 (CHO), 116.6 (CH_2_=CH), 135.3 (CH_2_=CH). 

^77^Se NMR (76.3 MHz, CDCl_3_): 291.6. 

MS (EI): *m/z* (%) = 302 (14, M^+^), 245 (22), 205 (12), 187 (16), 121 (21), 93 (31), 79 (45), 55 (39), 41 (100).

IR (film): λ = 1049, 1069, 1126, 1645, 2850, 2917, 2984 cm^−1^. 

Anal. calcd for C_14_H_22_O_2_Se (301.28): C 55.81, H 7.36, O 10.62, Se 26.21%. Found: C 55.78, H 7.32, Se 26.31%.

*2,6-Dipropargyloxy-9-selenabicyclo[3.3.1]nonane* (**11**) was obtained under the same conditions as compound **10** in 95% yield using propagyl alcohol.

^1^H NMR (400 MHz, CDCl_3_): 1.80–1.91 (m, 2H, CH_2_), 1.99–2.06 (m, 2H, CH_2_), 2.16–2.26 (m, 2H, CH_2_), 2.42 (t, 2H, CCH), 2.65–2.71 (m, 2H, CH_2_), 3.01–3.04 (m, 2H, CHSe), 4.19 (d, 4H, OCH_2_C), 4.21–4.26 (m, 2H, CHO). 

^13^C NMR (100 MHz,CDCl_3_): 28.3 (CH_2_), 28.4 (CH_2_), 29.0 (CHSe), 55.6 (CH_2_O), 74.2 (CCH), 79.0 (CHO), 80.3 (CCH). 

^77^Se NMR (76.3 MHz, CDCl_3_): 295.8. 

MS (EI): *m/z* (%) = 298 (21, M^+^), 259 (6), 243 (10), 203 (11), 161 (18), 133 (21), 107 (22), 91 (41), 79 (37), 55 (42), 39 (100).

IR (film): λ = 1017, 1069, 1160, 2115, 2851, 2918, 2984 cm^−1^. 

Anal. calcd for C_14_H_18_O_2_Se (297.25): C 56.57, H 6.10, O 10.76, Se 26.56%. Found: C 56.63, H 6.06, Se 26.61%.

*2,6-Dibenzyloxy-9-selenabicyclo[3.3.1]nonane* (**12**) was obtained under the same conditions as compound **10** in 94% yield using benzyl alcohol and chloroform.

^1^H NMR (400 MHz, CDCl_3_): 1.94–2.09 (m, 4H, CH_2_), 2.18–2.25 (m, 2H, CH_2_), 2.73–2.79 (m, 2H, CH_2_), 3.05–3.08 (m, 2H, CHSe), 4.11–4.16 (m, 2H, CHO), 4.52–4.57 (m, 2H, CH_2_O), 4.58–4.66 (m, 2H, CH_2_O), 7.28–7.39 (m, 10H, CH_Ar_). 

^13^C NMR (100 MHz,CDCl_3_): 28.3 (CH_2_), 28.8 (CHSe), 29.4 (CH_2_), 70.3 (CH_2_O), 79.3 (CHO), 127.7 (CH_Ar_), 127.7 (CH_Ar_), 128.5 (CH_Ar_), 139.0 (C_Ar_). 

^77^Se NMR (76.3 MHz, CDCl_3_): 291.3. 

MS (EI): *m/z* (%) = 402 (5, M^+^), 203 (10), 105 (8), 91 (100), 79 (45), 65 (12), 41 (7).

IR (film): λ = 1027, 1063, 1087, 1363, 1454, 2860, 2919 cm^−1^.

Anal. calcd for C_22_H_26_O_2_Se (401.40): C 65.83, H 6.53, O 7.97, Se 19.67%. Found: C 65.88, H 6.54, Se 19.72%.

*2,6-Bis(3,4,5-trimethoxybenzyloxy)-9-selenabicyclo[3.3.1]nonane* (**13**) was obtained under the same conditions as compound **10** in 92% yield using 3,4,5-trimethoxybenzyl alcohol and chloroform.

^1^H NMR (400 MHz, CDCl_3_): 1.93–2.09 (m, 4H, CH_2_), 2.17–2.27 (m, 2H, CH_2_), 2.72–2.77 (m, 2H, CH_2_), 3.02–3.05 (m, 2H, CHSe), 3.82 (s, 6H, OCH_3_), 3.84 (s, 12H, OCH_3_), 4.08–4.14 (m, 2H, CHO), 4.42–4.48 (m, 2H, CH_2_O), 4.52–4.58 (m, 2H, CH_2_O), 6.55 (s, 4H, CH_Ar_). 

^13^C NMR (100 MHz,CDCl_3_): 28.3 (CH_2_), 28.7 (CHSe), 29.3 (CH_2_), 56.1 (OCH_3_), 60.8 (OCH_3_), 70.4 (CH_2_O), 79.2 (CHO), 104.6 (CH_Ar_), 134.5 (C_Ar_), 137.5 (C_Ar_), 153.3 (C_Ar_). 

^77^Se NMR (76.3 MHz, CDCl_3_): 292.8. 

MS (EI): *m/z* (%) = 293 (7), 195 (6), 181 (100), 79 (41), 65 (8), 41 (7).

Anal. calcd for C_26_H_38_O_8_Se (557.53): C 56.01, H 6.87, O 22.96, Se 14.16%. Found: C 55.94, H 6.81, Se 14.22%.

### 3.4. Synthesis of Phenol Derivatives ***14–16***

*2,6-Bis(4-methoxyphenoxy)-9-selenabicyclo[3.3.1]nonane* (**14**). The solution of selenium dibromide (1 mmol) was added dropwise to a solution of cyclooctadiene (108 mg, 1 mmol) in acetonitrile (5 mL). The mixture was stirred for 4 h at room temperature and 4-methoxyphenol (310 mg, 2.5 mmol) and powdered potassium carbonate (300 mg, 2.1 mmol) were added and the mixture was refluxed for 16 h. The mixture was cooled, diluted with cold water (20 mL) and extracted with ethyl acetate (3 × 15 mL). The combined organic phase was washed with water, dried over Na_2_SO_4_, and the solvent was removed on a rotary evaporator. The residue was subjected to column chromatography on silica gel (eluent: hexane/chloroform 7:1 → hexane/chloroform 1:5) giving the product (355 mg, 82% yield). 

^1^H NMR (400 MHz, CDCl_3_): 2.17–2.34 (m, 6H, CH_2_), 2.82–2.87 (m, 2H, CH_2_), 3.06–3.10 (m, 2H, CHSe), 3.78 (s, 6H, OCH_3_), 4.87–4.93 (m, 2H, CHO), 6.82–6.86 (s, 4H, CH_Ar_), 6.88–6.93 (m, 4H, CH_Ar_). 

^13^C NMR (100 MHz,CDCl_3_): 28.16(CH_2_), 28.48 (CHSe), 28.99 (CH_2_), 55.82 (OCH_3_), 79.36 (CHO), 114.89 (CH_Ar_), 118.33 (CH_Ar_), 151.20 (OC_Ar_), 154.51 (OC_Ar_). 

^77^Se NMR (76.3 MHz, CDCl_3_): 293.2. 

MS (EI): *m/z* (%) = 311 (79, M^+^–MeC_6_H_4_O), 205 (23), 187 (32), 161 (15), 137 (12), 123 (100), 105 (47), 79 (58), 67 (19), 41 (37). 

IR (film): λ = 1017, 1037, 1103, 1215, 1442, 1453, 1504, 2851, 2918 cm^−1^.

Anal. calcd for C_22_H_26_O_4_Se (433.40): C 60.97, H 6.05, O 14.77, Se 18.22%. Found: C 61.02, H 6.04, Se 18.31%.

*2,6-Bis(3,5-dimethylphenoxy)-9-selenabicyclo[3.3.1]nonane* (**15**) was obtained under the same conditions as compound 14 in 85% yield.

^1^H NMR (400 MHz, CDCl_3_): 2.20–2.26 (m, 4H, CH_2_), 2.28–2.36 (m, 2H, CH_2_), 2.31 (s, 12H, CH_3_), 2.83–2.88 (m, 2H, CH_2_), 3.14–3.16 (m, 2H, CHSe), 5.00–5.05 (m, 2H, CHO), 6.59 (s, 4H, CH_Ar_), 6.64 (s, 2H, CH_Ar_). 

^13^C NMR (100 MHz,CDCl_3_): 21.6 (CH_3_), 28.2 (CH_2_), 28.5 (CHSe), 28.9 (CH_2_), 77.7 (CHO), 114.3 (CH_Ar_), 121.1 (CH_Ar_), 139.5 (C_Ar_), 157.3 (C_Ar_).

^77^Se NMR (76.3 MHz, CDCl_3_): 291.5. 

MS (EI): *m/z* (%) = 309 (97, M^+^–Me_2_C_6_H_4_O), 227 (8), 187 (48), 159 (21), 135 (32), 105 (92), 101 (52), 79 (100), 67 (25), 41 (33). 

IR (film): λ = 1049, 1147, 1187, 1292, 1318, 1472, 1593, 2850, 2919, 2844 cm^−1^.

Anal. calcd for C_24_H_30_O_2_Se (429.45): C 67.12, H 7.04, O 7.45, Se 18.39%. Found: C 67.24, H 7.01, Se 18.55%.

*2,6-Diphenoxy-9-selenabicyclo[3.3.1]nonane* (**16**). Powdered potassium carbonate (300 mg, 2.1 mmol) was added to a mixture of compound **2** (248 mg, 1 mmol), phenol (282 mg, 3 mmol), and DMF (4 mL) and the mixture was heated at 60–70 C for 8 h. The mixture was cooled, diluted with cold water (20 mL) and extracted with ethyl acetate (3 × 15 mL). The combined organic phase was washed with water, dried over Na_2_SO_4_, and the solvent was removed on a rotary evaporator. The residue was subjected to column chromatography on silica gel (eluent: hexane/chloroform 9:1 → hexane/chloroform 1:5) giving the product (299 mg) in 80% yield.

^1^H NMR (400 MHz, CDCl_3_): 2.20–2.35 (m, 6H, CH_2_), 2.82–2.88 (m, 2H, CH_2_), 3.12–3.15 (m, 2H, CHSe), 5.02–5.07 (m, 2H, CHO), 6.93–6.99 (m, 6H, CH_Ar_), 7.30 (t, 4H, CH_Ar_). 

^13^C NMR (100 MHz,CDCl_3_): 28.2 (CH_2_), 28.4 (CHSe), 28.8 (CH_2_), 78.0 (CHO), 116.7 (CH_Ar_), 121.4 (CH_Ar_), 129.8 (CH_Ar_), 157.3 (C_Ar_). 

^77^Se NMR (76.3 MHz, CDCl_3_): 294.8. 

MS (EI): *m/z* (%) = 281 (87, M^+^–C_6_H_5_O), 187 (41), 157 (15), 145 (28), 105 (73), 79 (100), 67 (50), 39 (58). 

IR (film): λ = 1017, 1168, 1226, 1491, 1597, 2853, 2926 cm^−1^.

Anal. calcd for C_20_H_22_O_2_Se (373.35): C 64.34, H 5.94, O 8.57, Se 21.15%. Found: C 64.42, H 5.91, Se 21.20%.

### 3.5. Synthesis of Compounds ***17–19***

*2,6-Diazido-9-selenabicyclo[3.3.1]nonane* (**17**). A solution of sodium azide (1.8 g, 2.7 mmol) in water (14 mL) was added dropwise to a mixture of compound **2** (1 g, 2.87 mmol) and acetonitrile (24 mL) with stirring at room temperature. The reaction mixture was stirred overnight (20 h) at room temperature. Acetonitrile was removed by a rotary evaporator and the residue was extracted with methylene chloride (3 × 20 mL). The organic phase was dried over Na_2_SO_4_, methylene chloride was removed by a rotary evaporator and the residue was dried in vacuum giving a compound **3** (763 mg, 98% yield) as a grey oil. Spectral characteristics and elemental analysis data were reported [49].

^77^Se NMR (76.3 MHz, CDCl_3_): 331.1.

*2,6-Bis(1,2,3-triazol-1-yl)-9-selenabicyclo[3.3.1]nonane* (**18**). A solution of sodium ascorbate (84 mg, 0.42 mmol) in water (2 mL) was added to Cu(OAc)2·H_2_O (42 mg, 0.21 mmol) and the mixture was stirred for 5 min. A solution of compound **3** (189 mg, 0.7 mmol) in methanol (3 mL) was added to the reaction mixture. The reaction mixture was saturated with acetylene by bubbling for 8 h at room temperature with stirring. Then the bubbling of acetylene was stopped, the flask was closed and the mixture was stirred overnight (18 h) at room temperature. The reaction mixture was diluted with H_2_O (8 mL) and extracted with methylene chloride (3 × 10 mL). The organic phase was dried over Na_2_SO_4_, the solvent was removed by a rotary evaporator. The residue was subjected to column chromatography on silica gel (eluent: hexane → hexane/chloroform 1:1 → hexane/chloroform 1:9) giving the product (163 mg) in 72% yield as a white powder; mp 155–157 °C. 

^1^H NMR (400 MHz, CDCl_3_): 2.28–2.36 (m, 2H, CH_2_), 2.46–2.58 (m, 4H, CH_2_), 3.09–3.19 (m, 2H, CH_2_), 3.35–3.40 (m, 2H, CHSe), 5.49–5.55 (m, 2H, CHN), 7.68 (s, 2H, CHCH), 7.77 (s, 2H, CHCH). 

^13^C NMR (100 MHz,CDCl_3_): 27.43(CH_2_), 29.21 (CHSe), 30.36 (CH_2_), 63.04 (CHN), 122.41 (CHCH), 133.60 (CHCH). 

^77^Se NMR (76.3 MHz, CDCl_3_): 344.7. 

MS (EI): *m/z* (%) = 324 (5, M^+^), 255 (50), 186 (44), 145 (12), 105 (100), 79 (61), 67 (38), 41 (67). 

IR (KBr): λ = 1025, 1070, 1113, 1486, 1562, 2851, 2921 cm^−1^.

Anal. calcd for C_12_H_16_N_6_Se (323.26): C 44.59, H 4.99, N 26.00, Se 24.43%. Found: C 44.82, H 5.18, N 25.76, Se 23.15%.

*2,6-Bis(4-hydroxymethyl-1H-1,2,3-triazol-1-yl)-9-selenabicyclo[3.3.1]nonane* (**19**). A solution of sodium ascorbate (84 mg, 0.42 mmol) in water (3 mL) was added to Cu(OAc)_2_^.^H_2_O (42 mg, 0.21 mmol) and the mixture was stirred for 5 min. A solution of compound **2** (189 mg, 0.7 mmol) and propargyl alcohol (136 mg, 2 mmol) in methanol (3 mL) was added dropwise for 10 min. The reaction mixture was stirred for 24 h at room temperature. Methanol was distilled off by a rotary evaporator. The residue was extracted with methylene chloride (3 × 15 mL). The organic phase was dried over Na_2_SO_4_, the solvent and an excess of propargyl alcohol was removed by a rotary evaporator and by drying in vacuum. The product (241 mg, 90% yield) was obtained as a grey powder, mp 181–183 °C.

^1^H NMR (400 MHz, CDCl_3_): 2.14–2.31 (m, 6H, CH_2_), 2.80–2.87 (m, 2H, CH_2_), 3.07–3.11 (m, 2H, CHSe), 3.75 (s, 6H, OCH_3_), 4.86–4.93 (m, 2H, CHO), 6.83–6.92 (m, 8H, CH_Ar_). 

^13^C NMR (100 MHz,CDCl_3_): 26.3 (CH_2_), 28.7 (CHSe), 29.9 (CH_2_), 55.2 (CH_2_OH), 62.2 (CH_2_CHN), 121.6 (CH_Ar_), 147.6 (C_Ar_). 

^77^Se NMR (76.3 MHz, CDCl_3_): 334.2. 

MS (EI): *m/z* (%) = 384 (2, M^+^), 229 (16), 202 (18), 186 (20), 120 (36), 105 (68), 49 (41), 93 (70), 79 (68), 67 (78), 57 (73), 41 (100). 

IR (KBr): λ = 1019, 1046, 1130, 1556, 2863, 2922 cm^−1^.

Anal. calcd for C_14_H_20_N_6_O_2_Se (383.31): C 43.87, H 5.26, N 21.92, O 8.35, Se 20.60%. Found: C 43.74, H 5.24, N 21.98, Se 20.76%.

### 3.6. Synthesis of Compounds ***20–23***

*2,6-Bis[amino(iminio)methylsulfanyl]-9-selenabicyclo[3.3.1]nonane dibromide* (**20**). A solution of compound **2** (0.348 g, 1 mmol) in methylene chloride (5 mL) was added to a mixture of thiourea (0.152 g, 2 mmol) in acetonitrile (5 mL). The mixture was stirred at room temperature overnight (20 h). The formation of white precipitate was observed. Precipitated product was filtered, washed with cold hexane and dried in vacuum, giving bis-isothiouronium salt (0.475 g, 95% yield) as a white powder; mp 219–220 °C. Spectral characteristics and elemental analysis data were reported [50].

^77^Se NMR (76.3 MHz, CDCl_3_): 382.2. 

*2,6-Bis(vinylsulfanyl)-9-selenabicyclo[3.3.1]nonane* (**22**). A solution of sodium hydroxide (80%, 1 g, 20 mmol) and sodium borohydride (0.38 g, 10 mmol) in ethanol (10 mL) was added dropwise to a solution of bis-isothiouronium salt (1 g, 2 mmol) in ethanol (20 mL). The mixture was heated at in a 1 L steel rotating autoclave at temperature 110–120 C for 5 h. Methylene chloride (20 mL) and cold water (120 mL) were added to the reaction mixture. The mixture was transferred to a separatory funnel and the organic layer was separated. The mixture was additionally extracted with methylene chloride (2 × 20 mL), the organic phase was dried over Na_2_SO_4_ and the solvent was removed by a rotary evaporator. The residue was subjected to column chromatography on silica gel (eluent: hexane → hexane/chloroform 15:1 → hexane/chloroform 2:1) giving the product (0.495 g, 81% yield).

^1^H NMR (400 MHz, CDCl_3_): 1.90–2.02 (m, 2H, CH_2_), 2.08–2.15 (m, 2H,CH_2_), 2.26–2.36 (m, 2H, CH_2_), 2.74–2.80 (m, 2H, CH_2_), 3.10–3.12 (m, 2H, CHSe), 3.94–4.00 (m, 2H, CHS), 5.22–5.29 (m, 4H, CH_2_=CHS), 6.30–6.38 (m, 2H, CH_2_=CHS). 

^13^C NMR (100 MHz, CDCl_3_): 29.1 (CH_2_) 29.3 (CHSe), 30.0 (CH_2_), 49.8 (CHS), 114.5 (CH_2_=CH), 131.1 (CH_2_=CH). 

^77^Se NMR (76.3 MHz, CDCl_3_): 285.3. 

Anal. calcd for C_12_H_18_S_2_Se (305.36): C 47.20, H 5.94, S 21.00, Se 25.86%. Found: C 47.32, H 5.99, S 21.12, Se 26.04%.

*2,6-Bis(triphenylphosphonium)-9-selenabicyclo[3.3.1]nonane dibromide* (**23**). A solution of compound **2** (0.348 g, 1 mmol) in methylene chloride (5 mL) was added to

A solution of triphenyl phosphine (0.525 g, 2 mmol) in acetonitrile (5 mL) was added to a mixture of compound **2** (0.348 g, 1 mmol) in acetonitrile (5 mL). The mixture was refluxed for 8 h. The formation of white precipitate was observed. The precipitated product was filtered, washed with cold hexane and dried in vacuum, giving the product (0.837 g, 96% yield) as a white powder; mp 216–218 °C.

^1^H NMR (400 MHz, CDCl_3_): 1.43–1.55 (m, 2H, CH_2_), 1.82–1.91 (m, 4H, CH_2_), 2.60–2.71 (m, 2H, CH_2_), 3.59–3.66 (m, 2H, CHSe), 4.88–4.98 (m, 2H, CHP), 7.57–7.71 (m, 30H, CH_Ar_). 

^13^C NMR (100 MHz,CDCl_3_): 22.9 (CH_2_), 24.8 (CH_2_), 29.6 (CHSe), 29.7 (CHSe), 38.4 (CHP), 38.8 (CHP), 115.7 (C_Ar_), 116.6 (C_Ar_), 130.7 (CH_Ar_), 130.9 (CH_Ar_), 133.8 (CH_Ar_), 133.9 (CH_Ar_), 135.1 (CH_Ar_), 135.2 (CH_Ar_). ^31^P NMR (100 MHz, CDCl_3_): 23.86. 

^77^Se NMR (76.3 MHz, CDCl_3_): 521.4. 

MS (EI): *m/z* (%) = 384 (2, M^+^), 229 (16), 202 (18), 186 (20), 120 (36), 105 (68), 49 (41), 93 (70), 79 (68), 67 (78), 57 (73), 41 (100). 

IR (KBr): λ = 521, 693, 1104, 1436, 1480, 2895, 2992, 3038 cm^−1^.

Anal. calcd for C_44_H_42_P_2_Br_2_Se (895.54): C 61.69, H 4.73, P 6.92, Br 17.84, Se 8.82%. Found: C 61.79, H 4.69, P 6.98, Br 17.99, Se 8.94%.

### 3.7. Synthesis of Selenoxides

*2,6-Dipyridinium-9-selenobicyclo[3.3.1]nonane-9-oxide dibromide* (**24**). A solution of *tert-*butyl hydroperoxide (70%, 2 mmol) was added dropwise to 0.505 g (1 mmol) 2,6-dipyridinium 9-selenobicyclo[3.3.1]nonane dibromide **3**. The reaction mixture is stirred for 2 h at room temperature. The reaction mixture was washed with acetonitrile (10 mL), the precipitate was filtered off and dried under vacuum. The product was isolated as a light-yellow powder (0.49 g, 94% yield), mp 96–98 °C (decomp.).

^1^H NMR (400 MHz, CDCl_3_) δ 2.41–2.59 (m, 4H, OCHCH_2_, SeCHCH_2_), 2.66–2.79 (m, 2H, OCHCH_2_, SeCHCH_2_), 3.27–3.46 (m, 2H, SeCHCH_2_), 3.95–4.01 (m, 2H, SeCH), 5.69–5.76 (m, 2H, NCHCH_2_), 8.27–8.32 (m, 4H, CH_Ar_), 8.72–8.78 (m, 2H, CH_Ar_), 9.18–9.23 (m, 4H, CH_Ar_). 

^13^C NMR (100 MHz, CDCl_3_) δ 18.75, 22.02, 25.46, 26.15, 47.41, 49.36, 66.94, 70.57, 130.17, 144.56, 144.94, 148.16, 148.35. 

^77^Se NMR (76.3 MHz, CDCl_3_): 851.6. 

Found: C, 41.27; H, 4.36; N, 5.66; Br, 31.03; Se, 15.44. Calc. for C_18_H_22_N_2_Br_2_OSe: C, 41.48; H, 4.25; N, 5.38; Br, 30.66; O, 3.07; Se 15.15.

*2,6-Dihydroxy-9-selenobicyclo[3.3.1]nonane-9-oxide* (**25**). A solution (0.25 mL) of *tert*-butyl peroxide (70%, 2 mmol) was added dropwise to a solution of 0.221 g (1 mmol) of 2,6-hydroxy-9-selenobicyclo[3.3.1]nonane **4** in methylene chloride (10 mL). The reaction mixture was stirred for 12 h at 0 °C. The mixture was washed with water (5 × 10 mL), dry with CaCl_2_, and the solvent was removed on a rotary evaporator. The residue was dried in vacuum. The product was isolated as a white powder (0.218 g, 92% yield), mp 86–88 °C (decomp.). 

^1^H NMR (400 MHz, CDCl_3_) δ 1.56–1.64 (m, 1H, SeCHCH_2_), 1.72–1.87 (m, 4H, OCHCH_2_, SeCHCH_2_) 2.14–2.17 (m, 2H, SeCHCH_2_), 2.31–2.37 (m, 1H, OCHCH_2_), 2.96–2.98 (m, 1H, SeCH), 2.99–3.01 (m, 1H, SeCH), 3.92 (s, 1H, OH), 4.22 (s, 1H, OH), 4.89–4.91 (m, 1H, OCH), 5.24–5.26 (m, 1H, OCH). 

^13^C NMR (100 MHz, CDCl_3_) δ 16.53, 19.23, 29.56, 30.09, 46.66, 50.15, 62.44, 67.92. 

^77^Se NMR (76.3 MHz, CDCl_3_): 841.5. 

Found: C, 40.64; H, 6.01; Se, 32.79. Calc. for C_8_H_12_O_3_Se: C, 40.52; H, 5.95; O, 20.24; Se 33.30.

## 4. Conclusions

A set of highly efficient syntheses of novel derivatives of 9-selenabicyclo[3.3.1]nonane in high yields based on selenium dibromide and cis,cis-1,5-cyclooctadiene were developed. Various oxygen-centered nucleophiles were involved in the selenenylation/bis-oxylation reactions including alkanols, benzyl, allyl, and propargyl alcohols, and phenols. The copper-catalyzed 1,3-dipolar cycloaddition of 2,6-diazido-9-selenabicyclo[3.3.1]nonane with unsubstituted gaseous acetylene and propargyl alcohol was used for the preparation of novel 1,2,3-triazole derivatives of selenabicyclo[3.3.1]nonane. 

Bis-isothiuronium salt was obtained in 95% yield, at room temperature, at a stoichiometric ratio of compound **2** and thiourea. This salt was used for the generation of the corresponding dithiolate anion under the action of sodium hydroxide, followed by the nucleophilic addition of the dithiolate anion to unsubstituted acetylene with the formation of bis(vinylsulfanyl) derivative of 9-selenabicyclo[3.3.1]nonane. The synthesis of water-soluble bis-phosphonium salt in quantitative yield was developed from dibromo derivative **2** and triphenyl phosphine.

The obtained water-soluble products were used for the estimation of glutathione peroxidase-like activity. It was found that diazido derivative **17** is considerably superior to other products in activity. The second most active product is bis-pyridinium salt **3**, which is considered a promising drug for metabolic correction during vaccination process [61]. The selenoxides **24** and **25**, which are supposed to be the catalytic cycle intermediates, were synthesized by the oxidation of compounds **3** and **4** with *tert*-butyl hydroperoxide.

## Data Availability

Not applicable.

## Supplementary Materials

The following supporting information can be downloaded at: https://www.mdpi.com/article/10.3390/ijms232415629/s1.
