# Supplementary material for: Click Chemistry of Selenium Dihalides: Novel Bicyclic Organoselenium Compounds Based on Selenenylation/Bis-Functionalization Reactions and Evaluation of Glutathione Peroxidase-like Activity
Source: Int J Mol Sci. 2022 Dec 9;23(24):15629. doi: 10.3390/ijms232415629 (PMC9779772; doi:10.3390/ijms232415629)

Supplementary Materials

Click Chemistry of Selenium Dihalides: Novel Bicyclic Organoselenium Compounds Based on Selenenylation/Bis-Functionalization Reactions and Evaluation of Glutathione Peroxidase-like Activity

Maxim V. Musalov * and Vladimir A. Potapov

A. E. Favorsky Irkutsk Institute of Chemistry, Siberian Division of The Russian Academy of Sciences, 1 Favorsky Str., 664033 Irkutsk, Russia

* Correspondence: musalov_maxim@irioch.irk.ru

Table of Contents

Experimental (General Information)	2
Examples of ^{1}H and ^{13}C-NMR Spectra	3-11
Examples of ^{1}H and ^{13}C-NMR Spectra	11-22
Examples of ^{77}Se-NMR and ^{1}H-^{77}Se-HMBC NMR Spectra	22-25
Examples of Mass Spectra	26-29

Experimental (General Information)

The ^{1}H (400.1 MHz), ^{13}C (100.6 MHz), and ^{77}Se (76.3 MHz) NMR spectra were recorded on a Bruker DPX-400 spectrometer (Bruker BioSpin GmbH, Rheinstetten, Germany) and referred to the residual solvent peaks of $CDCl_3$ (δ = 7.27 and 77.16 ppm in ^{1}H- and ^{13}C-NMR, respectively), DMSO (δ = 2.50 and 39.50 ppm for ^{1}H- and ^{13}C-NMR, respectively) or D_2O (δ = 4.79 ppm for ^{1}H-NMR) and dimethyl selenide (^{77}Se-NMR).

The mass spectra were recorded on a Shimadzu GCMS-QP5050A (Shimadzu Corporation, Kyoto, Japan) with electron impact (EI) ionization (70 eV).

Examples of ^{1}H-NMR Spectra

^{1}H-NMR spectrum of compound 3 (D_2O)

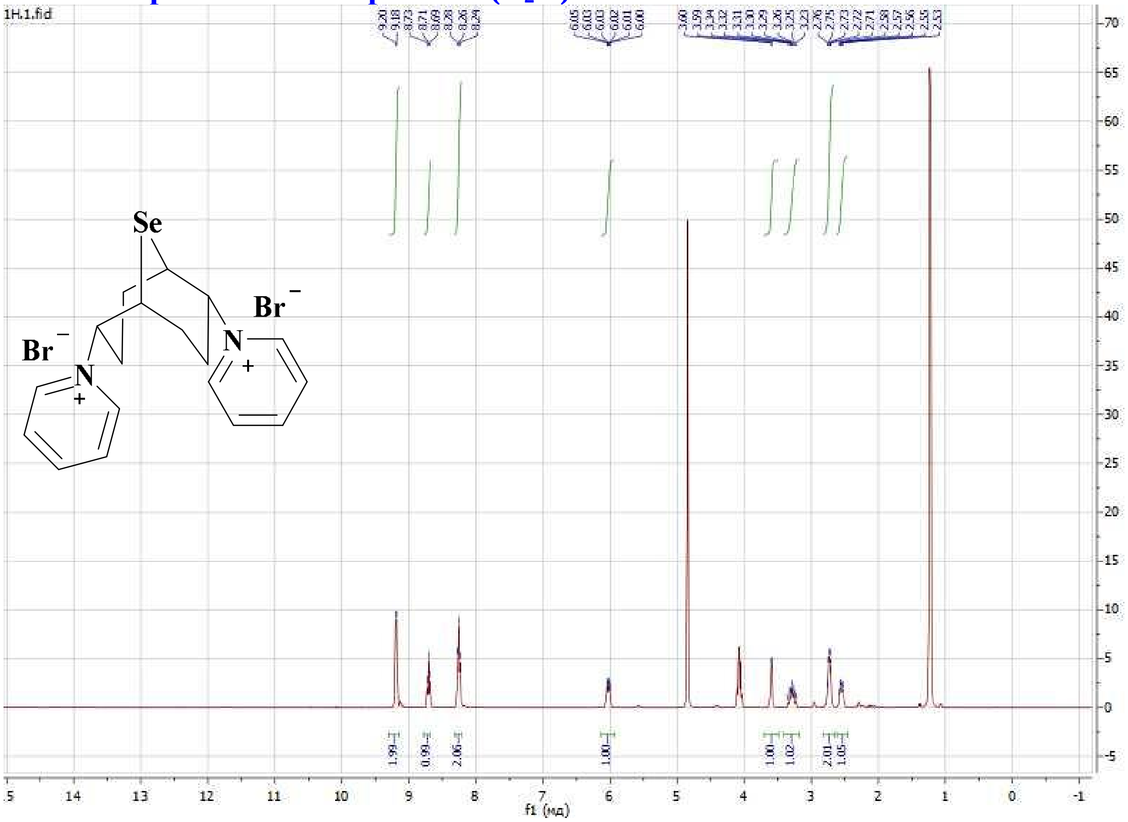

^{1}H-NMR spectrum of compound 4

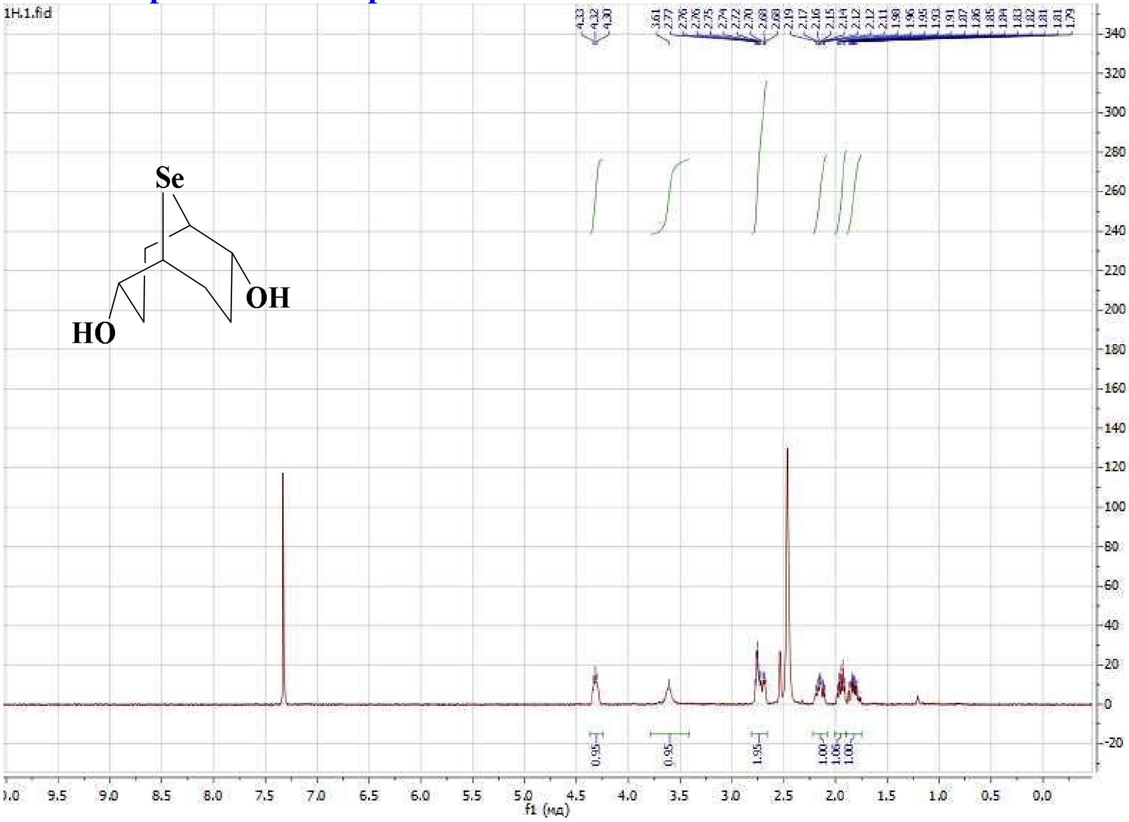

^{1}H-NMR spectrum of compound 5

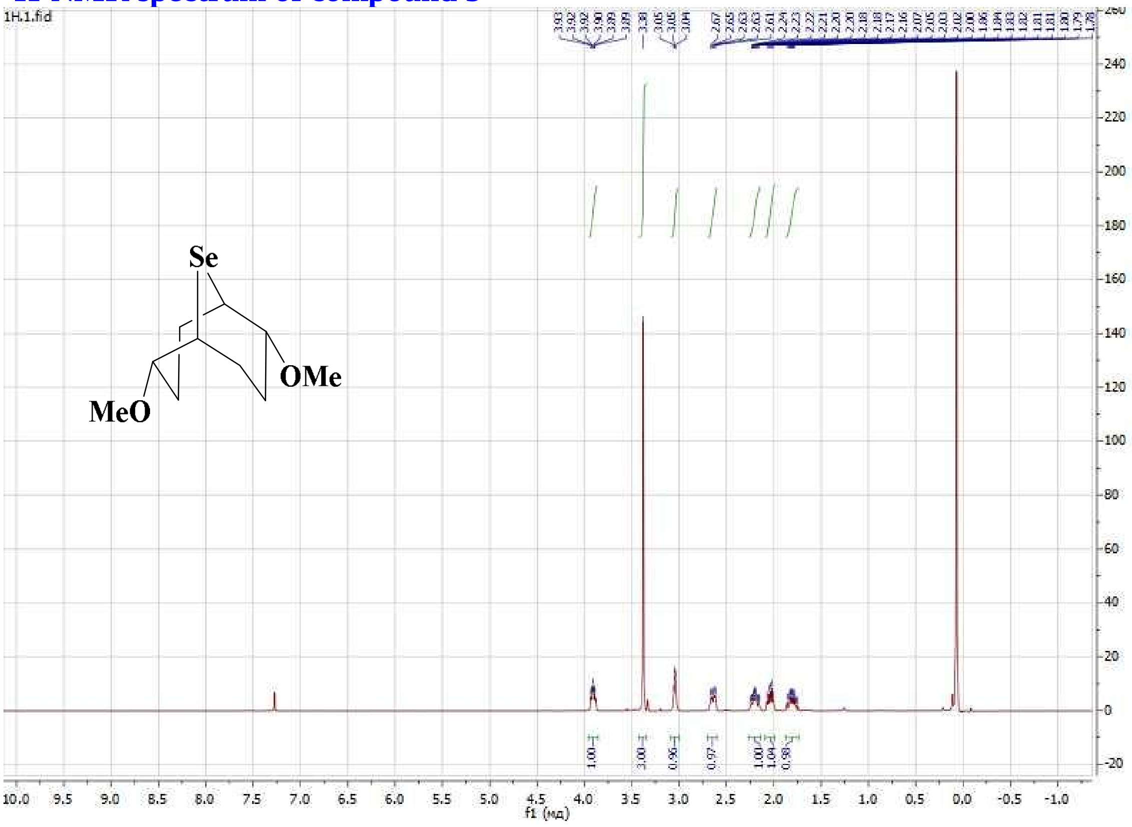

^{1}H-NMR spectrum of compound 6

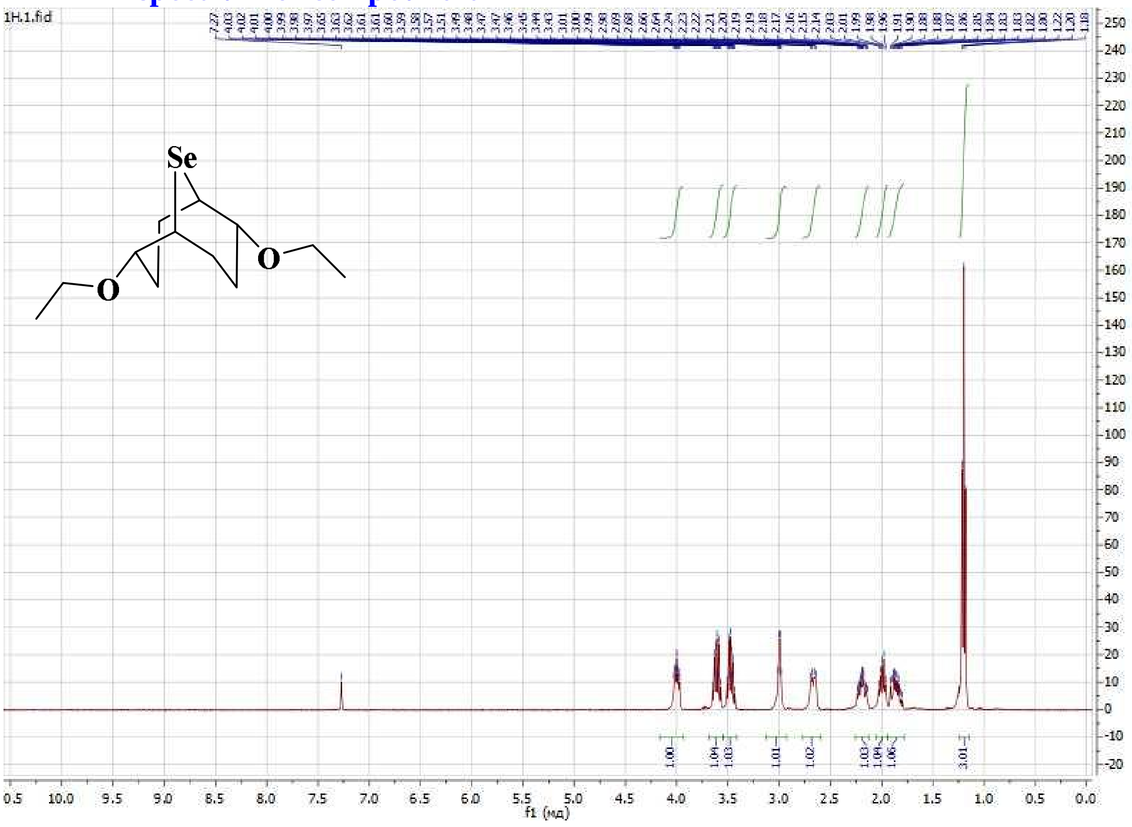

^{1}H-NMR spectrum of compound 7

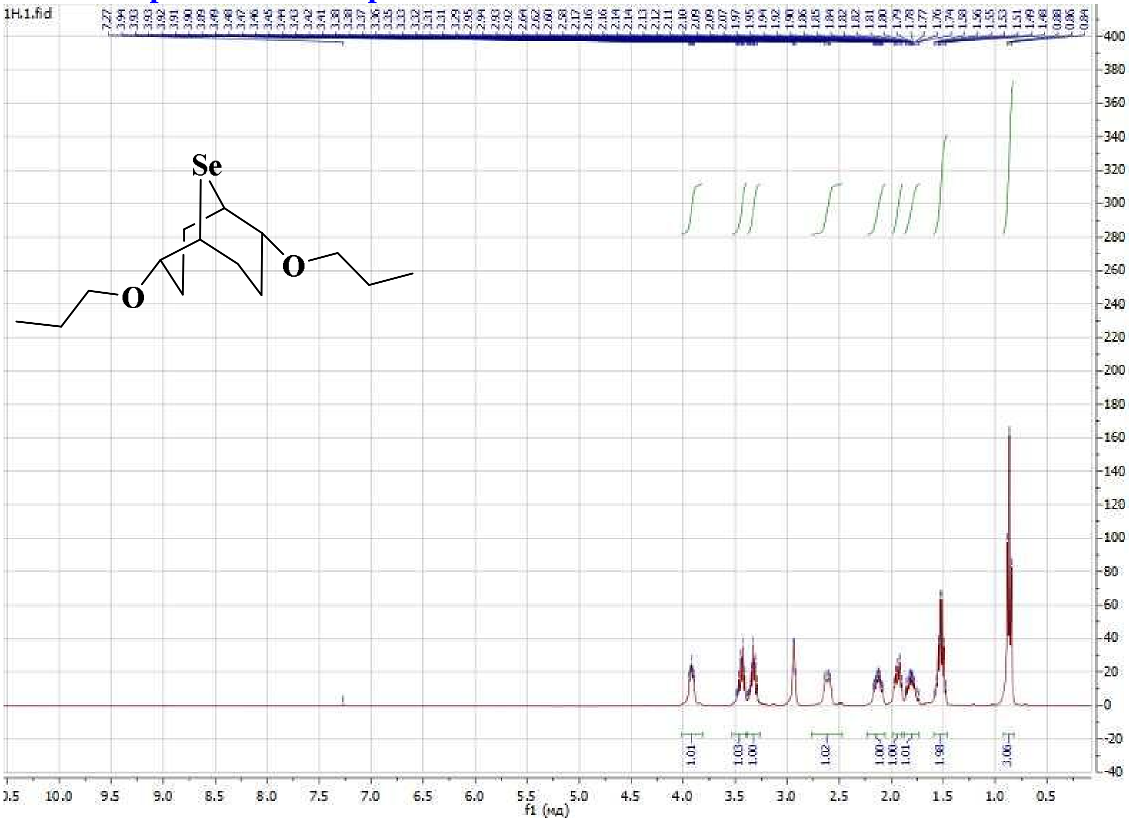

^{1}H-NMR spectrum of compound 8

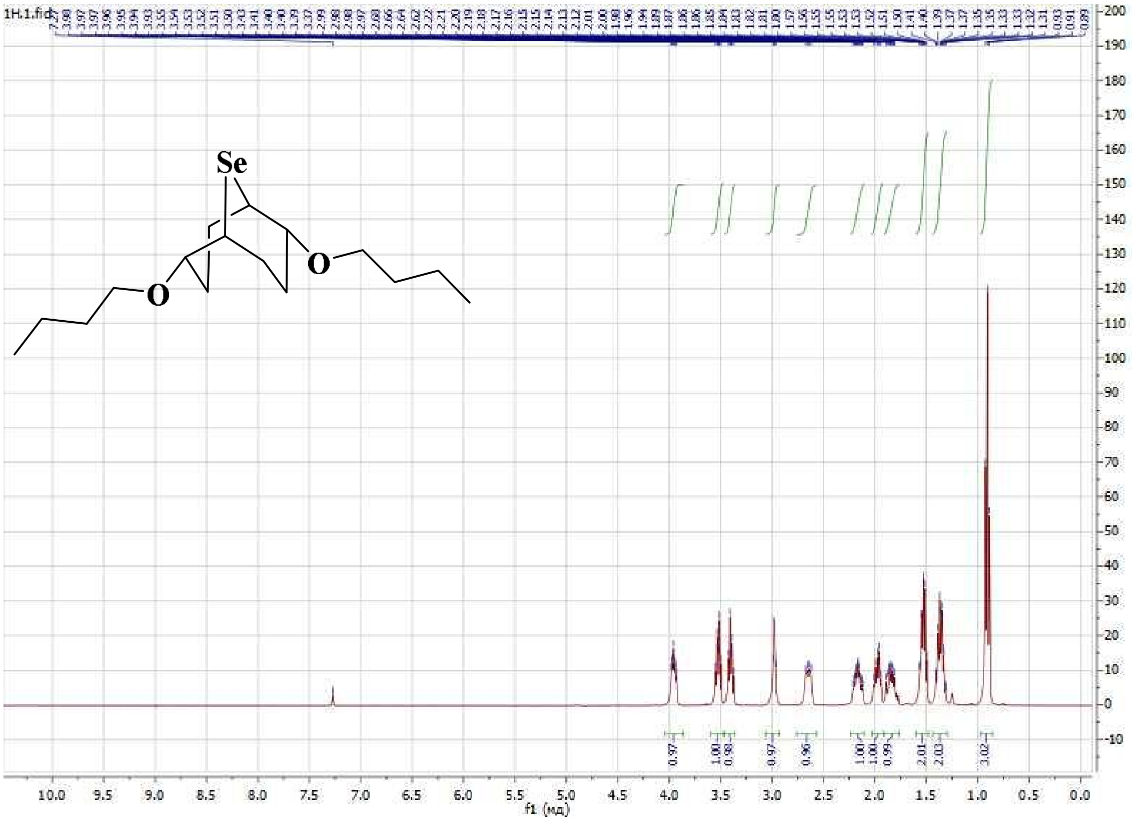

^{1}H-NMR spectrum of compound 9

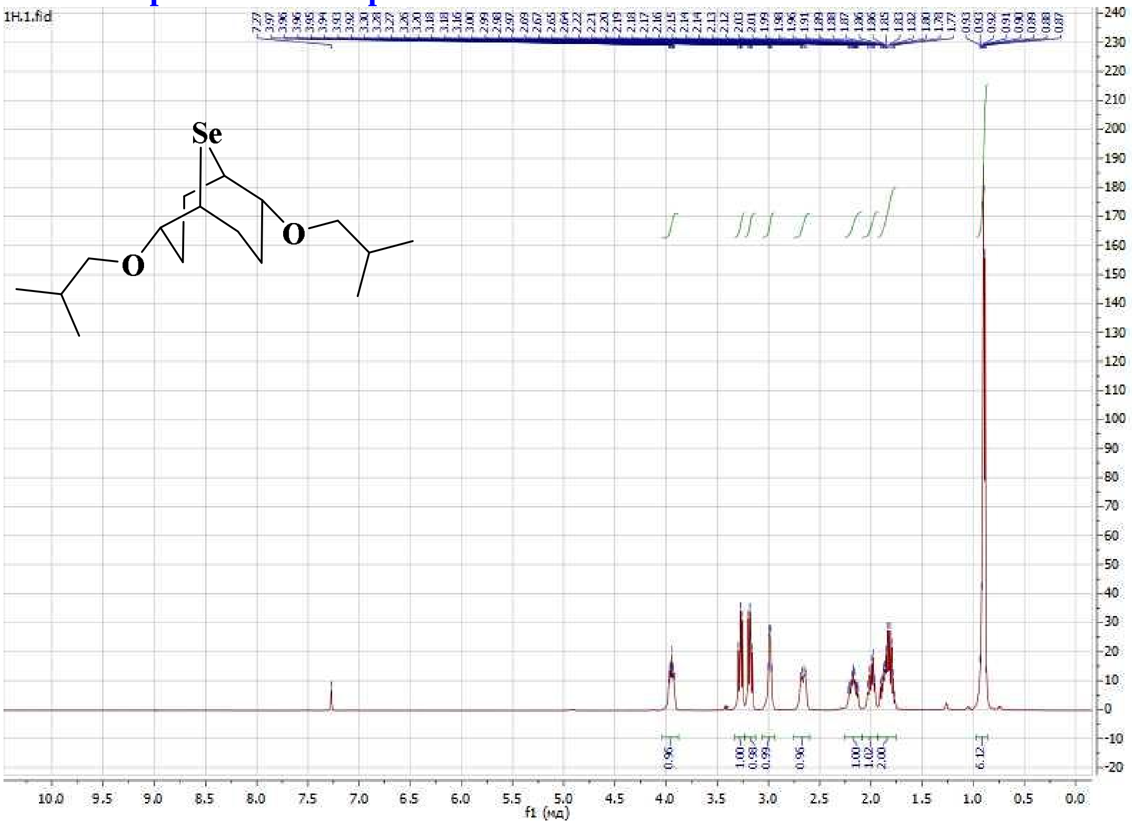

^{1}H-NMR spectrum of compound 10

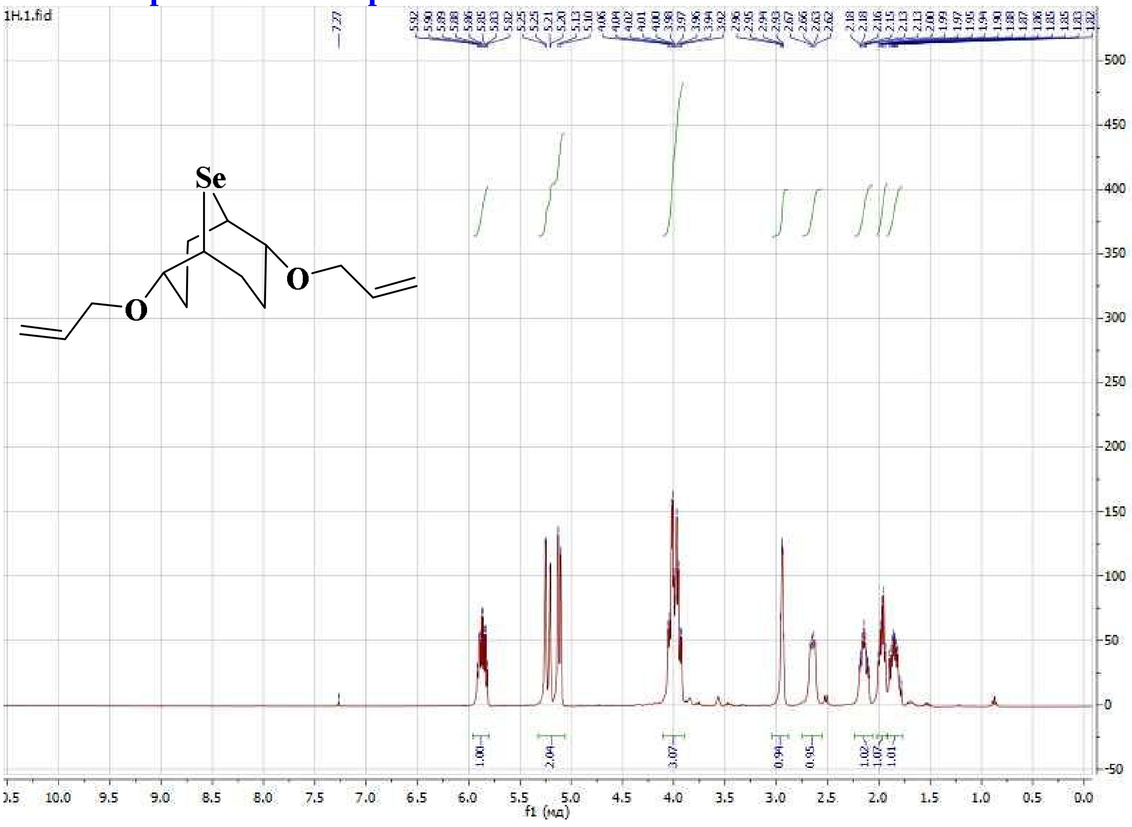

^{1}H-NMR spectrum of compound 11

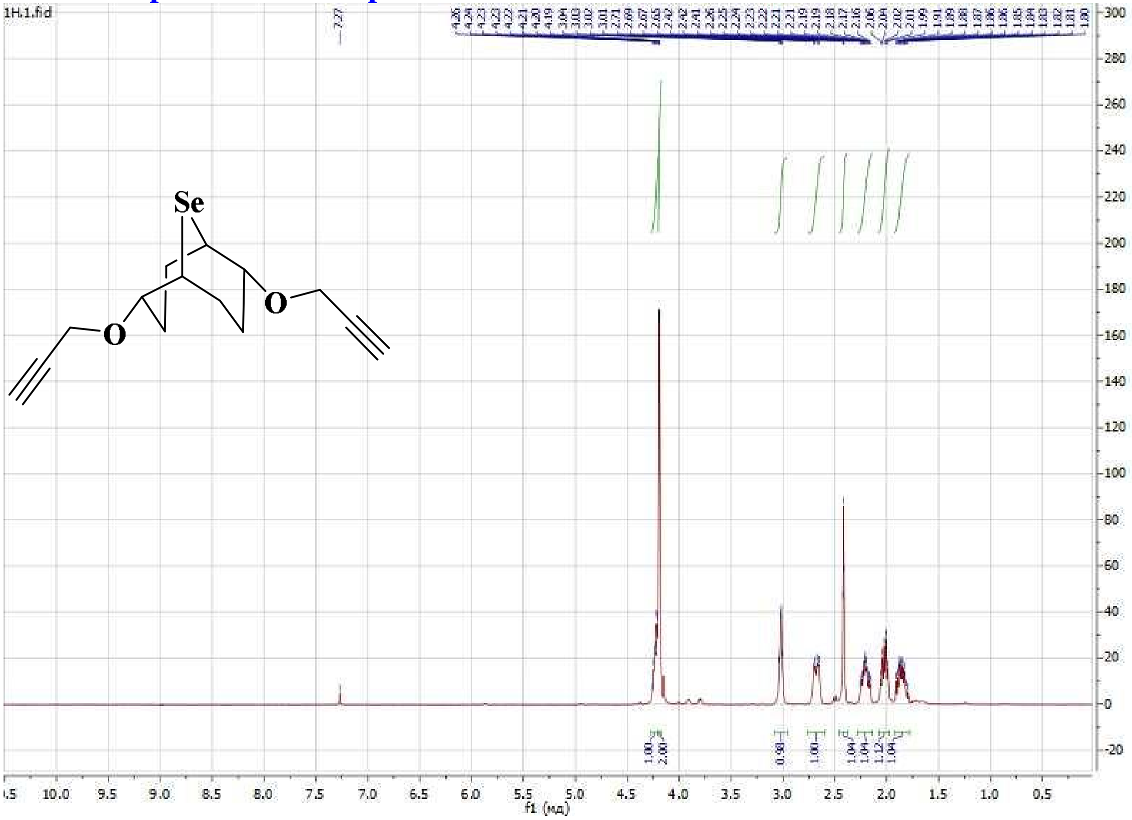

^{1}H-NMR spectrum of compound 12

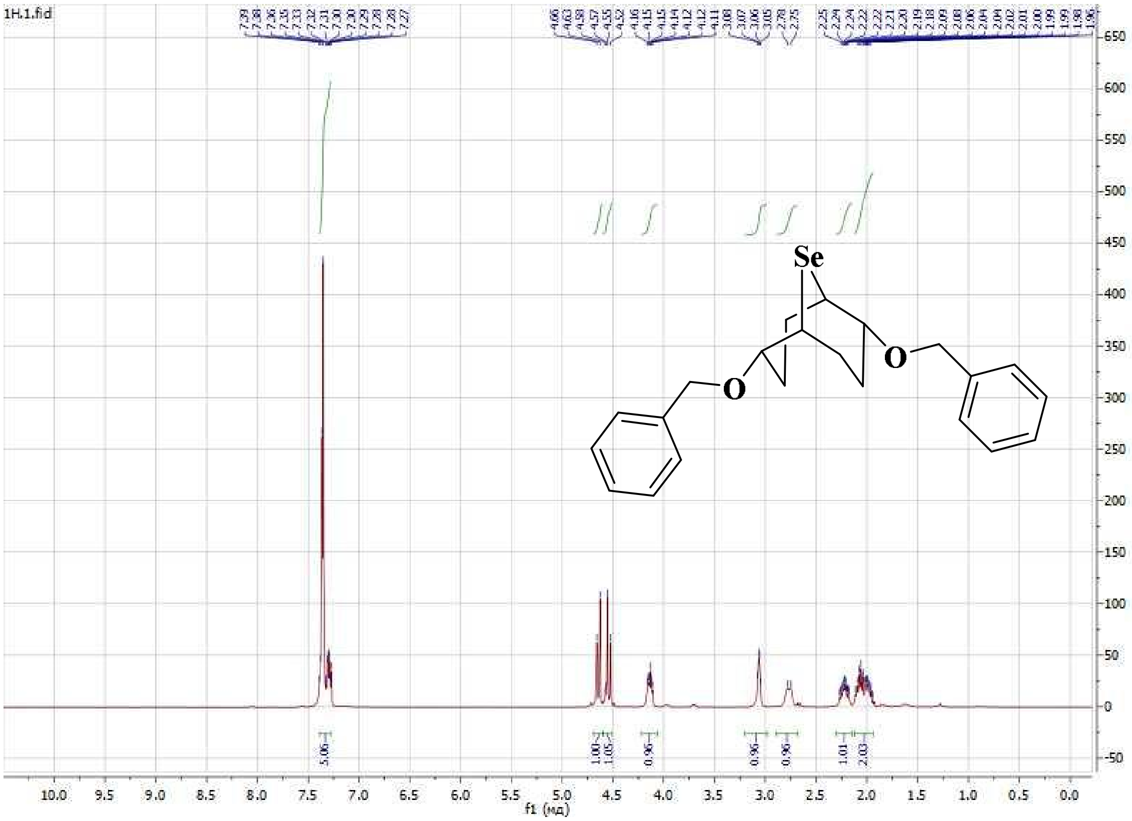

^{1}H-NMR spectrum of compound 13

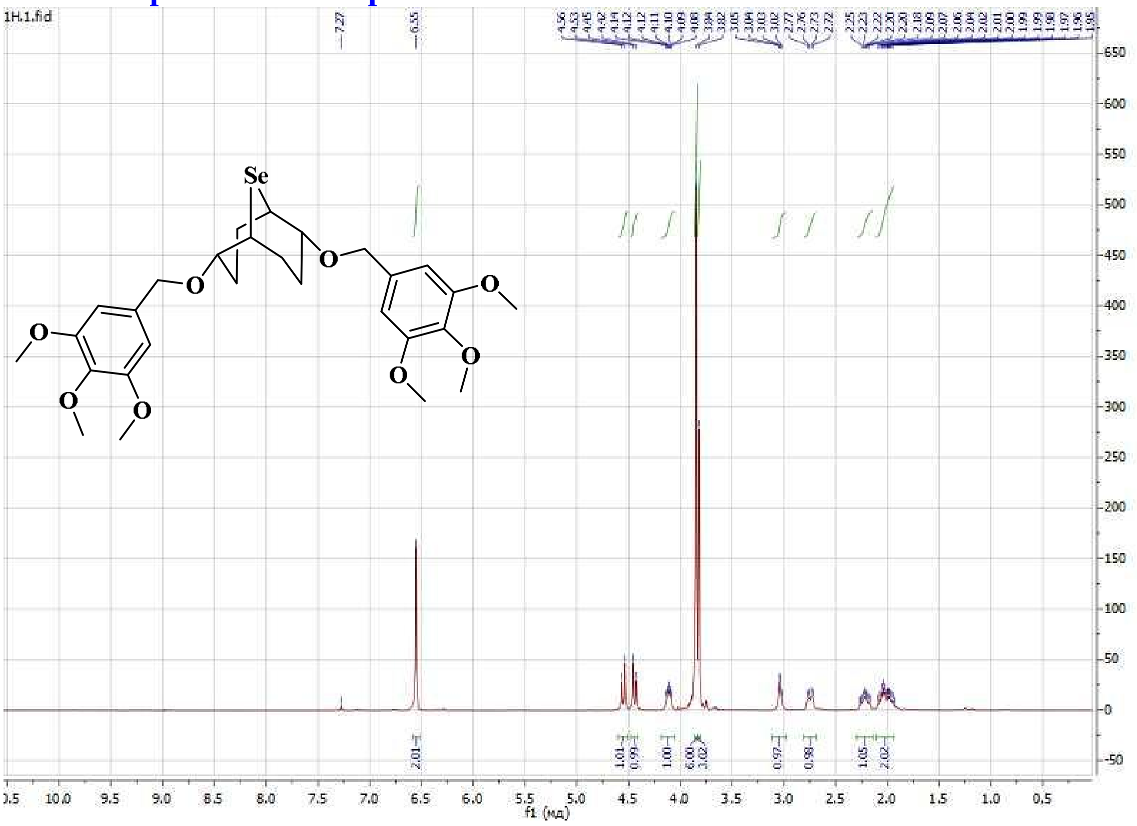

^{1}H-NMR spectrum of compound 14

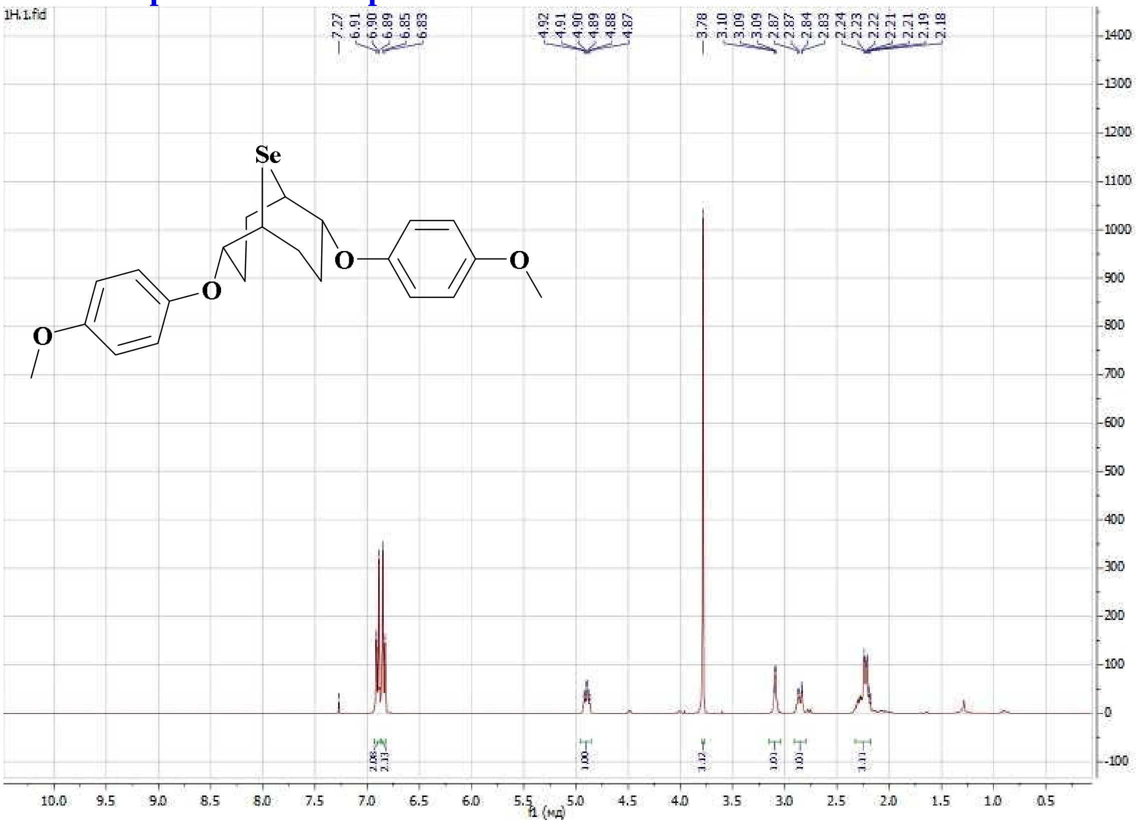

^{1}H-NMR spectrum of compound 15

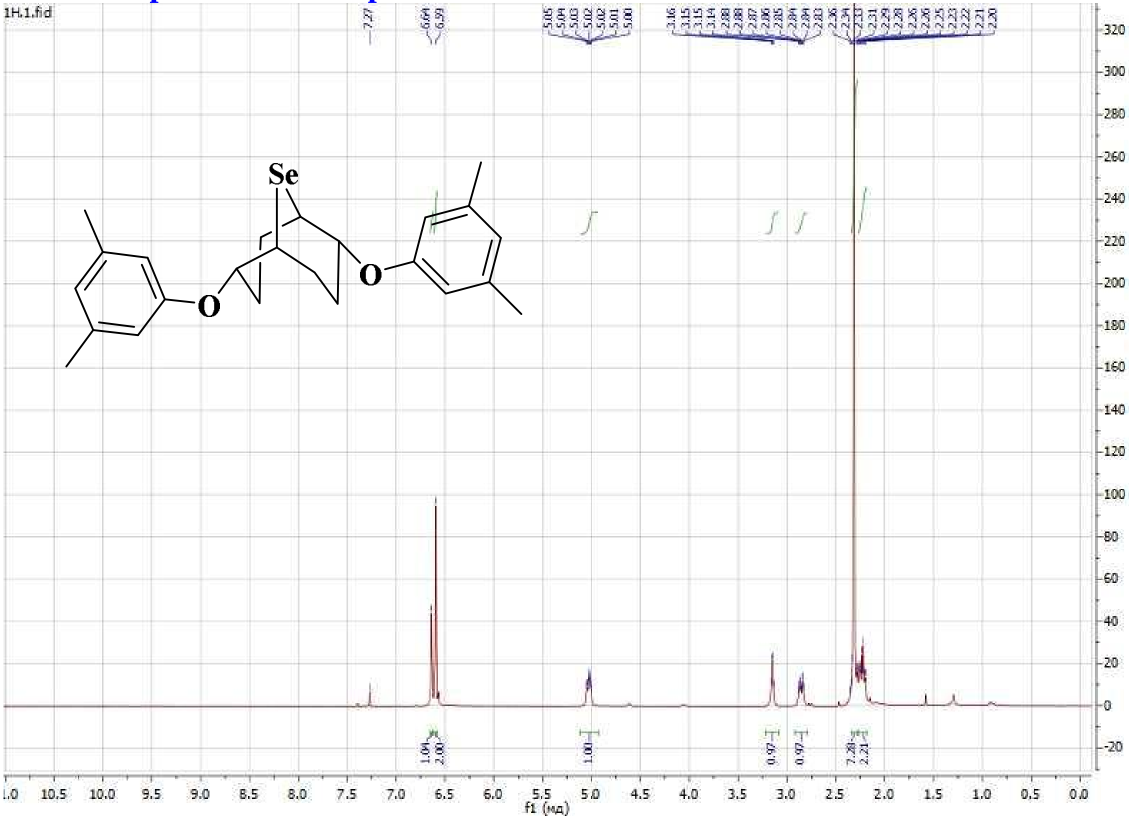

^{1}H-NMR spectrum of compound 16

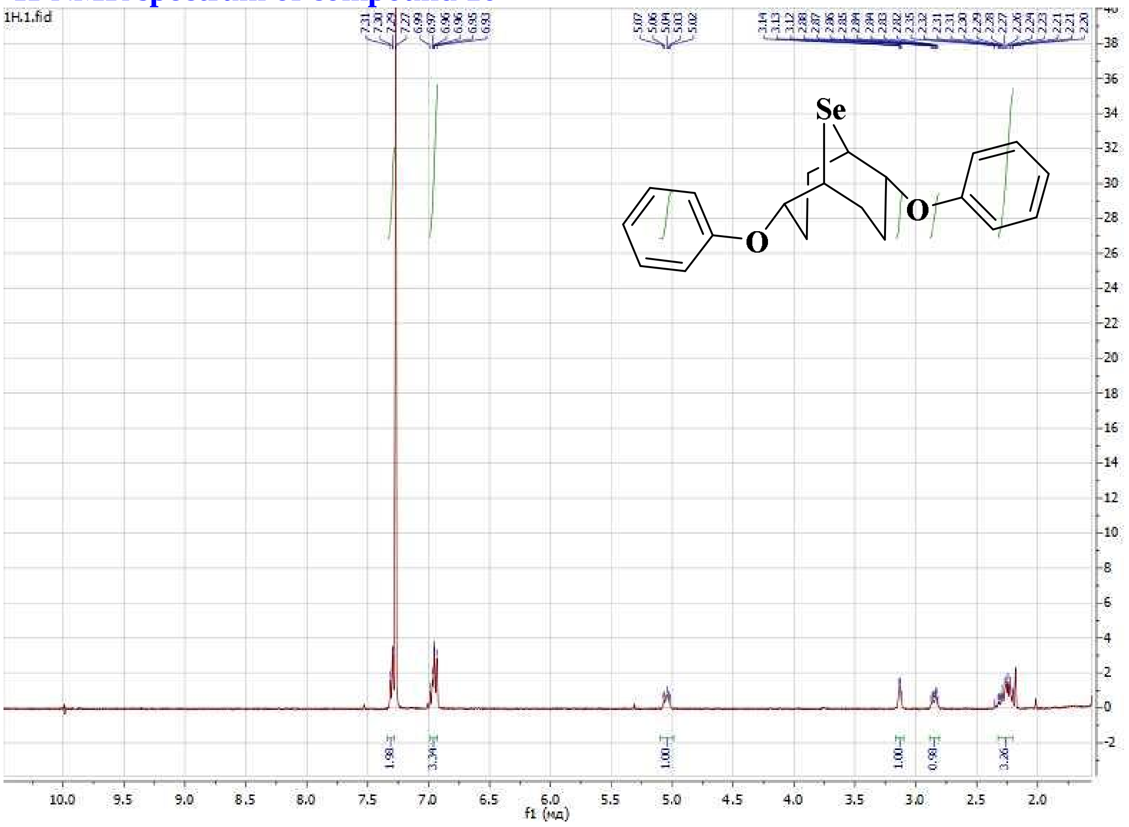

^{1}H-NMR spectrum of compound 18

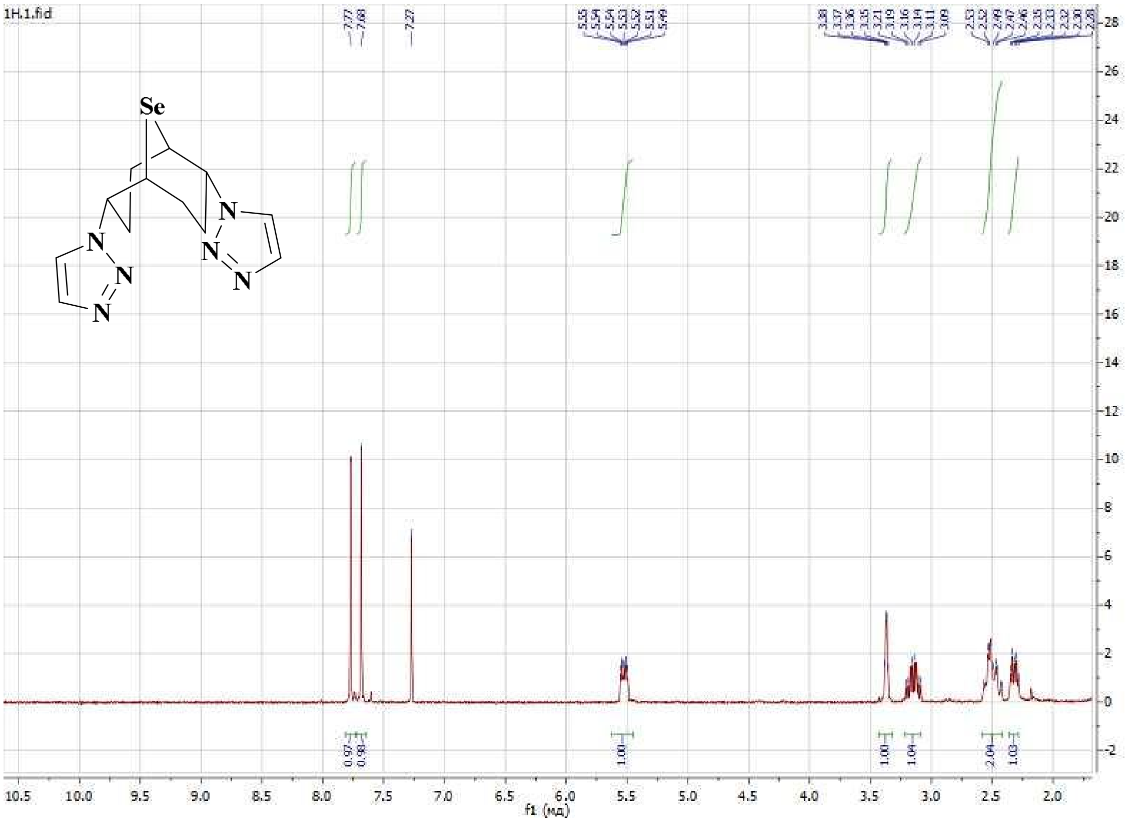

^{1}H-NMR spectrum of compound 22

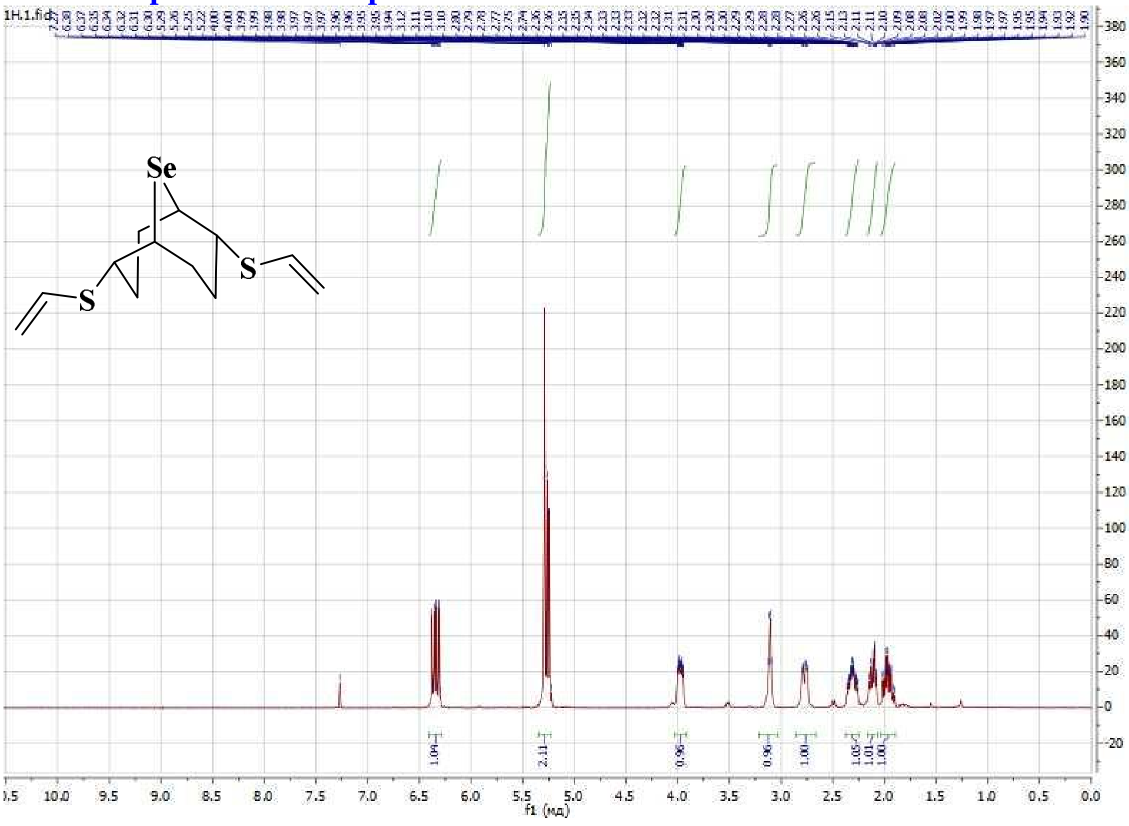

^{1}H-NMR spectrum of compound 23

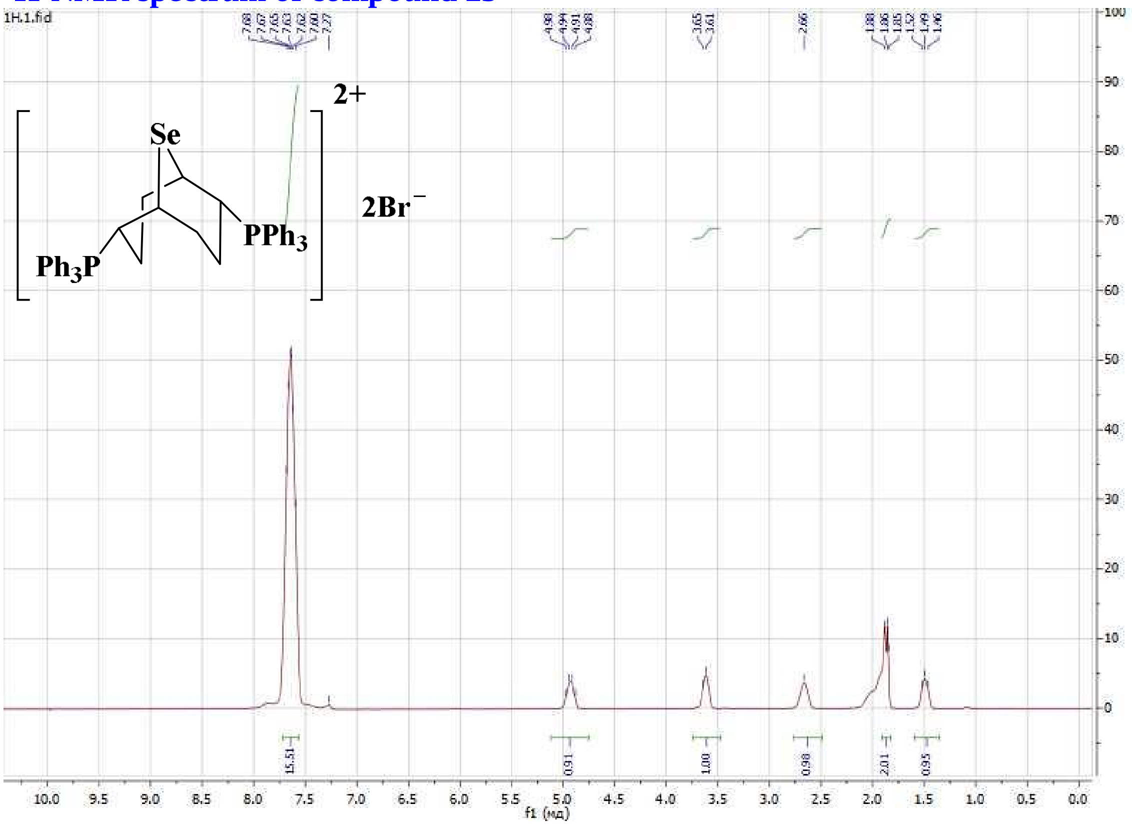

^{1}H-NMR spectrum of compound 24

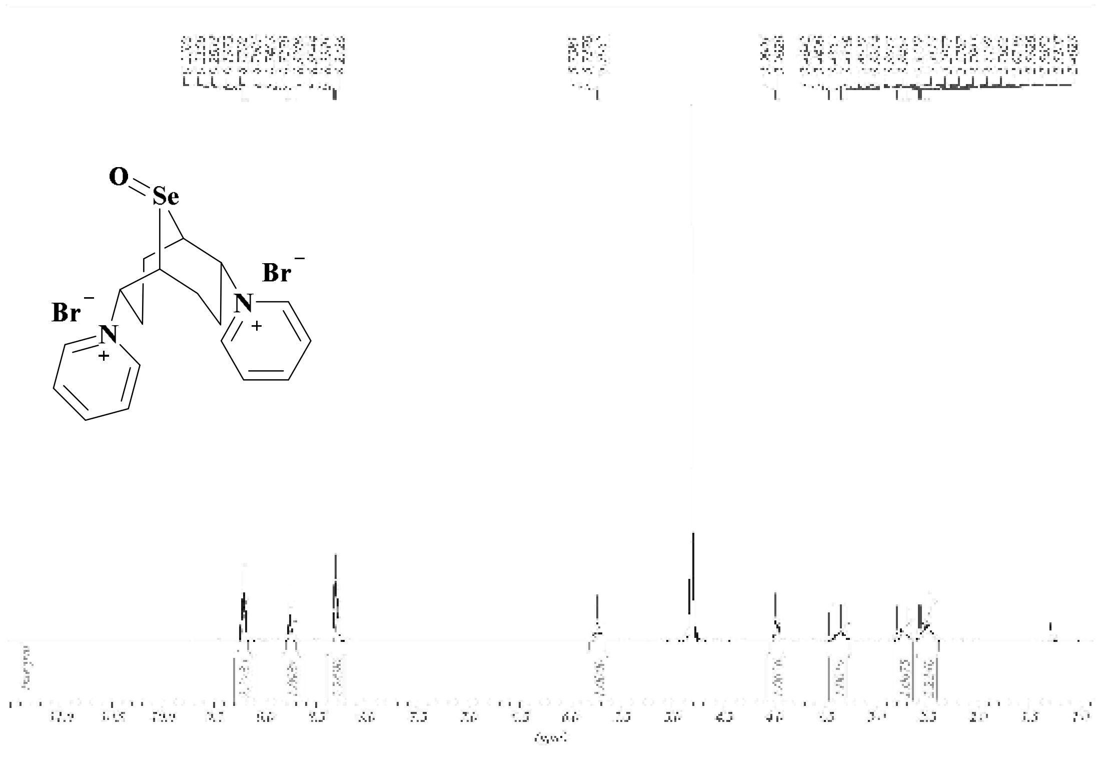

^{1}H-NMR spectrum of compound 25

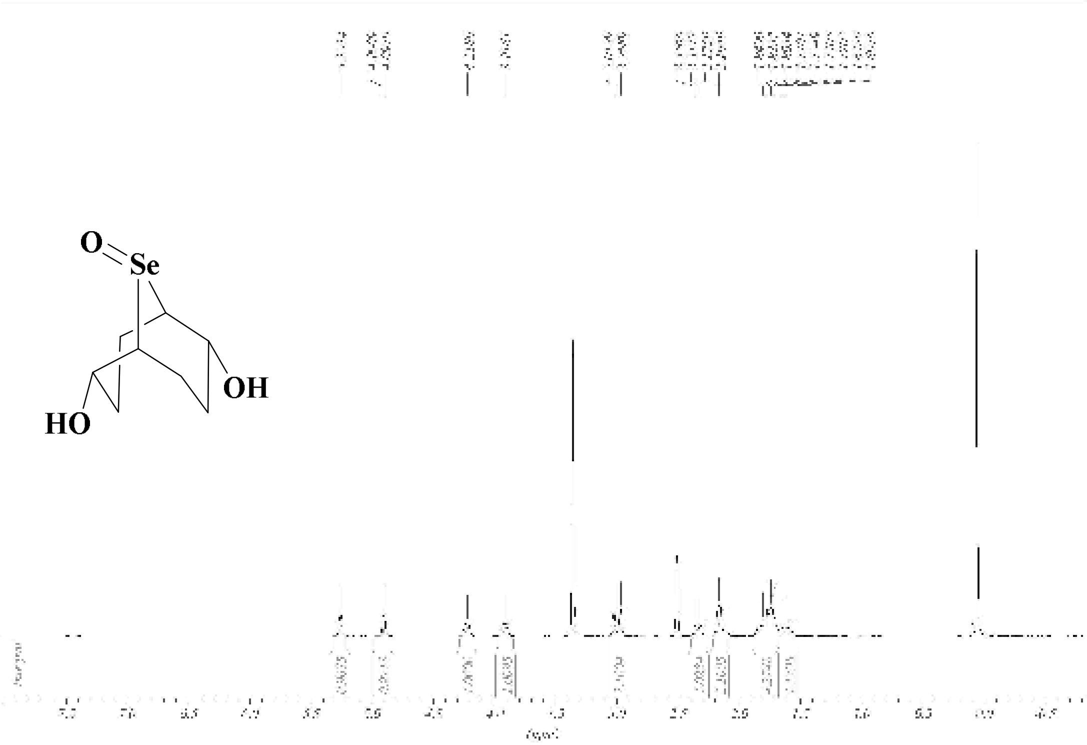

Examples of ^{13}C-NMR Spectra

^{13}C-NMR spectrum of compound 3 (D_2O)

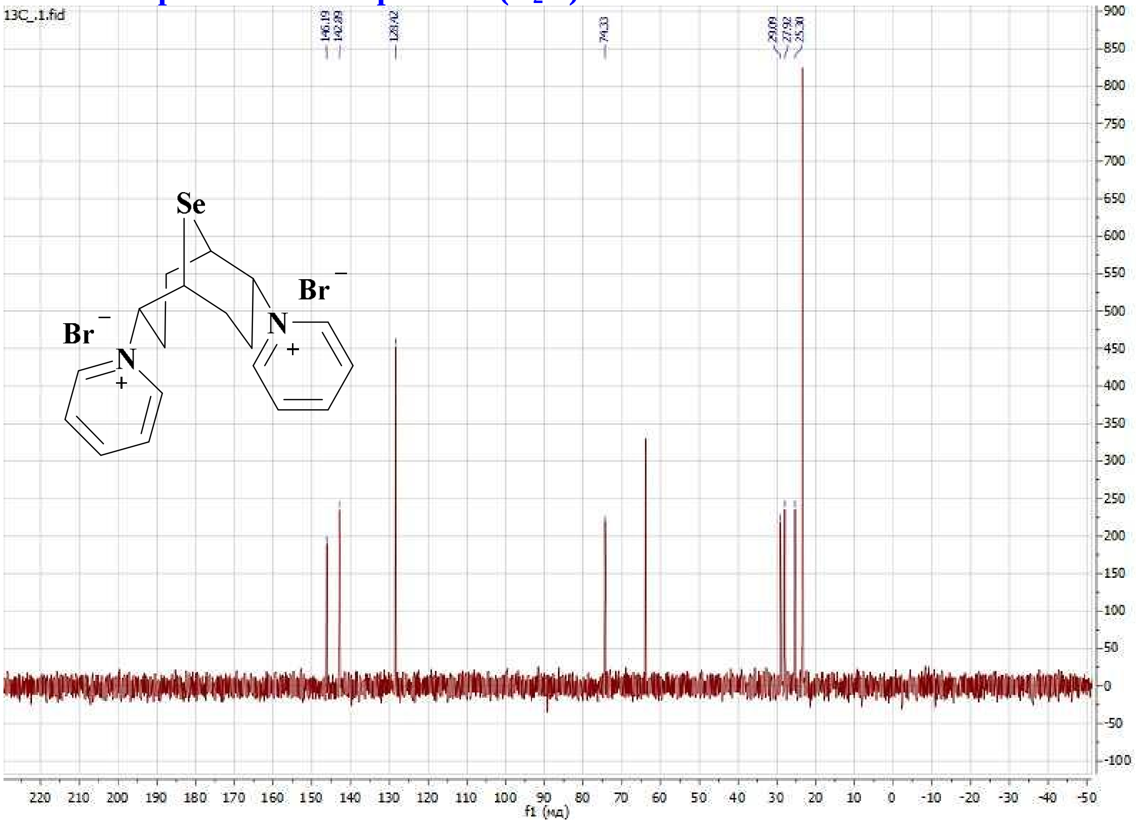

^{13}C-NMR spectrum of compound 4

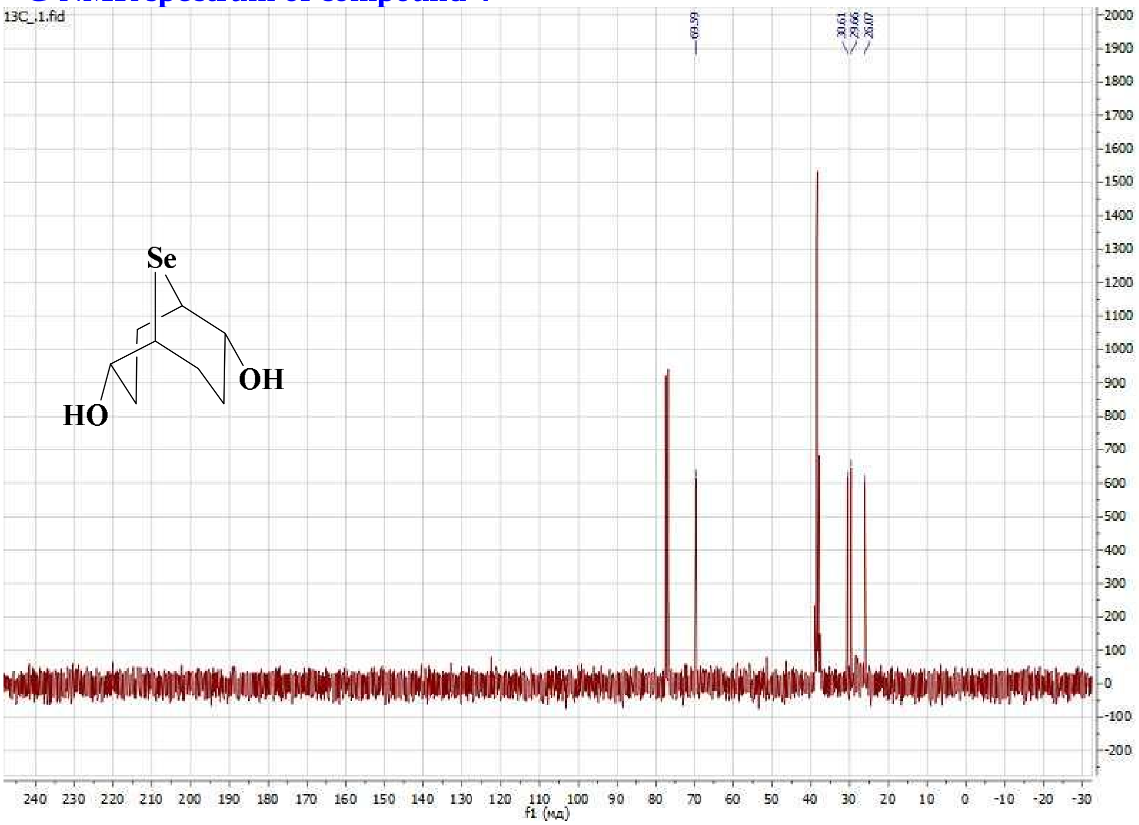

^{13}C-NMR spectrum of compound 5

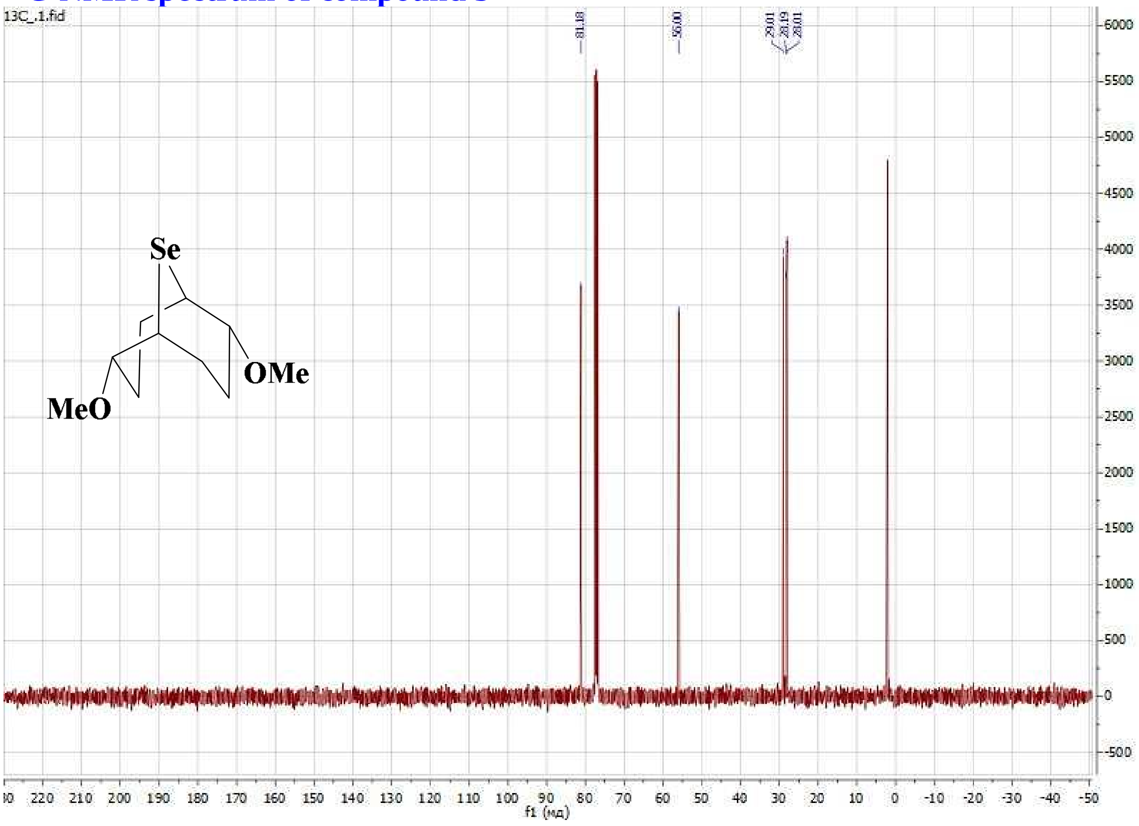

^{13}C-NMR spectrum of compound 6

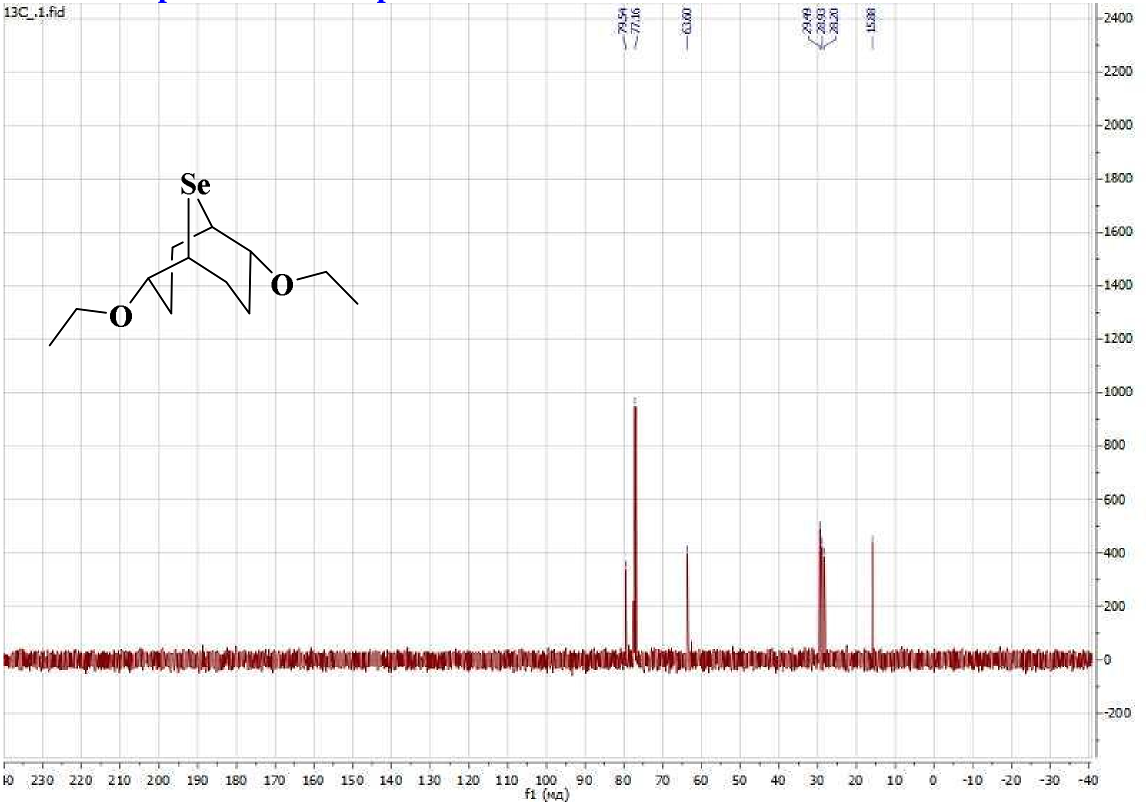

^{13}C-NMR spectrum of compound 7

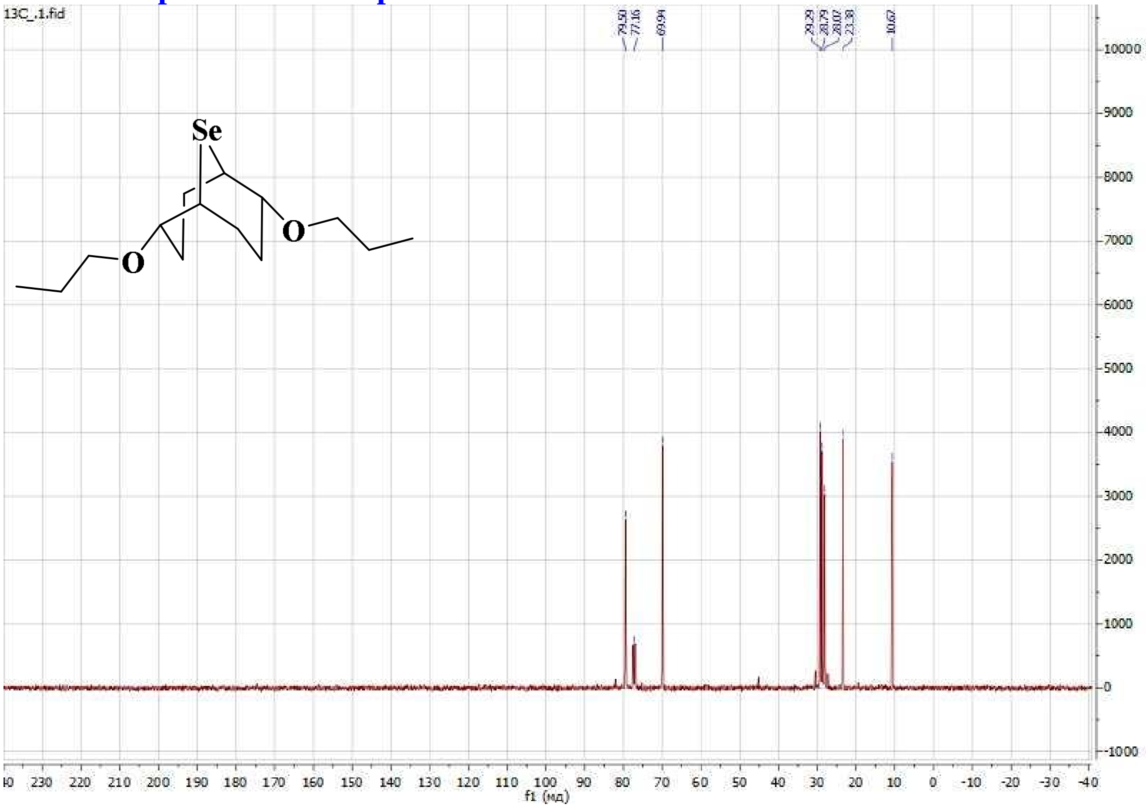

^{13}C-NMR spectrum of compound 8

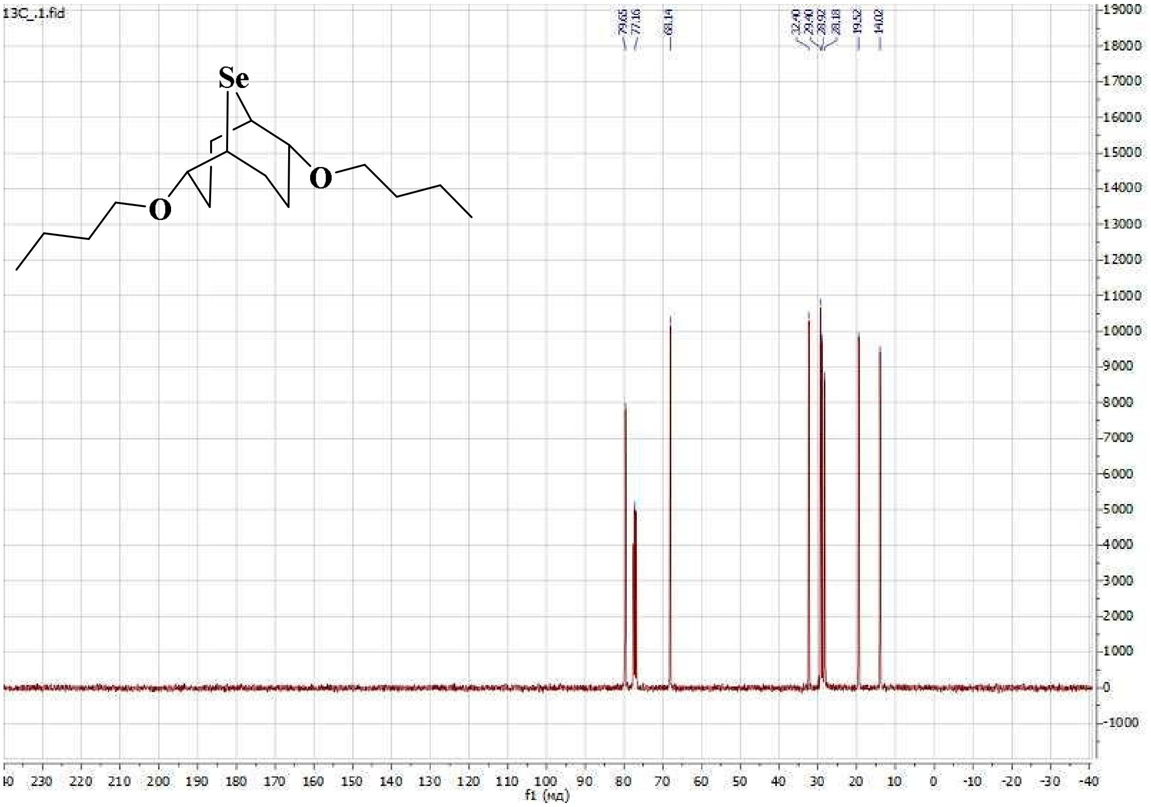

^{13}C-NMR spectrum of compound 9

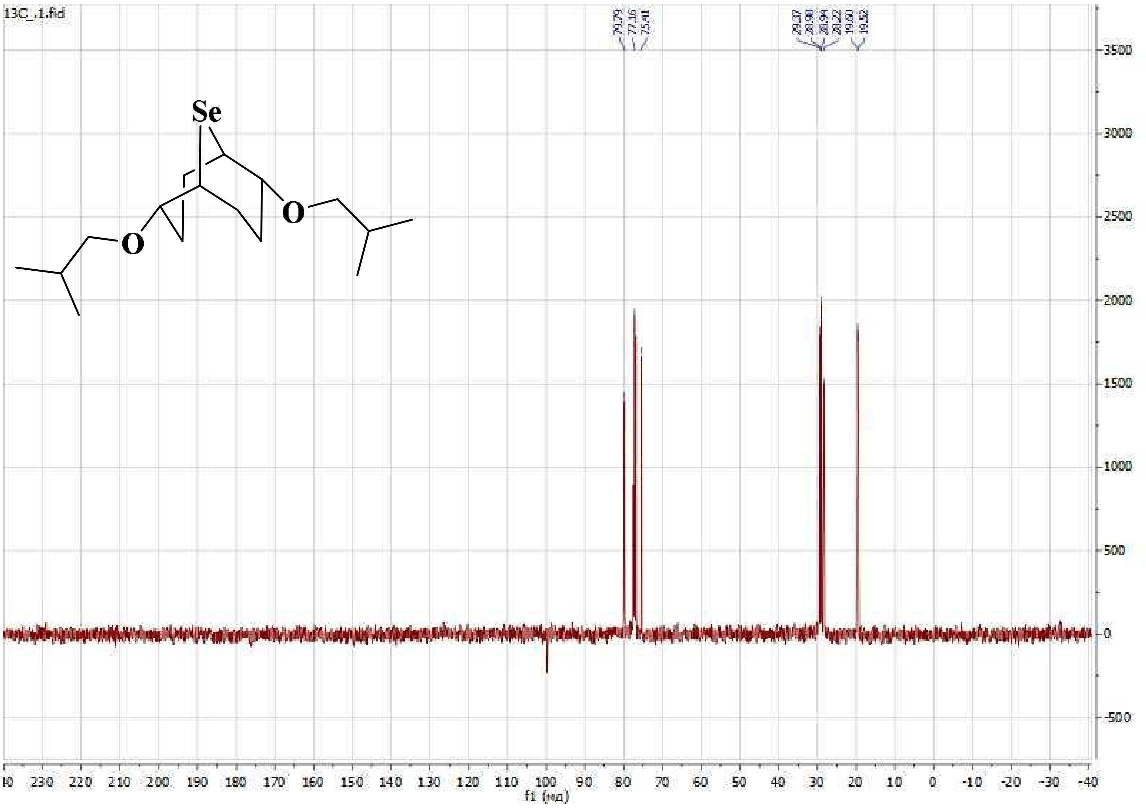

^{13}C-NMR spectrum of compound 10

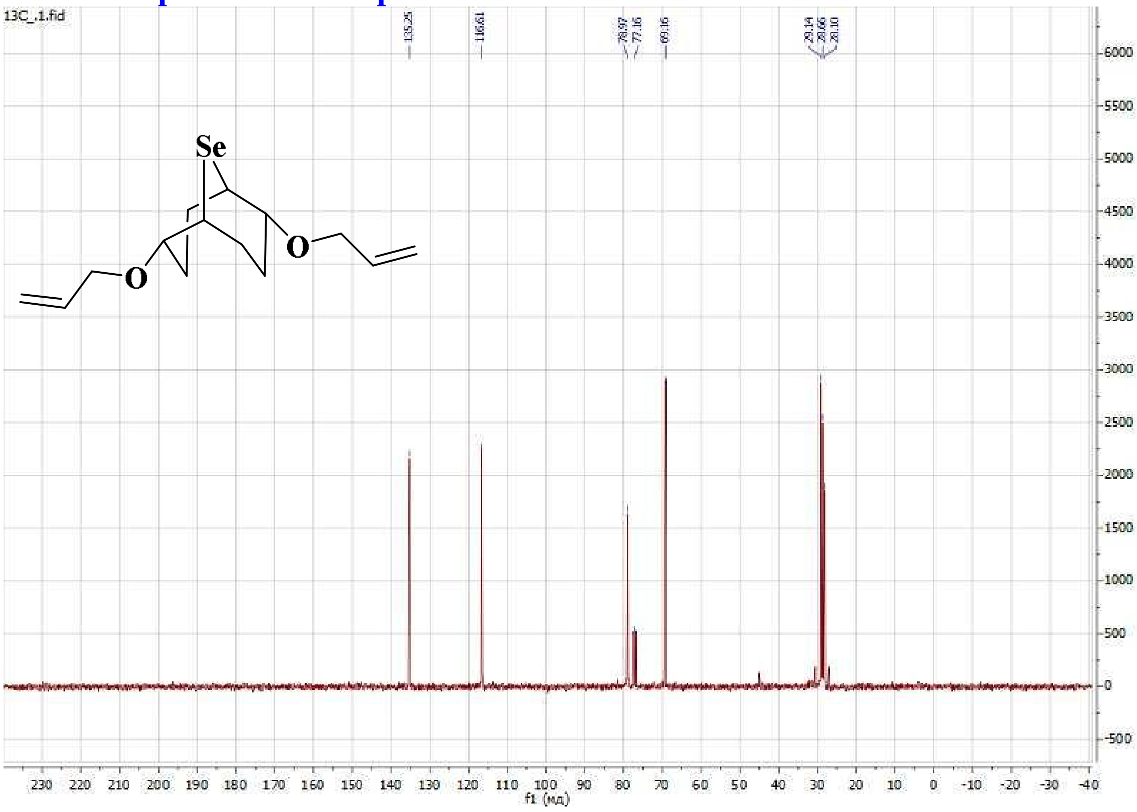

^{13}C-NMR spectrum of compound 11

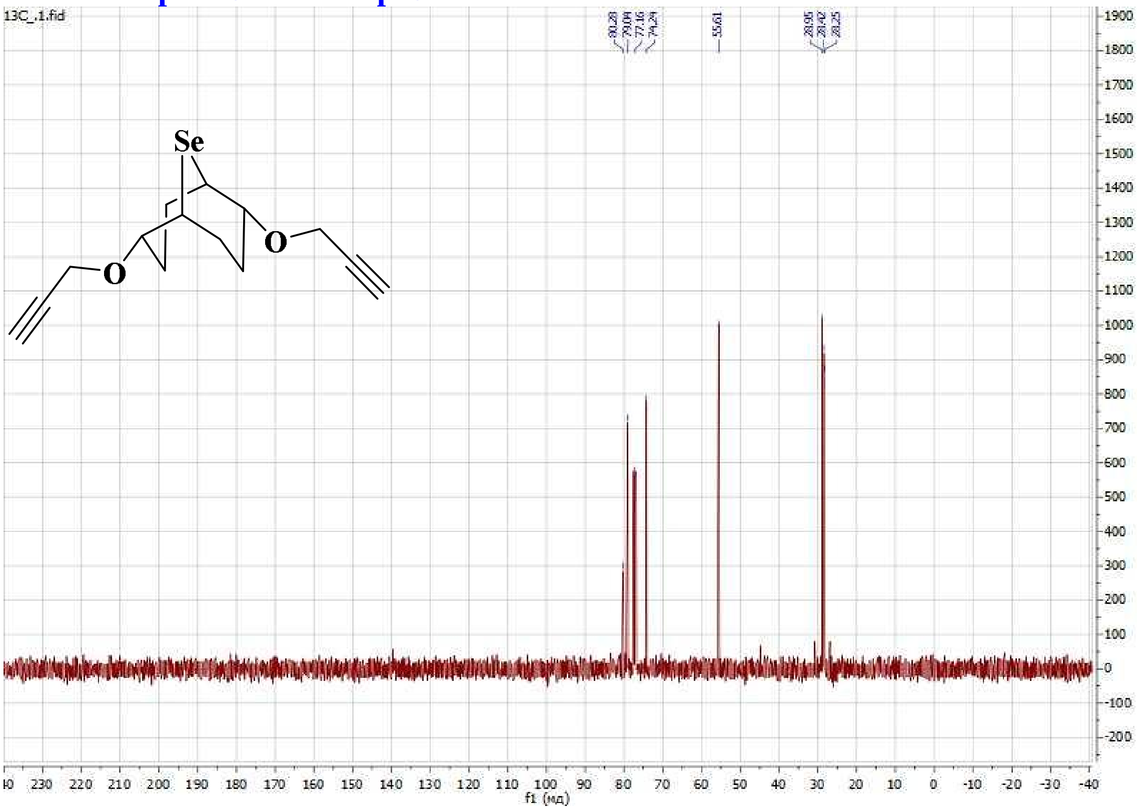

^{13}C-NMR spectrum of compound 12

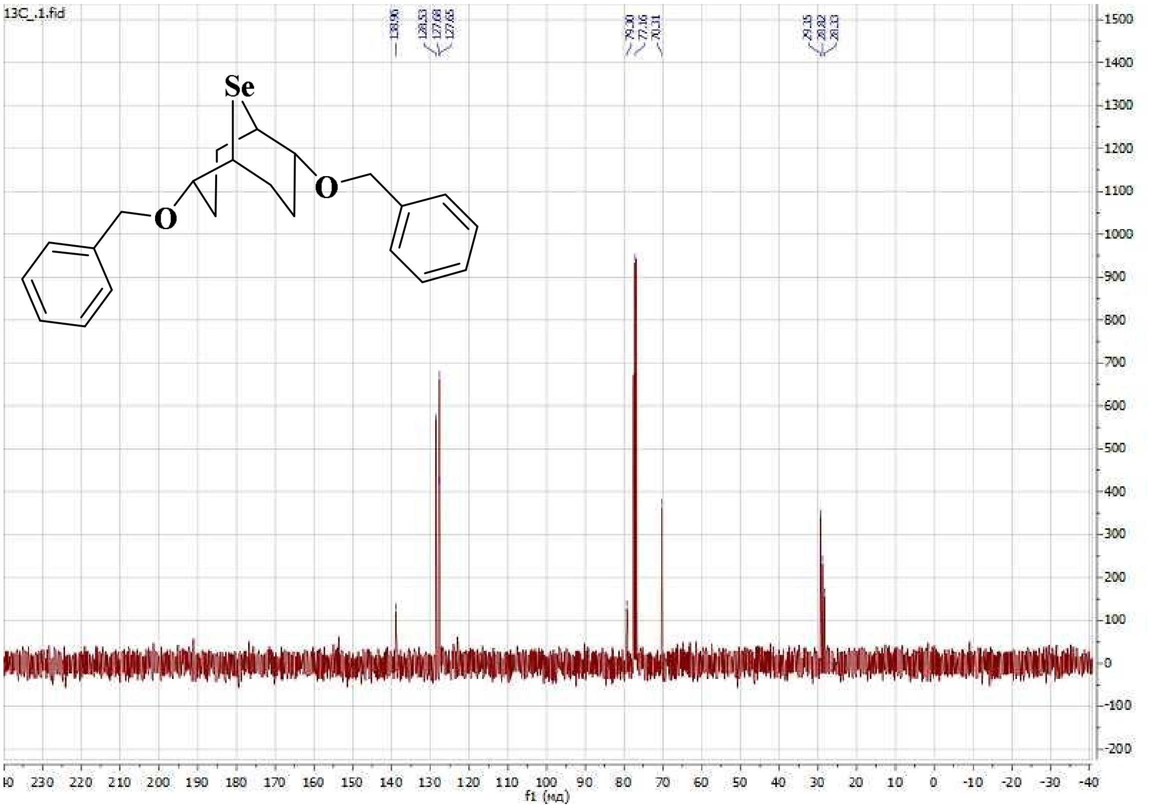

^{13}C-NMR spectrum of compound 13

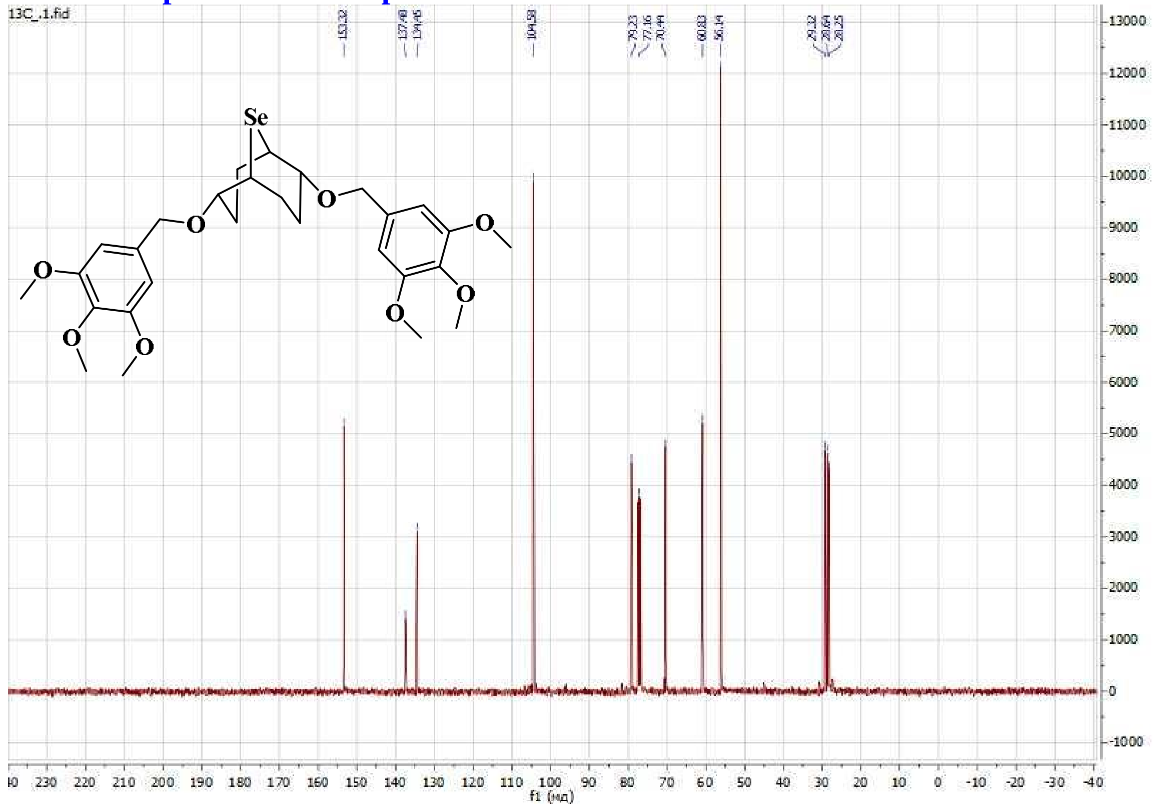

^{13}C-NMR spectrum of compound 14

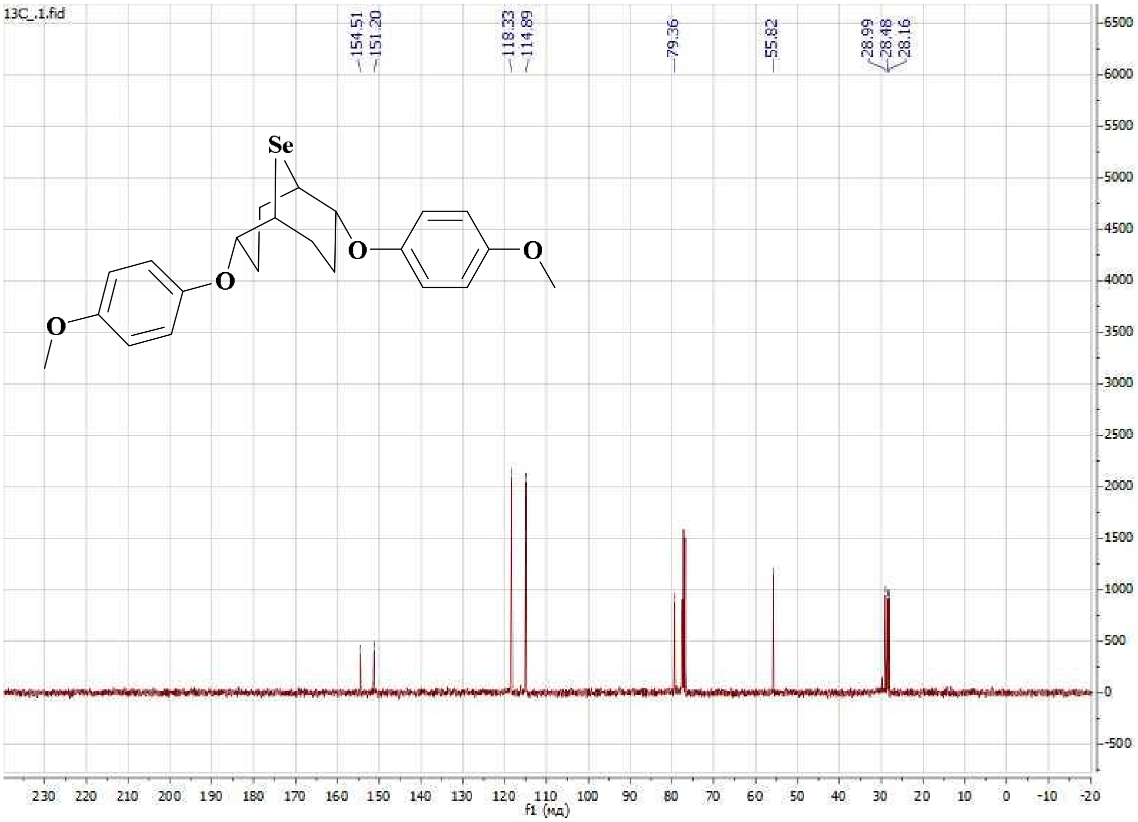

^{13}C-NMR spectrum of compound 15

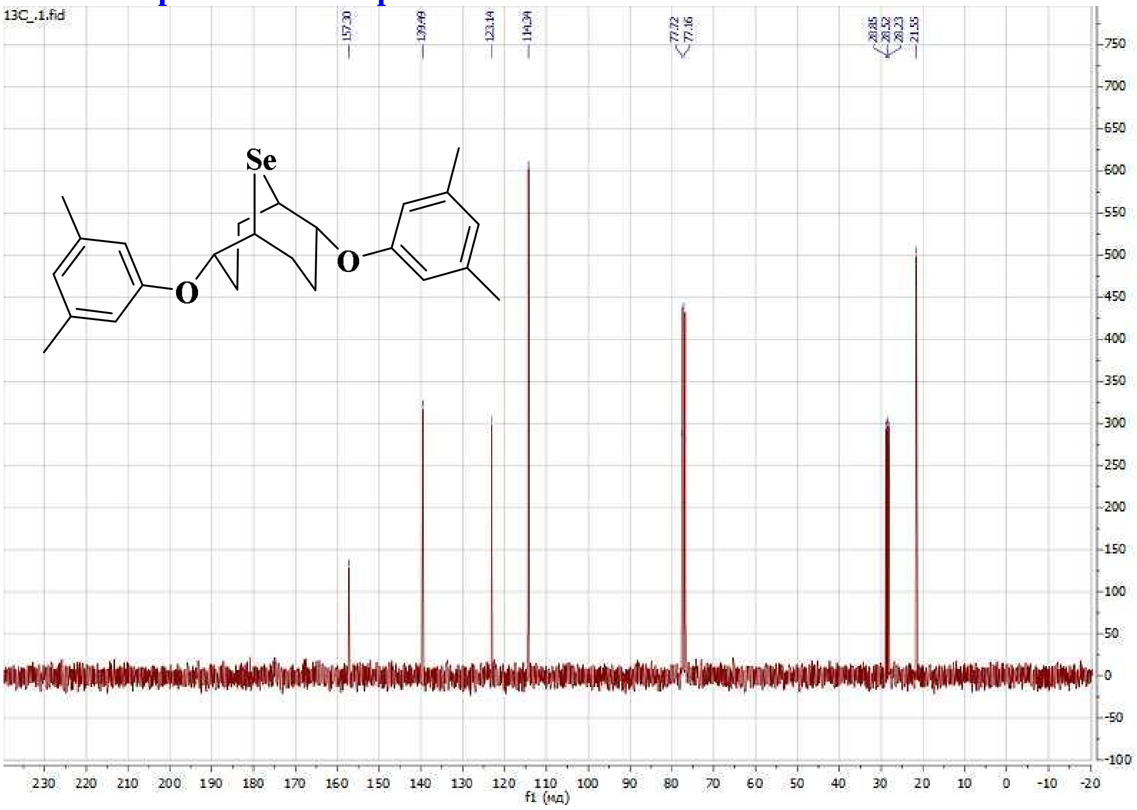

^{13}C-NMR spectrum of compound 16

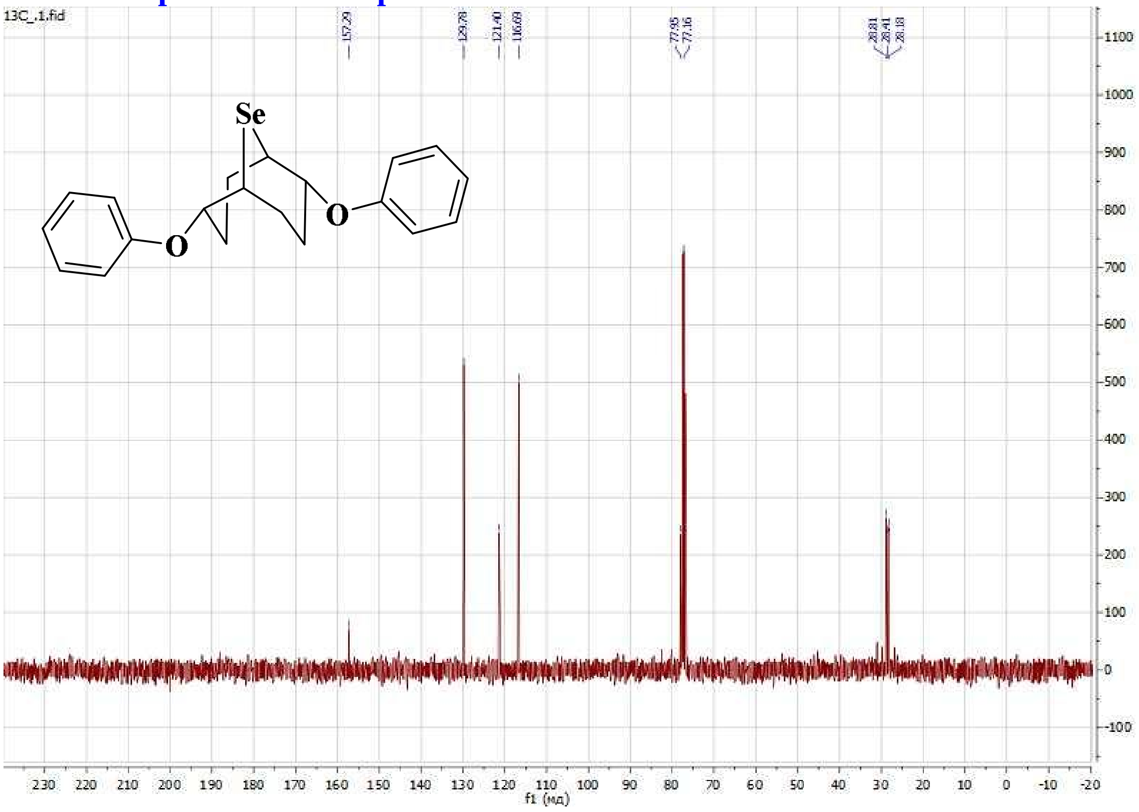

^{13}C-NMR spectrum of compound 18

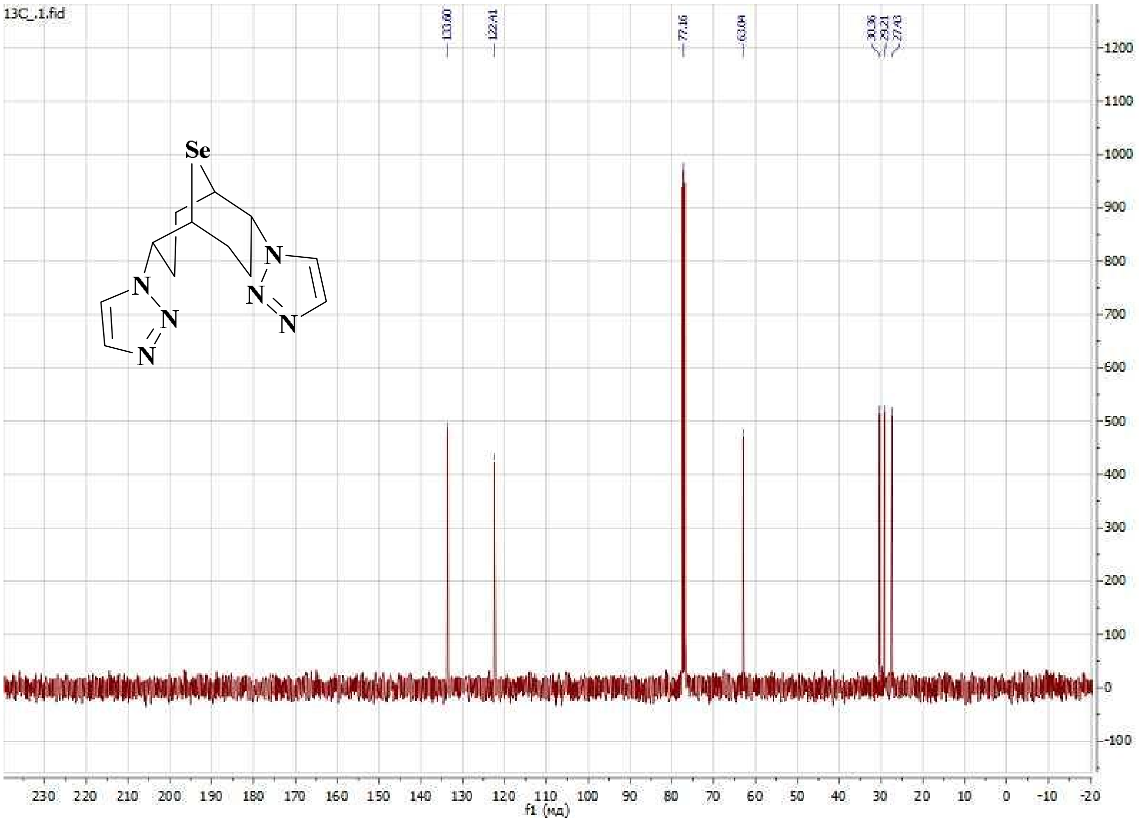

^{13}C-NMR spectrum of compound 19 (DMSO-d_6/$CDCl_3$)

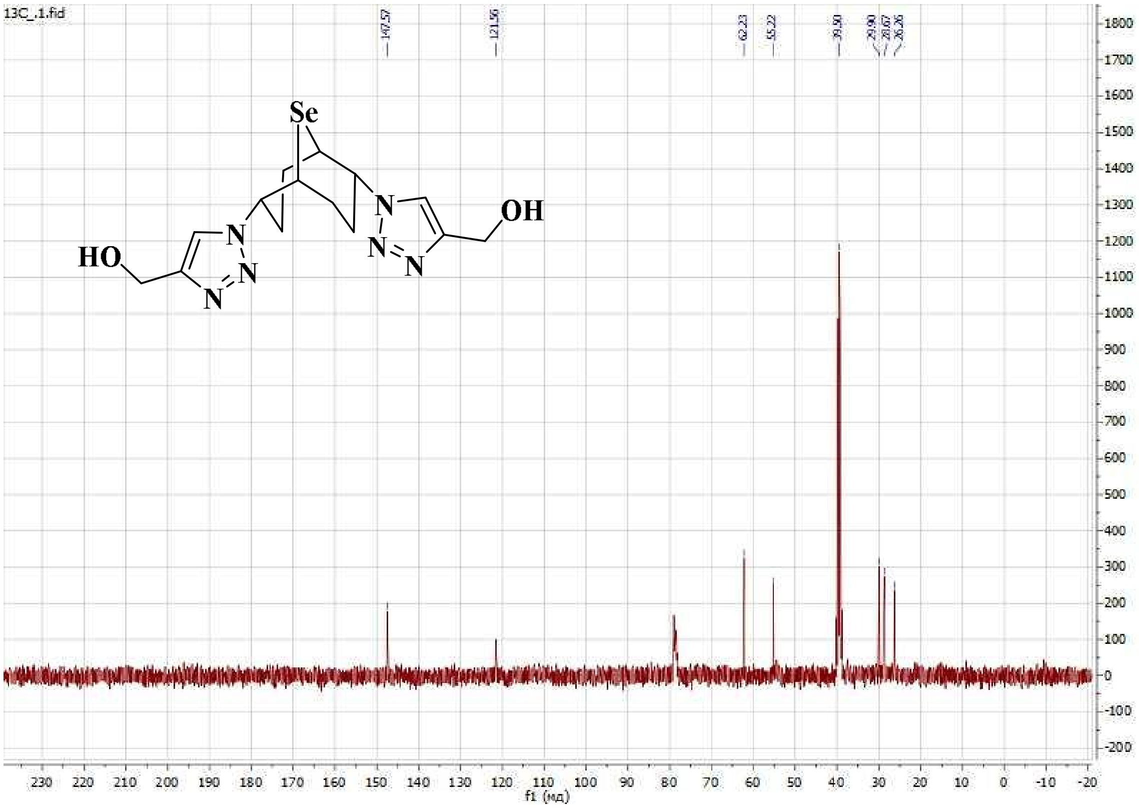

^{13}C-NMR spectrum of compound 22

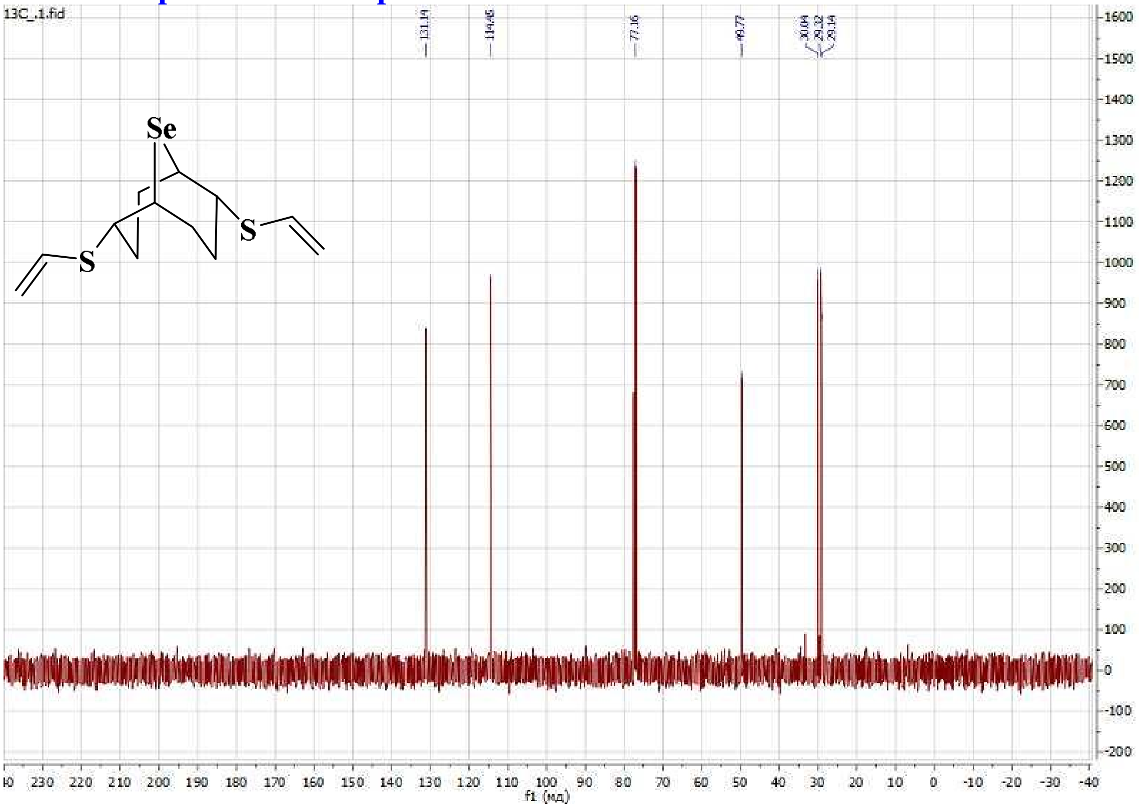

^{13}C-NMR spectrum of compound 23

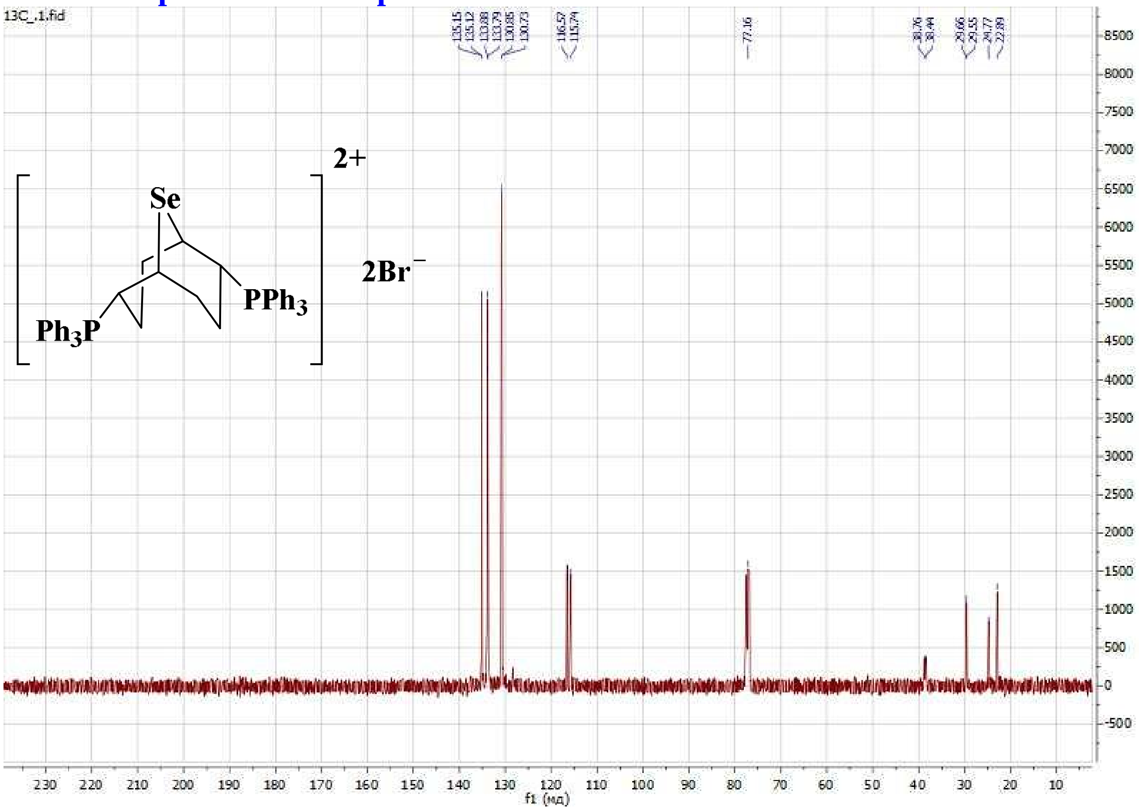

^{13}C-NMR spectrum of compound 24

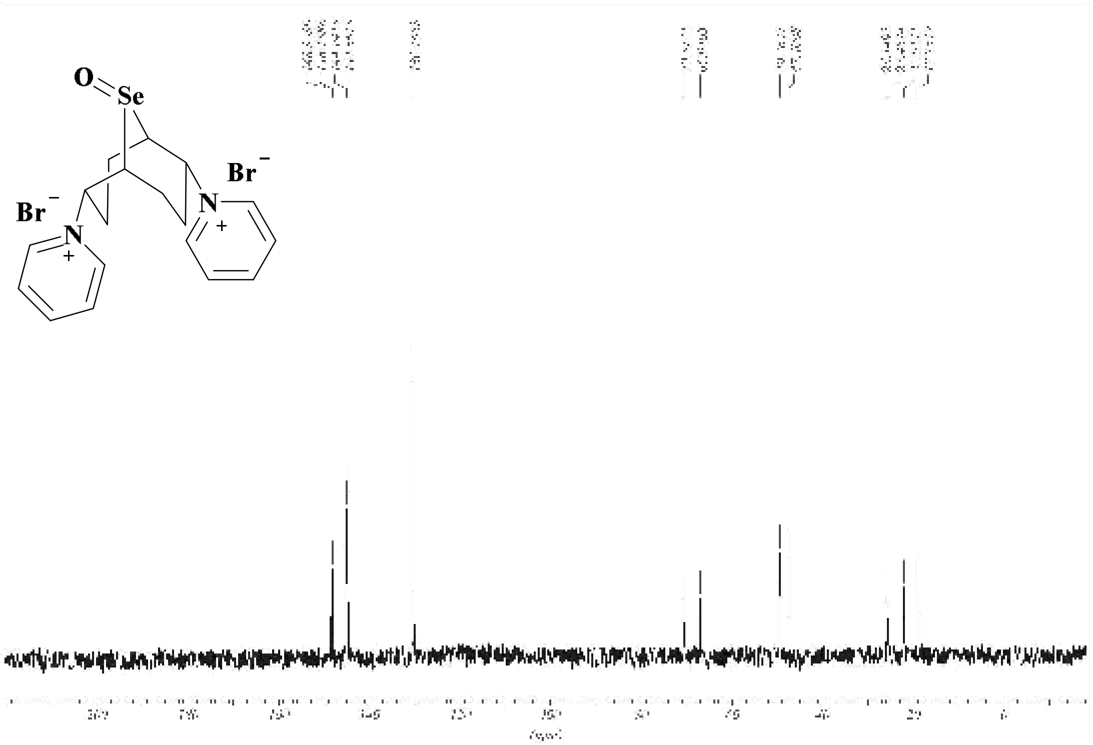

^{13}C-NMR spectrum of compound 25

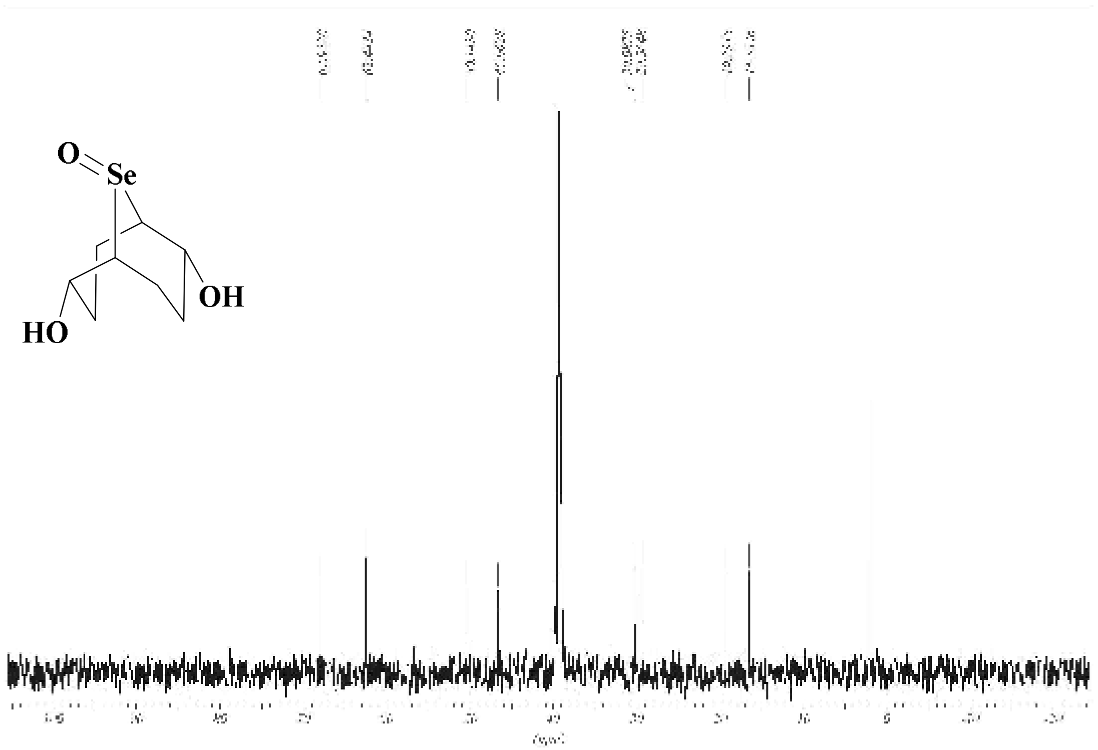

Examples of ^{77}Se-NMR and ^{1}H-^{77}Se-HMBC NMR Spectra

^{77}Se-NMR spectrum of compound 4

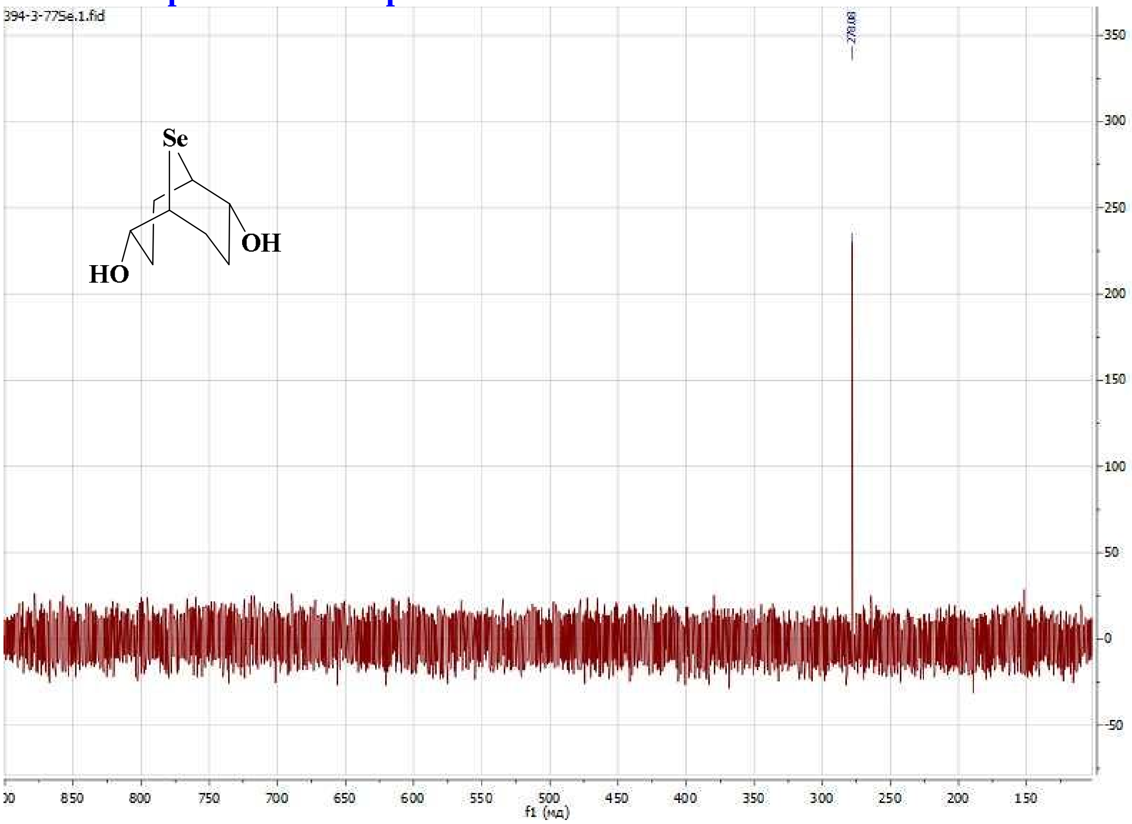

^{1}H-^{77}Se-HMBC NMR spectrum of compound 6

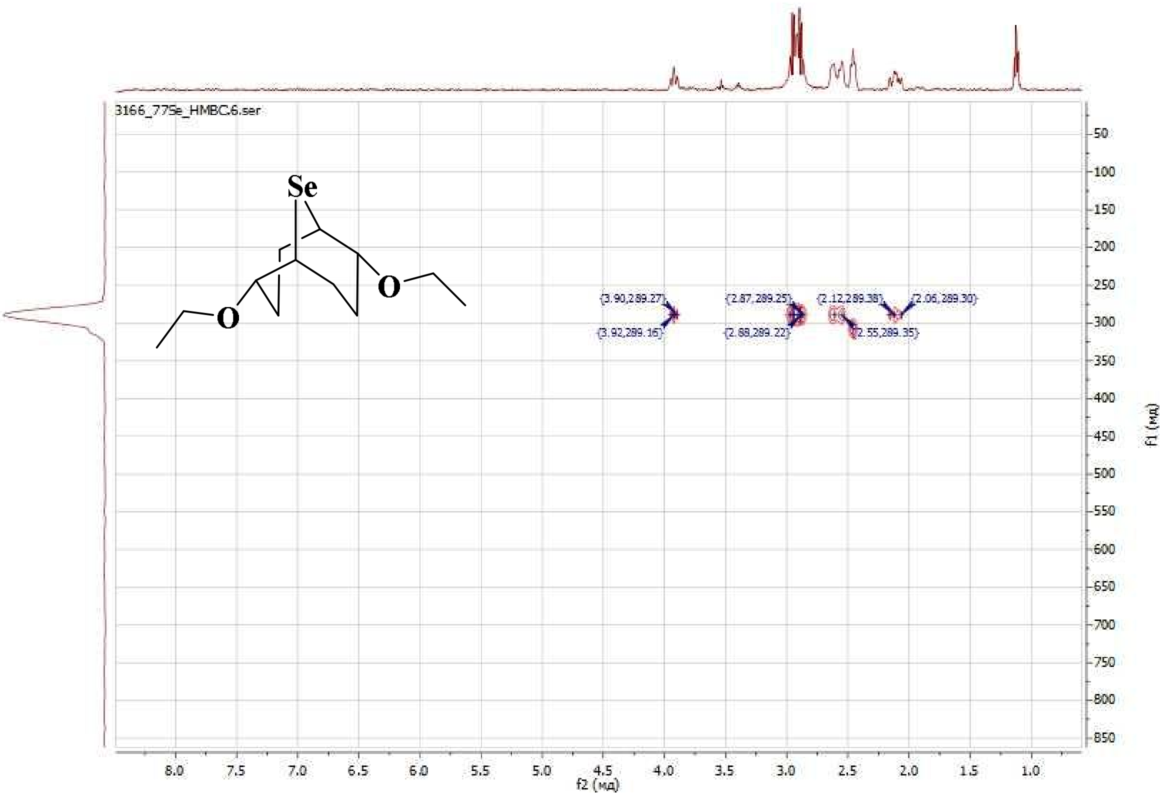

^{1}H-^{77}Se-HMBC NMR spectrum of compound 7

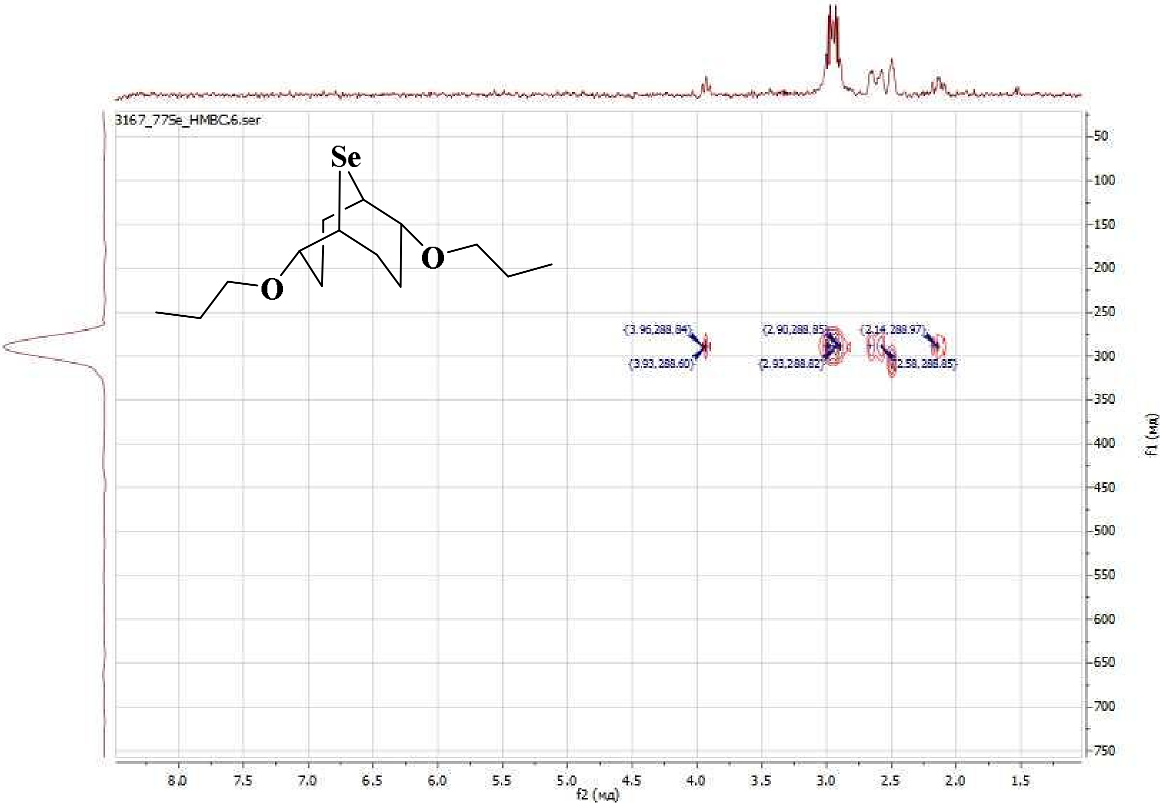

^{1}H-^{77}Se-HMBC NMR spectrum of compound 10

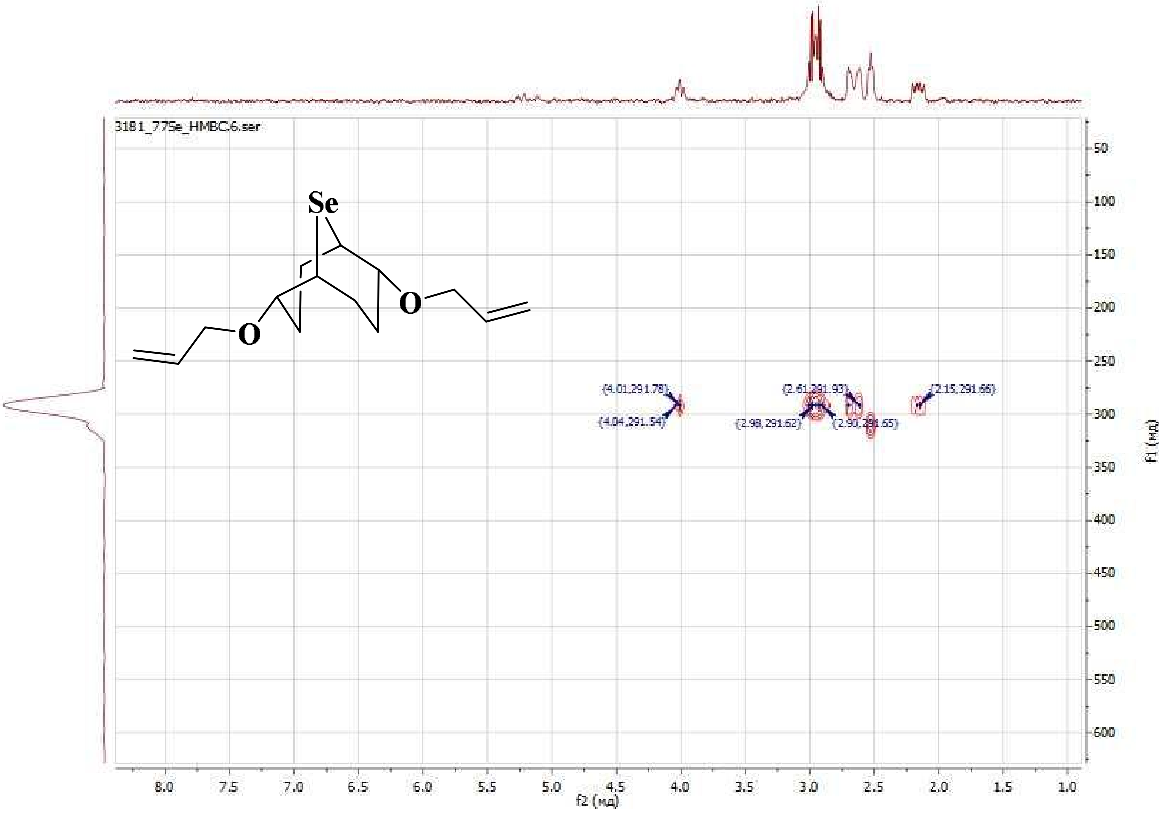

^{1}H-^{77}Se-HMBC NMR spectrum of compound 11

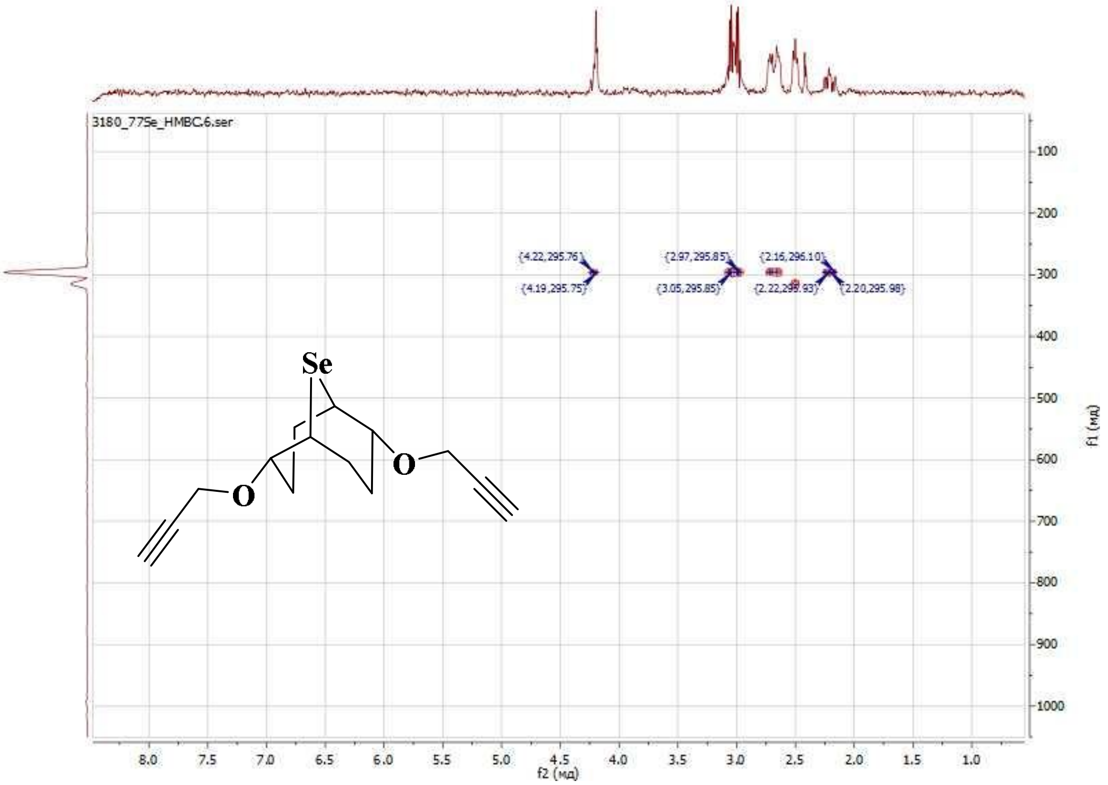

^{1}H-^{77}Se-HMBC NMR spectrum of compound 19

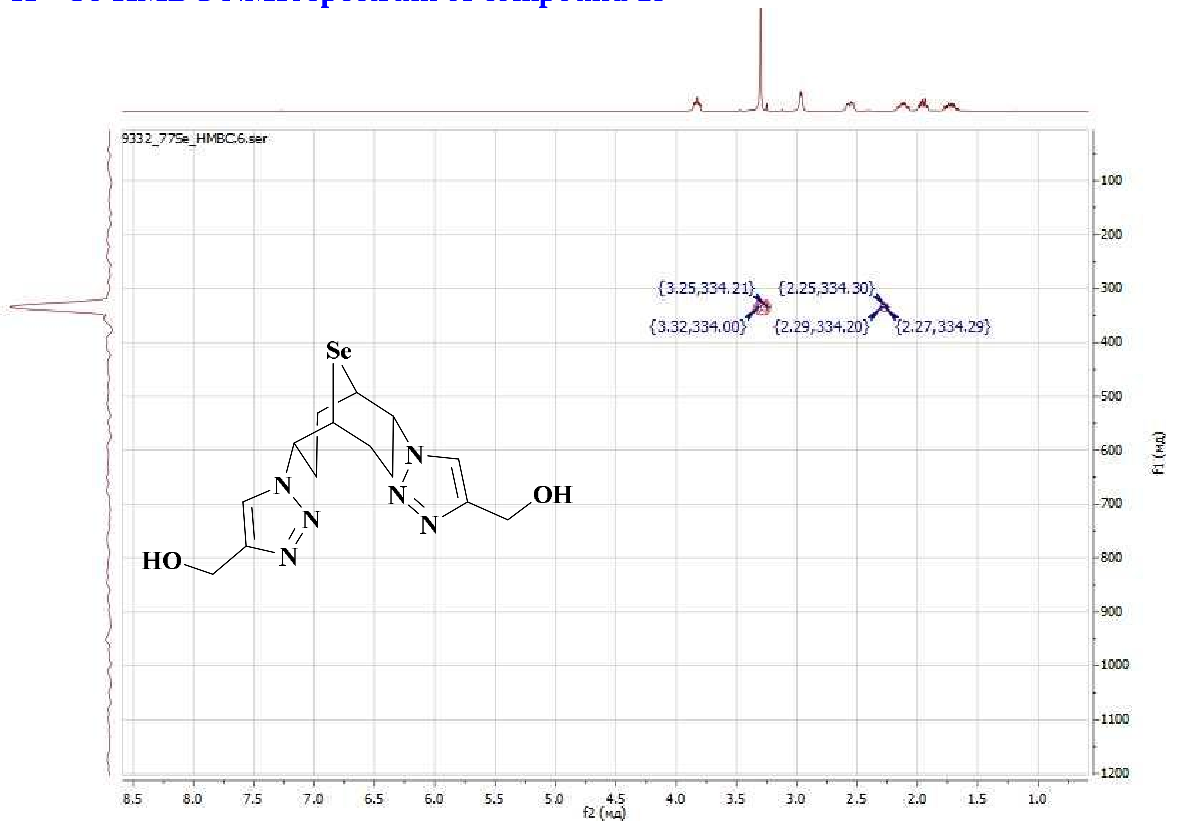

^{77}Se-HMR spectrum of compound 25

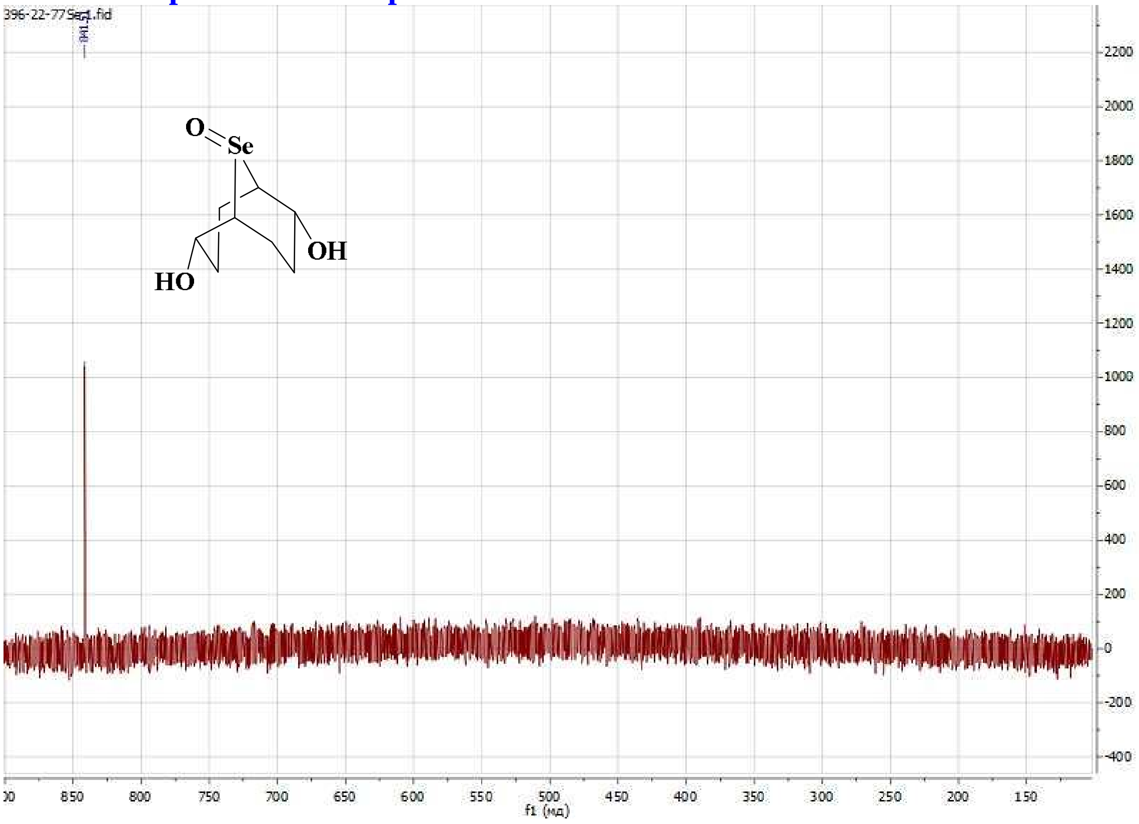

Examples of Mass Spectra

Mass spectrum of bis(methoxy) derivative 5 (M^+ = 250)

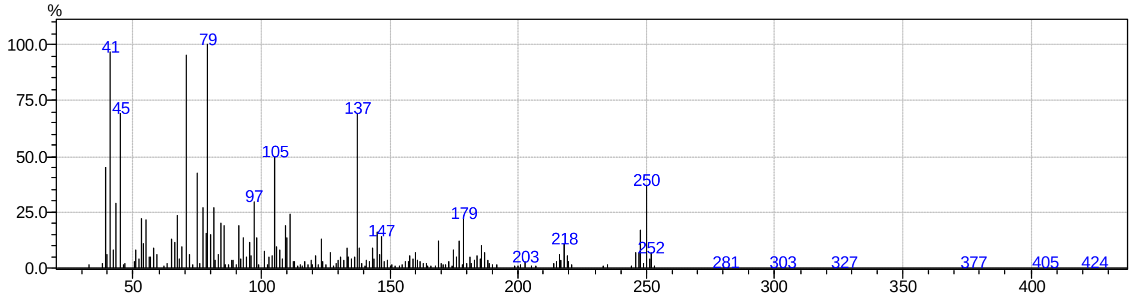

Mass spectrum of bis(ethoxy) derivative 6 (M^+ = 278)

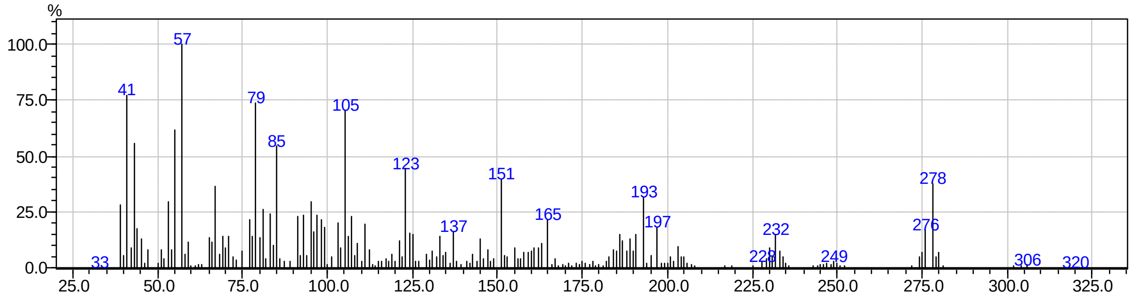

Mass spectrum of bis(propoxy) derivative 7 (M^+ = 306)

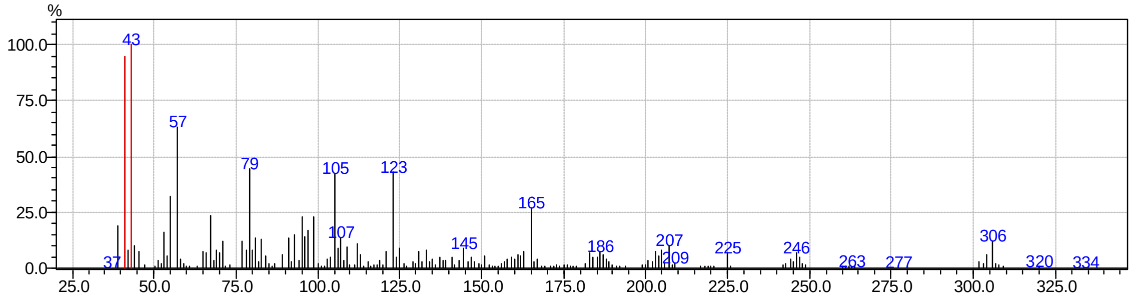

Mass spectrum of bis(butoxy) derivative 8 (M^+ = 334)

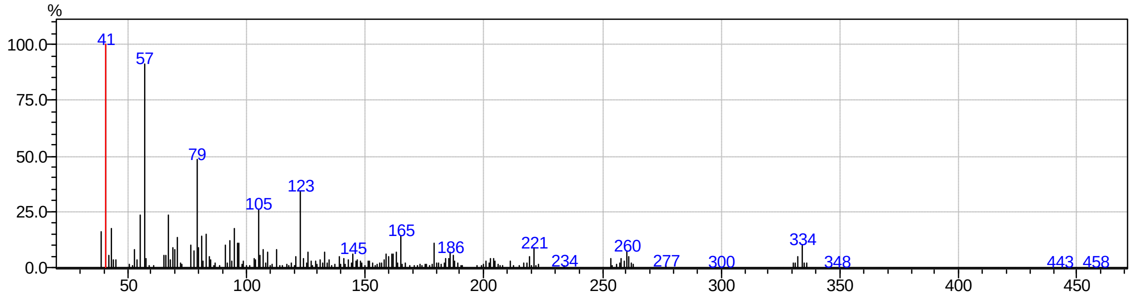

Mass spectrum of bis(isobutoxy) derivative 9 (M^+ = 334)

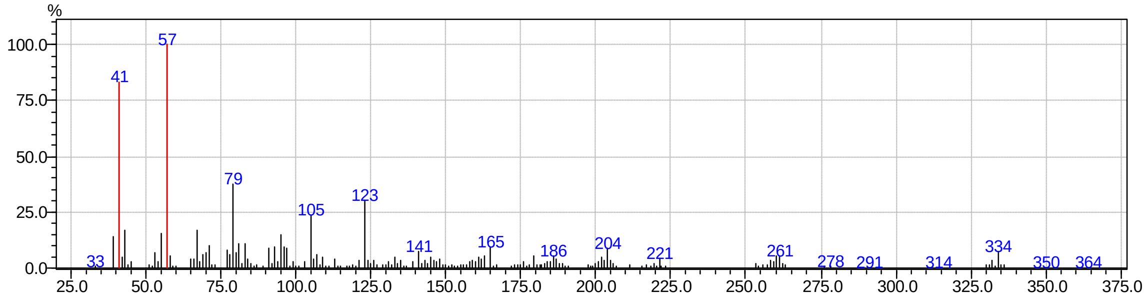

Mass spectrum of bis(allyloxy) derivative 10 (M^+ = 302)

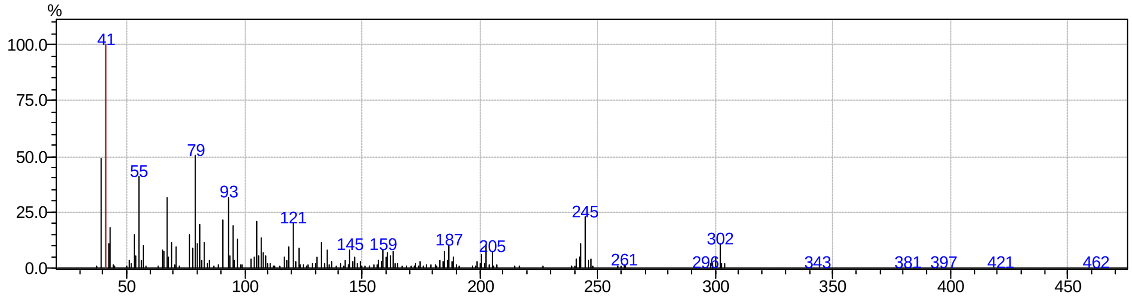

Mass spectrum of bis(propargyloxy) derivative 11 (M^+ = 298)

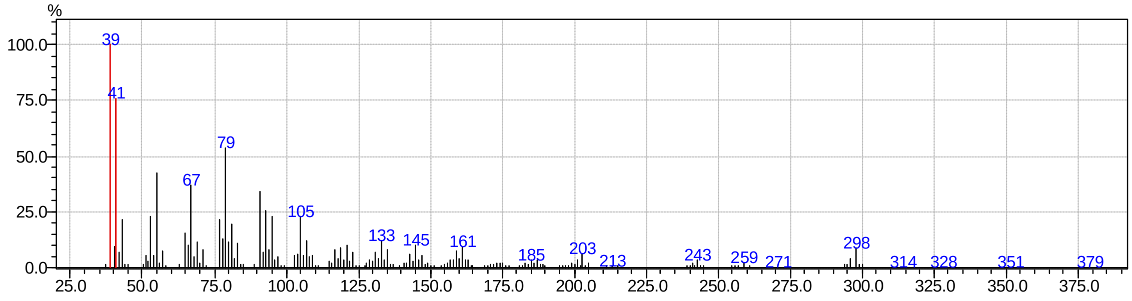

Mass spectrum of bis(4-methoxyphenyloxy) derivative 14 (311 = [M^+−$MeOC_6H_4O$])

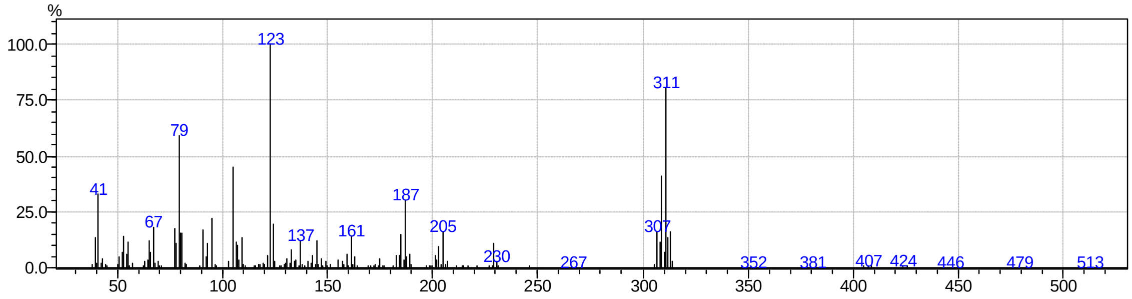

Mass spectrum of bis(3,5-dimethylphenyloxy) derivative 15 (309 = [M^+−$Me_2C_6H_3O$])

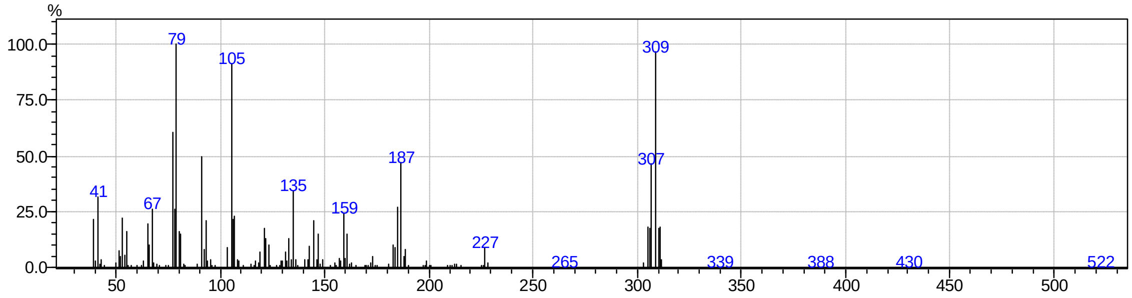

Mass spectrum of bis(phenyloxy) derivative 16 (281 = [$M^+ - C_6H_5O$])

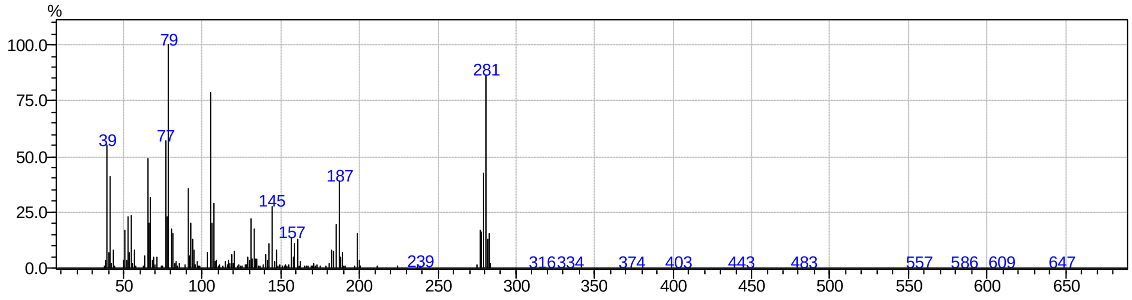

Supplement: Supplementary file 1 [file ijms-23-15629-s001.zip › ijms-2073245-supplementary.pdf]
